# Rapid screening of shellfish tainting from oil spills using an antibody-based biosensor

**DOI:** 10.1093/etojnl/vgae024

**Published:** 2025-01-06

**Authors:** Kristen M Prossner, Aaron D Redman, Christopher M Prosser, Thomas F Parkerton, Michael A Unger

**Affiliations:** Virginia Institute of Marine Science, Aquatic Health Sciences, William & Mary, Gloucester Point, VA, United States; ExxonMobil Biomedical Sciences, Inc., Annandale, NJ, United States; ExxonMobil Biomedical Sciences, Inc., Annandale, NJ, United States; EnviSci Consulting LLC, Austin, TX, United States; Virginia Institute of Marine Science, Aquatic Health Sciences, William & Mary, Gloucester Point, VA, United States

**Keywords:** polyaromatic hydrocarbons (PAHs), biosensor, tainting, oil spill, bivalves

## Abstract

Tainting of shellfish by polyaromatic hydrocarbons (PAHs) following an oil spill poses possible health risks as well as socioeconomic impacts. Traditional screening approaches for evaluating PAH contamination have limitations that can prevent timely, objective spill response decisions. The objective of this study was to investigate the relationship between PAH concentrations measured in the oyster, *Crassostrea virginica*, interstitial fluid using a rapid antibody-based biosensor method, with PAH concentrations in oyster tissues determined using conventional gas chromatography–mass spectrometry analysis. To accomplish this objective, bioconcentration tests were performed to simulate oil spill exposures using a crude and heavy fuel oil containing different PAH compositions. This design allowed both the PAH concentration and composition in water and, subsequently, accumulated by oysters to be varied over time. Oysters sampled during uptake and depuration phases were analyzed using biosensor and conventional analysis methods to generate comparative data. Results indicated that biosensor measurements of oysters captured the kinetics of PAH accumulation during uptake and depuration phases. Further, significant positive correlations were observed between biosensor interstitial fluid and lipid-normalized PAH tissue concentrations. However, quantitative predictions appear to be modulated by the contamination source and target analyte list for tissue analysis. Thus, the biosensor can be applied for rapidly evaluating relative PAH contamination between biota samples and offers a promising new analytical tool for oil spill monitoring and fisheries management contexts. A generic model was also developed from study and literature data to predict PAH half-lives from bivalve tissues. These predictions can help inform field monitoring of shellfish and estimate recovery times required to achieve pre-spill conditions.

## Teaser

Rapid detection of oyster tainting following an oil spill can greatly enhance response decisions.

Oysters exposed to Water Accommodated Fractions (WAFs) from two oils were analyzed using an antibody based biosensor and traditional GC-MS to evaluate uptake and depuration rates of PAHs.

A generic model was developed from study and literature data to predict PAH half-lives from bivalve tissues.

## Introduction

Global reliance on petroleum dictates that potential oil spills remain an ongoing priority for prevention and response. Crude oil and related refined products are composed of complex mixtures of various chemical classes, including aliphatic and aromatic hydrocarbons, resins and asphaltenes, and trace amounts of heterocyclic compounds containing nitrogen, oxygen, sulfur, or other metals. Polycyclic aromatic hydrocarbons (PAHs) are a key class of hydrocarbons in crude oil and refined petroleum products (e.g., fuel oils) that contribute to aquatic toxicity (McGrath & Di Toro, 2009). Polycyclic aromatic hydrocarbons are also the key focus of human health risk assessments at contaminated sites (Lee et al., 2021; Prossner et al., 2022; Qu et al., 2015).

Analytical techniques such as gas chromatography coupled with mass spectrometry (GC-MS) can provide quantitative compound-specific measurements of concentrations in various media (air, water, sediment, tissues) for calculating site-specific exposures. However, given the time and number of samples required for site characterization, this technique is often costly and not suitable for rapid screening (Christensen & Tomasi, 2007; Felemban et al., 2019; Mastovska et al., 2015; Mauseth & Challenger, 2001; Zhang et al., 2018).

Due to the sessile nature and lower ability to metabolize PAHs (James, 1989) bivalves have been shown to exhibit the highest tissue concentrations across contaminated sites (Replinger et al., 2017). In fact, PAH levels observed in bivalves are among the highest observed in all food products (European Food Safety Authority, 2008). Following a PAH contamination event, such as an oil spill, exposure via consumption of commercially important shellfish can pose a public health threat (Loh et al., 2017). Further, with *Crassostrea virginica* commercial fishery landings garnering over $140 million in the United States alone (National Marine Fisheries Service (NMFS), 2015), contamination-related fishery closures can translate into substantial socioeconomic impacts. Therefore, given these concerns, critical management questions following an oil spill are, has shellfish tainting occurred, and if so, when will tissue concentrations return to acceptable levels to allow reopening of the fishery or resumption of aquaculture harvesting?

Several seafood screening techniques exist to prioritize samples for further GC-MS analysis. Sensory analysis has been used following major spills, including the *Deepwater Horizon* incident in 2010 (Moller et al., 1999; US Food and Drug Administration, 2010). Sensory analysis is conducted by a panel of specialists trained to detect tainting of seafood by crude oil or petroleum products. The results of such tests indicate the presence of an altered seafood odor or taste. Although there is some evidence that a correlative relationship between taint and petroleum concentration may exist, specific compounds have yet to be attributed to seafood taint and thresholds may be related to specific species, oil type, or other unknown factors (Yender et al., 2002). Additionally, such testing may yield unreliable results depending on the sensitivity and experience of the sensory panel and factors unrelated to oil contamination (e.g., fecal odor and putrefaction) that can adulterate the sample (Mauseth & Challenger, 2001). Furthermore, sensory analysis cannot provide quantitative concentrations of toxic compounds, such as PAHs. Following the *Deepwater Horizon* event, the commercial fishery was reopened based on sensory panel testing and confirmatory chemical analysis of finfish and shrimp, which indicated no contamination. However, tar balls were subsequently encountered in deep water shrimp trawling nets during the next 2 weeks. A preliminary evaluation by a sensory panel indicated taint in some samples of captured royal red shrimp. This finding resulted in the National Oceanic and Atmospheric Administration closing 4,213 mi^2^ (10,911 km^2^) to royal red shrimp fishing. However, subsequent PAH analysis indicated no evidence of contamination. In a follow-up study with a larger sample size of field collected shrimp and an expanded sensory panel, no taint was detected, as confirmed by PAH tissue analysis (Shigenaka et al., 2015). The need to collect and analyze these samples for potential tainting resulted in an additional 6 weeks of fishery closure for shrimp.

Another rapid screening technique currently available is high performance liquid chromatography coupled with UV fluorescence detection (HPLC-FLD). This approach uses a more streamlined sample processing protocol than GC-MS; thereby improving sample throughput; however, the results provided by HPLC-FLD are semiquantitative estimates of concentrations. Thus, the sensitivity and accuracy of this analysis is also limited (Plaza-Bolaños et al., 2010, Yender et al., 2002). In addition, sample processing requires multiple steps, including solvent extraction, which limit its utility in remote settings because laboratory availability and specialized expertise are necessary (Gratz et al., 2011; Yender et al., 2002). Therefore, the need for improved, rapid, on-site screening analysis of PAH in tissues would serve as a valuable advance for prioritizing biota samples for quantitative analysis. Such an analytical tool would reduce the time and costs associated with spill monitoring and support more timely, objective response decision-making.

Monoclonal antibody–based immunoassays have proven to be valuable analytical screening tools for environmental monitoring of contaminants, including PAHs (Baeumner, 2003; Behera et al., 2018; da Costa Filho et al., 2022; Justino et al., 2017; Płaza et al., 2000; Spier et al., 2012; US Environmental Protection Agency [USEPA], 1996a, 1996b; Wang et al., 2014). A rapid, near real-time PAH quantitation method using a KinExA Inline Biosensor (Sapidyne Instruments, Boise, ID, USA) coupled with a murine anti-PAH monoclonal antibody, 2G8, has been demonstrated to have uniform selectivity for parent and alkylated PAH compounds in the 3- to 5-ring range (Li et al., 2016). Thus, this method captures the list of carcinogenic PAHs of particular human health concern (USEPA, 1993).

The biosensor method has been used to directly analyze PAHs in porewater samples (i.e., minimal sample preparation required) from Chesapeake Bay tributaries and the Houston Ship Channel and has shown to correlate well with freely dissolved PAH concentrations determined via GC-MS using passive sampling methods (Camargo et al., 2022; Conder et al., 2021; Hartzell et al., 2017). The biosensor coupled with mAb 2G8 has also been used to measure PAH concentrations in oyster (*C. virginica*) interstitial fluid samples, which showed a strong positive association with GC-MS-derived PAH concentrations in tissue (Prossner et al., 2022). In that study, oysters were collected from the Elizabeth River, a system in which legacy creosote contamination is a major source of PAH contamination (Di Giulio & Clark, 2015). These authors concluded it was reasonable to assume steady-state conditions between internal interstitial fluid and tissue concentrations. Through a novel extension of the equilibrium partitioning theory, Prossner et al. (2022) demonstrated that PAH concentrations measured via biosensor in oyster interstitial fluid could be used to predict tissue concentrations for rapid human health risk assessment of PAH-contaminated oysters and cost-effective field tissue monitoring.

The primary objective of this study was to further explore the relationship between PAH concentrations measured in *C. virginica* interstitial fluid, using the rapid biosensor method, with PAH concentrations in tissues determined using conventional GC-MS analysis. This was accomplished by performing short-term controlled laboratory oyster bioconcentration tests involving both uptake and depuration phases of PAH to simulate dynamic oil spill exposures. Two tests were performed, using either a crude or heavy fuel oil that contained different PAH compositions. By exposing oysters to water accommodated fractions (WAFs) of the two different oils, this design allowed both the PAH concentration and composition in water and, subsequently, accumulated by oyster tissues to be varied over time. Using oysters collected during uptake and depuration phases of the bioconcentration tests, the utility of the biosensor technology was evaluated for screening PAH contamination in oysters compared with traditional GC-MS analysis of tissues. A secondary goal was to determine elimination rates and corresponding half-lives of individual PAHs in oysters to assess how quickly contaminated tissues depurate once oil exposure is terminated. These results were then compared with previous bivalve PAH depuration studies compiled from the literature with the goal of developing a generic model that can inform predicted recovery times of tainted tissues.

## Materials and methods

### Test oils

The two oils investigated in this study, Quintana Hoops crude oil blend and heavy fuel oil (HFO), were provided by ExxonMobil Biomedical Sciences, Inc. (Annandale, NJ). Total aromatic concentrations of each oil based on 56 quantified analytes were 21,860 and 145,291 mg/kg, respectively, with speciated PAH composition provided in online supplementary material Table S1. Relative percentages of 2, 3, and > 4 ring aromatic compounds (online supplementary material Figure S1) indicate HFO is enriched in heavier 3+ ring PAHs (86%) compared with the crude oil (24%). The Hoops oil composition is also enriched in monoaromatics (54%), which are absent in HFO (online supplementary material Table S1).

### Experimental design and WAF generation

**Figure 1.**
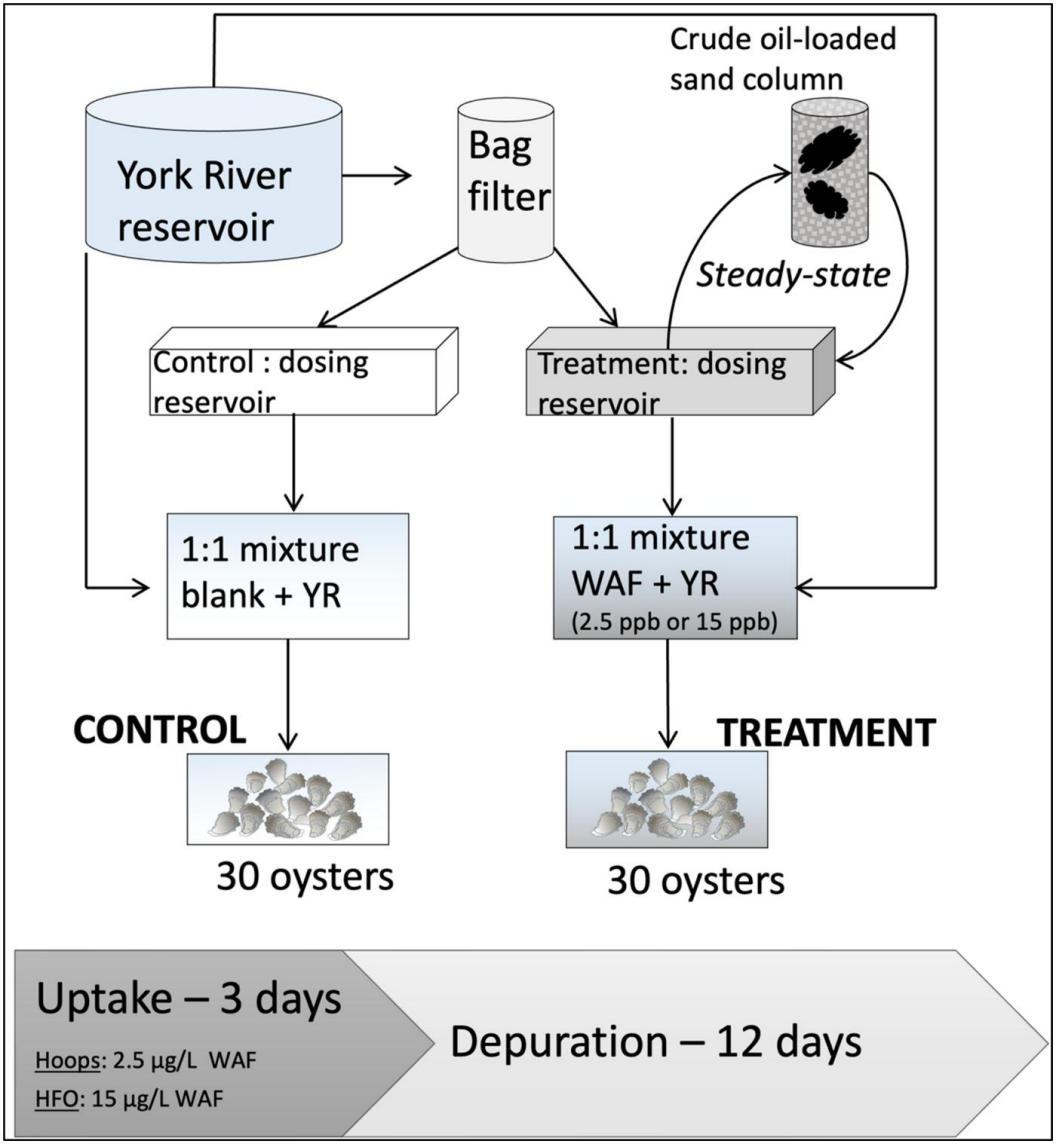
Schematic of experimental design. YR = York River water; WAF = water accommodated fraction of oil.

The experimental set-up was identical for the bioconcentration tests with the two oils investigated. Oysters were acutely exposed to oil WAFs and then allowed to depurate to evaluate the ability of the biosensor to predict tissue concentrations in the kinetic regime. The experiment was designed to simulate a short-term oil exposure characteristic of a spill in a controlled laboratory setting. The dosing system consisted of a flow-through system in which WAF (generated in a closed system via water recirculation through an oil-loaded generator column, described in following text) was pumped at 100 ml/min from the dosing water reservoir to the mixing aquarium, in which oil-dosed water and clean unfiltered York River water were mixed at a 1:1 ratio and delivered to the treatment tank. Approximately five test aquarium volume (∼30 L) exchanges occurred per 24-hr period. The purpose of mixing unfiltered York River water was to recreate realistic settings and ensure oysters would receive natural food, because supplementary food was not provided. The flow-through set-up for the control oysters was identical with the exclusion of WAF delivery to the tank. Figure 1 depicts a schematic of the experimental set-up. Oysters were exposed to WAF for 3 days. On the morning of the third day after a final uptake sample was collected, flow from the WAF dosing reservoir was stopped and residual water was siphoned out of the test aquarium. Tanks were refilled with clean, unfiltered York River water and volume exchange with fresh unfiltered water proceeded as previously described. The dosing reservoir and all tanks were covered to minimize loss of volatile hydrocarbons during WAF generation.

Water accommodated fractions were prepared as follows: 40 ml Quintana Hoops crude oil or HFO was loaded onto ignited filter sand in a 10 × 70 cm generator column. York River water was passed through a bag filter to remove particulate matter and recirculated for 11 or 12 days through the sand column and 310 L dosing water reservoir until apparent steady state concentrations were achieved, as determined by near real-time monitoring based on biosensor analysis of water samples as described in the section Biosensor analysis of water and oysters. When steady state was apparent, the generated WAF was retained in the dosing reservoir for use during the uptake phase. Samples for biosensor analyses of the undiluted WAF were collected twice per day for Hoops Oil and once per day for HFO tests.

### Oyster acquisition and husbandry

Adult cultured oysters were purchased from a local oyster farm on the York River in Gloucester County, VA. Oysters were transported to the laboratory (∼10 km away) in a cooler and kept cool with gel ice packs. Immediately upon arrival, oysters were scrubbed clean and shell height (bill edge to umbo) was measured by caliper prior to transfer to clean aquaria filled with unfiltered York River water. The oysters were acclimated to laboratory conditions for approximately 7 days prior to experimentation. Oysters (*n* = 35) were spread evenly along the bottom of each tank. At the start of the exposure, oysters were culled so that a total of 30 oysters were held in both the treatment and control tanks. Water quality parameters including temperature, salinity, pH, dissolved oxygen, and ammonia were monitored throughout each experiment in both the control and treatment tank and maintained at appropriate levels as per ASTM Standard Guide E1022-94 (ASTM International, 1994) using a digital aquarium tank thermometer, refractometer (for salinity), and commercial aquarium test kit.

### Sample collection

For sampling oil-dosed water from the generator column effluent, dosing reservoir, and test aquaria for biosensor analyses, aliquots of 10–15 ml water were collected in 20 ml glass screw-top scintillation vials and refrigerated at 4 °C until analysis. On the day of analysis, samples were filtered through a .45 µm PTFE syringe filter to remove larger particulate matter. During uptake, water samples were collected twice a day. During depuration, water samples were collected once a day. At the start of uptake, a 1 L sample of undiluted WAF from the generator column effluent used as dosing water for each bioconcentration test was also collected for GC-MS analysis in glass amber screw-top bottles. Samples were acidified using 4 ml of concentrated hydrochloric acid immediately upon collection and refrigerated at 4 °C until analysis to prevent possible microbial contamination.

For tissue sampling, oysters were retrieved from the tank using stainless steel tongs while minimizing disturbance of remaining test animals. Oysters were sampled from both oil and control treatments throughout the experiment. Whole oysters (i.e., in-shell) were placed in plastic bags and immediately frozen at –20 °C. During uptake, oysters were collected once a day. During depuration, oysters were collected every other day. Measurements in control samples and at time 0 in oil treatments reflect background PAH tissue concentrations in oysters prior to initiation and during the experiment. The first sampling of oysters occurred a few hours after the start of the exposure once the treatment tank was filled with WAF of each respective test oil. Prior to biosensor analysis, oysters were thawed, opened with a knife, and an aliquot of pooled fluid encompassing the soft tissue in the individual oyster (i.e., oyster interstitial fluid, per Prossner et al., 2022) was collected using a disposable glass Pasteur pipet. Fluid was collected from three individual oysters per sample period. The aliquot of individual oyster interstitial fluid was then transferred to a 20 ml glass scintillation vial for a final sample volume of approximately 5 ml, filtered through a .45 µm polytetrafluoroethylene (PTFE) syringe filter, and then frozen at –20 °C until analysis.

Following interstitial fluid sample collection, soft tissues from individual oysters were retained for GC-MS in glass screw-top jars and frozen at –20 °C until further processing. Soft tissues from the three oysters per sample (∼ 5 g [wet] of soft tissue per sample) period were pooled, homogenized, and then transferred to glass screw-top jars and stored frozen at –20 °C until overnight shipment of samples to the commercial lab for GC-MS and lipid analysis of the oyster tissues.

### Biosensor analysis of water and oysters

The procedures for biosensor analysis of PAH concentrations in water samples and oyster interstitial fluid (Camargo et al., 2022; Conder et al., 2021, Hartzell et al., 2017; Prossner et al., 2022; Spier et al., 2011) as well as selection and screening procedure of mAb 2G8 (Li et al., 2016) have been described previously. The murine monoclonal antibody mAb 2G8 has a uniformly strong affinity for 3-5 ring PAHs, including methylated compounds, measured in aqueous environmental samples with excellent correlation to GC-MS measurements (Li et al., 2016). There is some evidence that mAb 2G8 will bind with lower affinity to 2-ring aromatic hydrocarbons, particularly alkylated compounds (Conder et al., 2021); therefore, the total PAH concentration measured by the biosensor may be influenced by the presence of 2-ring aromatic compounds if present in high concentrations. The KinExA Inline Sensor (Sapidyne Instruments, Boise, ID) uses programmable fluidics to provide precise quantitative measurement of antibody binding for up to eight samples in a cycle. A fluorescent tag, AlexaFluor 647 (Invitrogen), was covalently bound to mAb 2G8 and the signal response (dV) of the fluorescently tagged antibody was measured by the KinExA detector. The KinExA Inline Sensor functions as a kinetic assay: the antibody is mixed with an aqueous sample and binds to free PAH, then the sample-antibody mixture is washed over stationary polymethyl methacrylate beads coated in antigen (1-pyrenebutyric acid, CAS no. 3443-45-6) conjugated to bovine serum albumin located in the detector flow cell. Any free mAb 2G8 that did not bind to dissolved PAH in the aqueous sample will bind to the antigen-coated beads and the resulting fluorescent signal then measured. Thus, the detected signal response is inversely proportional to the level of total PAH in a sample. The automated sample handling cycle takes up to 10 min to measure signal response in a sample and includes time for mixing with the antibody, cleaning of the flow cell, and replacement with a fresh set of antigen-coated beads for the next sample. At the start of a new day of operating the instrument and prior to sample analysis, a six-point calibration curve was generated using a laboratory blank, double deionized water (ddH_2_O), and a series of phenanthrene standards in ddH_2_O ranging from .5 to 2.5 µg/L to determine the linear range for the dV response from the instrument via log-linear regression analysis. Information on PAH standard sources is reported in online supplementary material Table S12. Once calibrated, samples were diluted accordingly with ddH_2_O to fall within the standard range, and the PAH concentration was then quantified based on measured signal response and required dilution factor. Concentrations are reported in micrograms per liter (ppb).

The detection limit for PAH analysis of oyster interstitial fluid by biosensor is reported to be .39 µg/L, as determined in Prossner et al. (2022). Signal responses detected outside the lower end of the calibration range (i.e., below the lowest phenanthrene standard, .5 µg/L) are reported as below the biosensor detection limit.

### GC-MS analysis of water

Gas chromatography coupled with mass spectrometry analysis of WAF generator column effluent for each experiment was conducted at the Virginia Institute of Marine Science following a standard procedure. The 1 L sample of dosing water was extracted with 3 aliquots of 100 ml of 100% dichloromethane. Surrogate PAH standards used for analysis included: d8-napthalene, d10-acenapthene, d10-phenanthrene, 1,1’ binapthyl, d12-chrysene, and d12-perylene. Polycyclic aromatic hydrocarbon standard sources are reported in online supplementary material Table S12. Calibration standards and samples were spiked with .1 ml of internal standard, p-terphenyl. A 7- to 10-point calibration curve was generated for analysis of individual PAH analytes and surrogate standards. A total of 64 analytes including both parent and alkylated compounds were measured with concentrations reported in micrograms per liter (online supplementary material Table S4).

### GC-MS and lipid analysis of oysters

Analysis of the oyster tissue was conducted by Alpha Analytical using GC-MS with selected ion monitoring based on a modified USEPA method 8270D (USEPA, 1998). Surrogate PAH standards used for analysis included d8-napthalene, d10-phenanthrene, and d12-benzo [a] pyrene. A total of 62 target analytes were examined, including parent and alkylated PAHs, several classes of thiophenes, and decalins, with results reported in micrograms per kilogram wet weight (online supplementary material Tables S5 and S6). Percentage of lipid content was also determined on all tissue samples using the method described in Lauenstein and Cantillo (1988) with results reported as percentage of wet tissue (online supplementary material Table S7).

### Oil solubility model calculations of dissolved oil exposures in test WAFs

Dissolved concentrations of individual PAHs in the undiluted recirculating WAF were predicted using the oil solubility model reported by Redman and Parkerton (2015). The two key inputs for applying this model are the oil loading, which was estimated from the amount of oil added to the generator column and the volume of water eluted through the column (ca. ∼40 g oil/310 L water = 130 mg/L), and the concentrations of PAHs in the test oil (online supplementary material Table S1). Predicted concentrations for each oil were compared with PAH measurements collected on WAF samples using both biosensor and GC-MS analysis. The predicted dissolved oil concentrations were also used to estimate summed toxic units (ƩTU) of undiluted test oil exposures to assess the potential for acute effects on test organisms during bioconcentration tests. This calculation uses the target lipid model to compute the effect concentration for each compound assuming a critical target lipid body burden of 70.8 mM, which corresponds to the median sensitivity test organism (McGrath et al., 2018).

### Derivation of oyster tissue half-lives

To determine compound-specific elimination rates (k_e_), background tissue concentrations of each analyte were determined by calculating the mean tissue concentration observed in control oysters over the test period. Observed oyster tissue concentrations in oil exposed treatments were corrected by subtracting the mean background concentration and then normalizing by the measured sample lipid content. Linear regressions were then performed using the natural logarithm of background-corrected lipid-normalized concentrations and days of depuration using JMP Pro (2021). Estimated elimination rates were derived for all significant regressions at *p* < .05 with corresponding half-lives calculated as .693/k_e_.

## Results and discussion

### Water quality and oyster survival

Acceptable water quality, including dissolved oxygen and ammonia levels, was observed throughout experiments (online supplementary material Tables S2). Temperature ranged from 12.9–17.9 °C and 15.0–17.9 °C for the Hoops and HFO tests, respectively. No mortalities were observed during the Hoops oil bioconcentration experiment or preceding acclimation period. During the HFO experiment, two mortalities occurred during the acclimation period, one control oyster and one treatment oyster; however, no mortalities were observed during the bioconcentration test. Oyster sizes were found to be significantly different between experiments (online supplementary material Figure S2). For the Hoops test, the mean oyster shell height was 91.5 ± 7.9 mm, and for the HFO test, the average shell height was 82.7 ± 6.4 mm.

### WAF exposures

Biosensor measurements of undiluted WAF for each test are provided in online supplementary material Table S3 and shown plotted in online supplementary material Figure S3 over the recirculation period. Apparent steady-state PAH concentrations were quickly achieved for Hoops and were maintained at low levels over the remainder of the test. Mean concentrations over Days 8–11 were 4.7 ± .9 μg/L. For HFO, higher concentrations were achieved with mean concentrations between 9 and 12 days of 131.6 ± 27.0 μg/L. Because biosensor measurements are expected to quantify 3–5-ring PAHs, these results can be compared with oil solubility model predictions as well as GC-MS measurements. Predicted dissolved oil concentrations for 3–5-ring PAHs in WAFs for Hoops and HFO were 1.3 and 62.2 ppb, respectively (online supplementary material Table S1). For comparison, measured concentrations based on GC-MS were 2.8 and 120.2 μg/L for Hoops and HFO, respectively (online supplementary material Table S4). The factor of two higher measured total concentrations obtained via GC-MS than the predicted dissolved concentrations suggests the presence of trace amounts of droplet oil sloughed off from the sand column in both tests. Because biosensor measurements are expected to reflect dissolved PAHs, the higher concentrations derived from biosensor measurements than solubility modeling predictions likely reflects role of 2-ring PAHs or other unmeasured, dissolved PAH compounds in the WAF that bind to the antibody and contribute to the observed response. Nevertheless, biosensor measurements of WAF effluent capture the relative differences in 3+ ring PAH exposures for the two different oils. This concordance supports the utility of biosensor measurements for characterizing 3+ ring PAH in aqueous samples consistent with results from earlier studies. Model predictions of dissolved oil exposures indicate that the ƩTUs in Hoops and HFO WAFs were .25 and 1.45 (online supplementary material Table S1), which, on dilution with York River water, is expected to reduce ƩTUs to below 1. These predictions are consistent with the lack of oyster mortality observed in bioconcentration tests.

### Aquarium exposures

**Figure 2.**
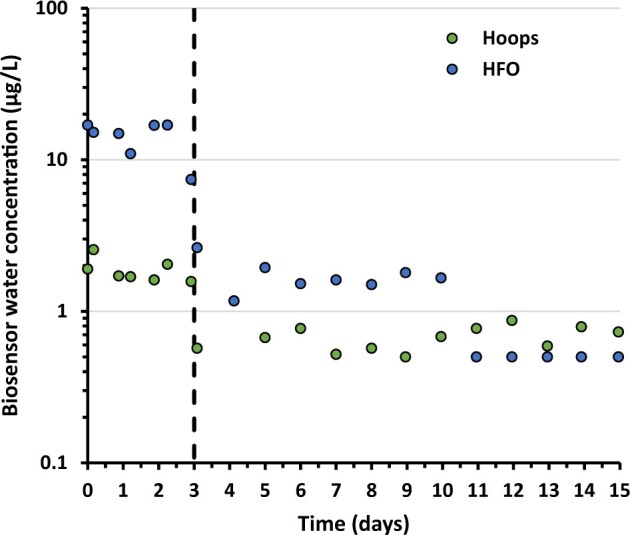
Biosensor measurements in aquaria dosed with water accommodated fraction (WAF) that was diluted with York River water during bioconcentration tests. Dashed vertical line indicates the transition from uptake (3-day period) to depuration (12-day period) phases. HFO = heavy fuel oil.

Based on biosensor results provided in online supplementary material Table S3, 3–5-ring PAH concentrations following dilution with York River water for Hoops and HFO during the uptake phase was estimated to be 1.9 ± .4 µg/L for Hoops and 15.3 ± 2.3 µg/L for HFO tests in treatment aquaria containing oysters (Figure 2). For both experiments, the water concentrations measured by the biosensor demonstrated that oysters received a consistent but order of magnitude difference in dissolved PAH exposures throughout the 3-day uptake period for the Hoops and HFO experiments. The observed aquaria concentrations were expected to decrease by a factor of two from that measured in WAFs due to dilution with York River water. Although this appears to be the case for Hoops, HFO exposures exhibited a larger, approximately 8-fold reduction in exposure concentrations. This unexpected decline may in part be due to sorption of higher molecular mass PAHs to natural particles in the river water that altered bioavailability by reducing freely dissolved concentrations and thereby limiting binding with the antibody used for biosensor quantification. Other loss processes may have contributed to the lower-than-expected biosensor results, but further investigation was beyond the study scope. On transition of aquaria from WAF effluent to clean river water flow, measured concentrations quickly dropped to low levels during the depuration phase of each bioconcentration test (Figure 2).

### Oyster accumulation and depuration using biosensor

**Figure 3.**
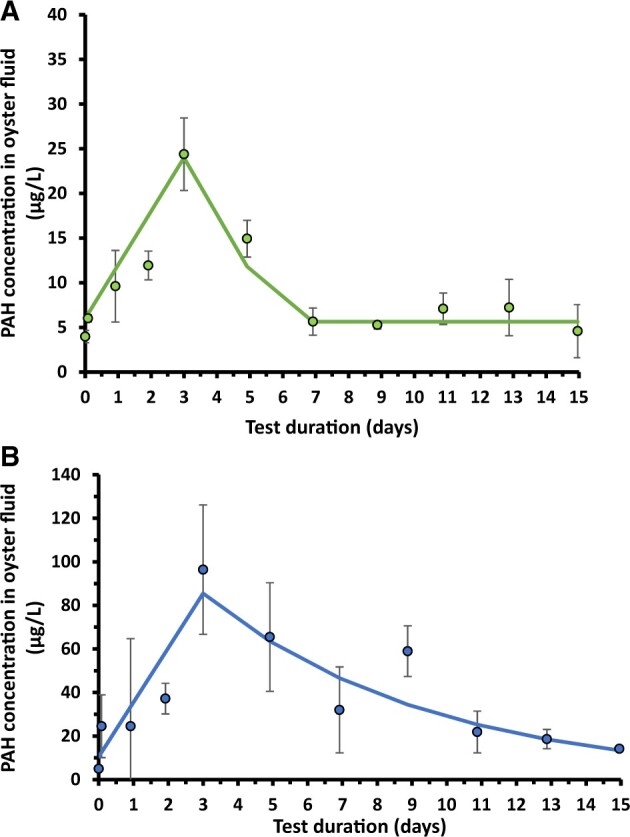
Biosensor measurements in oyster interstitial fluid following exposure to Hoops Oil (A) or heavy fuel oil (HFO) (B) over a 3-day uptake followed by a 12-day depuration phase. Error bars depict *SD* (*n* = 3) and solid line denotes one compartment, first-order model prediction. Note different y-axis scales.

All measurements of oyster interstitial fluid were well above the reported detection limit for both treatment and control oysters. The lowest oyster interstitial fluid concentration was measured in a control oyster at 1.1 µg/L. Biosensor analyses of individual oysters throughout each experiment showed a rapid uptake of PAH in the oyster interstitial fluid (Figure 3). Final concentrations on the last day of uptake were approximately 10 times higher than the estimated water concentrations to which oysters were exposed (c.f. Figures 2 and 3). On transition to the depuration phase during the lower dose Hoops oil experiment, oyster interstitial fluid concentrations quickly decreased to near the detection limit within 4 days of depuration, beyond which further elimination could not be quantified (Figure 3A). Biosensor concentrations beyond Day 7 appeared to reach an asymptotic level that matched concentrations in unexposed oysters at the start of uptake. Thus, a concentration of approximately 5 µg/L appears to reflect the background signal for this sample matrix. A predictive model was fit to the Hoops test data by assuming a linear uptake over the 3-day exposure period and a first order exponential loss over the first 4-day depuration period, resulting in an estimated uptake rate of 6.0 ± 1.0 μg/L_wet_ d^−1^ and elimination rate of .37 ± .07 d^−1^. Dividing the uptake rate by the average water concentration determined by the biosensor during the 3-day exposure period yields an estimated uptake clearance (k_u_) of 6.0/1.9 = 3.1 L_water_/L_interstital fluid_ d^−1^ and bioconcentration factor (BCF) = 3.1/.37 = 8.4 L_water_/L_interstital fluid._

Given the higher oyster interstitial fluid concentrations determined at the end of uptake following HFO exposure, decreasing concentrations were quantified over the 12-day depuration period (Figure 3B). Using observed oyster fluid concentrations, the fitted uptake and elimination rate were 25.0 ± 6.0 μg/L_interstital fluid_ d^−1^ and .12 ± .03 d^−1^, respectively. Dividing the uptake rate by the corresponding mean water concentration during HFO exposure yields a k_u_ = 1.6 L_water_/L_interstitial_ fluid d^−1^ and BCF = 1.6/.12 = 13.3 L_water_/L_interstital fluid._

The k_u_ estimates derived from both studies are within a factor of two and consistent with theory, because, for a given size organism and temperature, the uptake clearance is expected to be constant for hydrophobic dissolved substances, such as 3–5-ring PAHs, assuming no gill metabolism (Camenzuli et al., 2019). In addition, the slower estimated elimination rate and slightly higher BCF derived from the HFO test is consistent with the expected octanol-water partition coefficient (K_ow_) dependence of PAH bioconcentration and elimination as discussed in the following section.

### Comparison of PAH composition measured in oyster tissue

Compound-specific GC-MS data are summarized for Hoops Oil (Table S5) and HFO (Table S6) tests for oysters collected from both control and oil treatments. Companion lipid data for tissue samples are provided in online supplementary material Table S7. Mean percentage of lipid content on a wet mass basis for oysters in control and oil treated samples were 1.49 ± .22 and 1.70 ± .22 for the Hoops test and 1.96 ± .33 and 1.66 ± .52 for the HFO test, respectively. Lipid content between tests was not significantly different based on the Welch Two sample *t*-test (*p* = .26). Tissue analysis results revealed that the composition of PAHs accumulated in oyster tissue between the Hoops and HFO test exposures differed (Figure S4), consistent with the test oil compositions (online supplementary material Figure S1). Oysters exposed to Hoops oil accumulated predominately lower molecular mass (2 and 3 ring) PAHs. In contrast, oysters exposed to HFO exhibited a greater concentration of 4-ring PAHs and accumulated lower total concentrations of PAHs. The lower tissue concentrations measured in the HFO test reflects the reduced contribution of 2-ring PAHs and slower uptake kinetics that limited accumulation of the higher molecular weight compounds during the short 3-day uptake phase. These findings are consistent with a previous study by Lüchmann et al. (2015), who reported that oysters exposed to diesel WAFs for 4 days resulted in preferential accumulation of the more soluble 2–3-ring PAHs with lower amounts of higher 4–5-ring PAHs, despite the greater bioconcentration potential (i.e., higher K_ow_) of these chemicals.

### Comparison of biosensor-predicted vs. GC-MS-measured tissue concentrations

**Figure 4.**
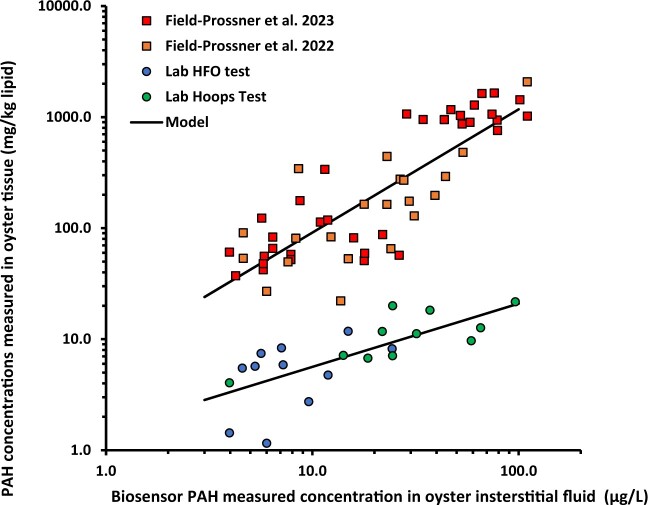
Correlation between the sum of lipid normalized concentrations of 3–5-ring polyaromatic hydrocarbons (PAHs) in oyster tissues determined using gas chromatography–mass spectrometry analysis and concentrations in oyster interstitial fluid determined using biosensor. Regression models derived using laboratory data from this study: log Y = .18 ± .15 + .57 ± .12*log X, *N* = 22, R^2^ = .53, *p* = .00013; field data from previous studies: log Y = .85 ± .14 + .70 ± .10*log X, *n* = 54, *R^2^* = .70, *p* ≤ .00001. HFO = heavy fuel oil.

Lipid normalized PAH tissue concentrations are summarized in online supplementary material Table S8. Figure 4 shows a significant positive correlation was observed between measured 3–5-ring PAH concentrations in tissue lipid (calculated as the sum of 29 parent or alkyl homologs; see online supplementary material Tables S5 and S6) and biosensor data (online supplementary material Table S3). Addition of 3-ring compounds in the dibenzothiophene and napthodibenzothiophene classes (10 additional analytes) to the 3–5-ring PAH sum resulted in a weaker correlation (online supplementary material Figure S5). Similar findings were observed when 2–5-ring PAHs (41 analytes, c.f. online supplementary material Table S5 and S6) were included in regression analysis (online supplementary material Figure S6). These results indicate a lower binding affinity for these compound classes to the biosensor antibody than the 3–5-ring PAHs.

Previous work has reported oyster biosensor and PAH tissue measurements collected in field studies (Prossner et al., 2022, 2023). In these studies, the same protocol was used to estimate 3–5-ring PAHs in oyster interstitial fluid, but a different target list of PAH analytes was quantified in oyster tissues. These data were compiled (online supplementary material Table S9) and plotted for comparison with results obtained in this study using the field 3–5-ring PAH sum metric (19 analytes) and 2–5-ring PAHs (33 analytes). Using the 3–5-ring PAH metric calculated for these field studies, a highly significant correlation is observed (Figure 4). Further, due to the low tissue concentrations of 2–5-ring PAHs in field collected oysters, the resulting regression using this alternative metric remains robust (online supplementary material Figure S6). However, although both laboratory and field regressions are significant, the resulting relationships are clearly distinct, as evidenced by the different slopes and intercepts (Figure 4).

**Figure 5.**
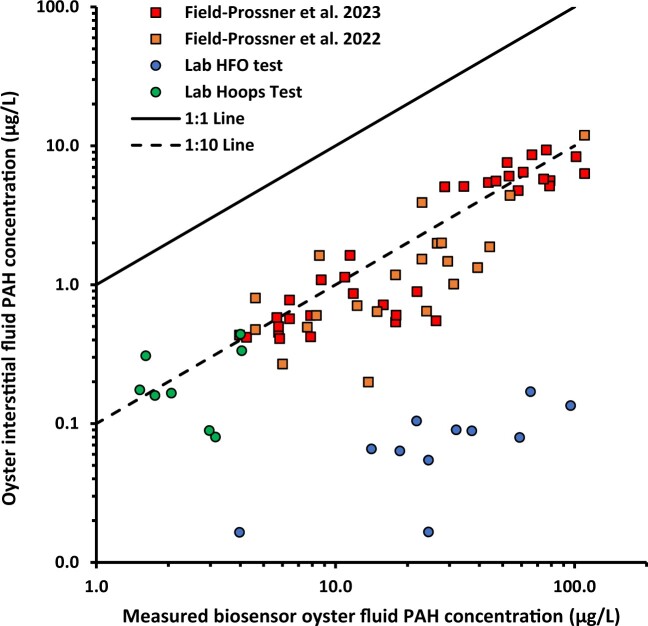
Comparison of predicted 3–5-ring polyaromatic hydrocarbon (PAH) concentrations in oyster interstitial fluid calculated assuming equilibrium partitioning with estimated concentrations determined using the biosensor. Solid line indicates 1:1 relationship and dashed lines indicate predicted concentrations at tenfold lower than biosensor estimates. HFO = heavy fuel oil.

To explore whether differences in the empirically derived laboratory and field regressions can be explained by differences in PAH composition and partitioning between lipid and interstitial fluid equilibrium, partitioning calculations were performed. To predict freely dissolved concentrations in oyster fluid, the lipid-normalized tissue concentrations of each measured PAH were divided by the corresponding K_ow_, which serves as an estimate of the lipid-water partition coefficient. Thiophene classes were excluded in this analysis due to expected insensitivity of the biosensor to heterocyclic PAHs, as previously discussed. The predicted dissolved concentrations in oyster fluid for each tissue sample from laboratory (online supplementary material Table S8) and field (online supplementary material Table S9) studies were summed to compute total dissolved PAHs. The predicted 3–5-ring and 2–5-ring PAH sum metrics were then cross-plotted against the estimated dissolved PAH concentrations obtained with the biosensor (Figure 5 and online supplementary material S7). If predicted concentrations in oyster interstitial fluid fully accounted for the observed biosensor response, the corresponding log-log plot constructed from these data should exhibit a slope of 1 and an intercept of 0. Using predicted concentrations of 3–5-ring PAHs in oyster fluid, the Hoops laboratory and field data collapse and exhibit a slope consistent with theory but an intercept that is an order of magnitude lower than expected, as denoted by the dashed line in Figure 5. However, data for the HFO laboratory study deviate and fall below this line. If predicted concentrations for 2–5-ring PAHs are instead considered, the intercept increases, but the slope deviates further from unity (online supplementary material Figure S7) suggesting inclusion of 2-ring PAHs is less reliably captured by the biosensor measurement.

We hypothesize that the discrepancy between predicted dissolved 3–5-ring PAHs in oyster fluid and biosensor measurements is due to the background signal associated with this sample matrix as well as the potential contribution of unmeasured compounds. In the case of HFO exposed oysters, compounds included in the unresolved complex mixture could explain why biosensor responses appear systematically higher than predicted interstitial fluid concentrations when compared with Hoops oil and field exposed oysters (Figure 5). Some unresolved complex mixture compounds may bind to mAb 2G8 and yield a greater estimated dissolved concentration than predicted, because only a limited number of PAHs that can be analytically resolved are included in equilibrium partitioning calculations. It is also possible that concentrations in oyster fluid are not at equilibrium with lipid given differing kinetic rates between specific organs (e.g., gill relative to hepatopancreas) from which PAHs are exchanged (Bustamante et al., 2012; Widdows et al., 1983). Another source of variability in deriving quantitative relationships for predicting tissue concentrations from biosensor measurements is differences in the PAH target analyte list as illustrated by the laboratory and field studies described in this analysis. These findings indicate that quantitative prediction of oyster tissue concentrations using biosensor measurements may vary depending on the source of PAH exposure and sum PAH metric adopted. Nevertheless, this method is informative for determining relative oyster PAH contamination for the purpose of rapid screening and prioritization for confirmatory analysis of biota samples.

### Oyster tissue half-lives

**Figure 6.**
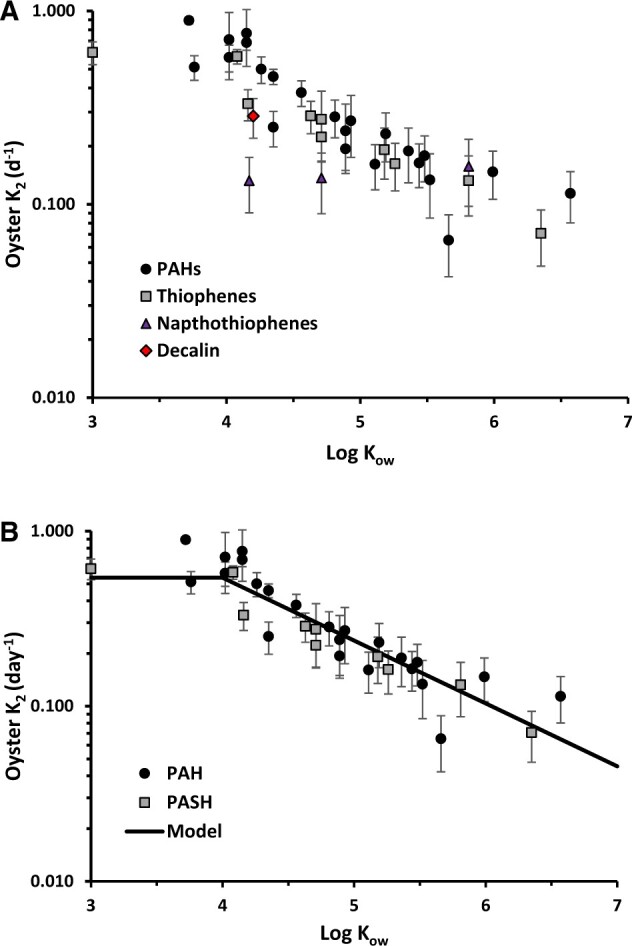
Oyster tissue elimination rates as a function of analyte log octanol-water partition coefficient (Log K_OW_). (A) all analytes with significant regressions (B) fitted model to observed data excluding decalin and napthothiophenes, PAH = polyaromatic hydrocarbons, PASH = benzothiophenes and dibenzothiophenes. A significant linear regression model was determined for Log K_ow_ above 4: k_e_ = .96 (± .19) - .314 (± .038) Log K_ow_, *R^2^* = .69, *p* < .0001.

Regressions were performed for all analytes where reported concentrations were above detection limits at the end of uptake phase as detailed in online supplementary material Appendix S1. Significant regressions for estimating elimination rates were obtained for 19 analytes in the Hoops and 16 analytes in the HFO tests, respectively (online supplementary material Table S10). Due to the different oil and resulting aqueous compositions, only four compounds (fluorene, acenaphthene, C1-phenanthenes/anthracenes, C1-dibenzothiophenes) overlapped so that elimination rates could be independently derived from both oil bioconcentration tests. For these compounds the ratio of k_e_ derived from Hoops to HFO tests ranged from .81 to .9 and based on the reported standard errors about the estimates, were not statistically different (online supplementary material Table S10). These results indicate that compound-specific elimination rates are consistent between PAH mixture exposures generated using different test oils. As shown in Figure 6A, half-lives calculated from elimination rates (online supplementary material Table S10) spanned from .8 to 10.6 days over a more than three order of magnitude range in Log K_ow_ (3.0–6.6). For comparison, the half-life of PAHs in oyster fluid determined using biosensor measurements during the depuration period of for Hoops Oil and HFO tests were 1.9 and 5.8 d, respectively. These results suggest depuration of PAHs in oyster interstitial fluid shows comparable rates to individual PAHs in tissues. Elimination rates for decalin and lower Log K_ow_ naphthothiophene homologs appeared lower than other PAHs (Figure 6A). Further, elimination rates have not been reported for these compounds in earlier literature studies. Therefore, these compounds were excluded in the development of a predictive model for estimating PAH oyster elimination rates from Log K_ow_ using our study data, as shown in Figure 6B.

**Figure 7.**
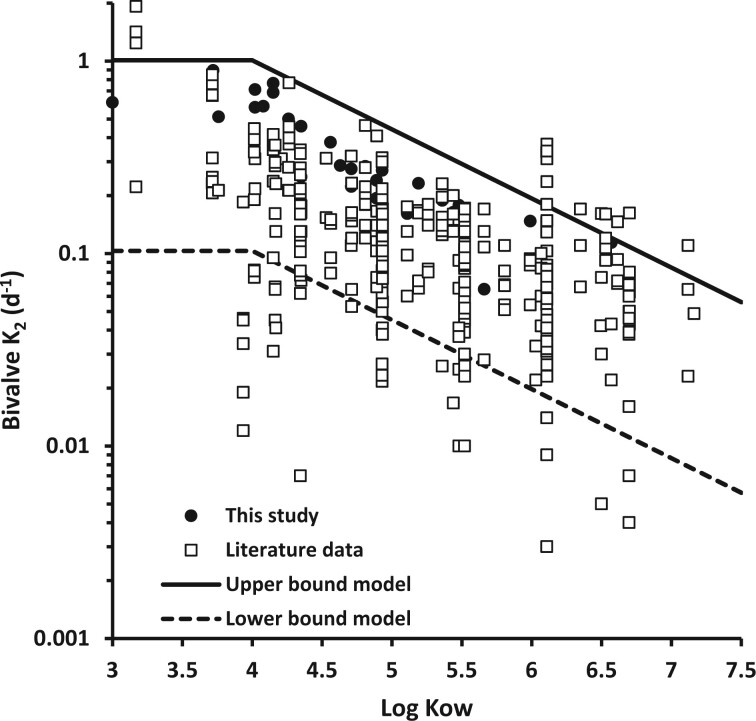
Bivalve tissue elimination rates for polyaromatic hydrocarbons (PAHs) obtained in this study (filled circles) and reported in literature (open squares) as a function of the log octanol-water partition coefficient. Between log octanol-water partition coefficient (Log K_ow_) from 3 to 4, a lower and upper bound model elimination rate of .1 to 1 d^−1^ is assumed. For PAHs with log K_ow_ > 4, the elimination rate is assumed to decline linearly with the same slope as the model shown in Figure 6.

Elimination rates for PAHs from earlier studies are compiled in online supplementary material Table S11 for various marine and freshwater bivalves, including various species of oysters, clams, and mussels (Bender et al., 1988; Enwere et al., 2009; Farrington et al., 1982; Gewurtz et al., 2002; Huckins et al., 2004; Idowu et al., 2020; McLeese & Burridge, 1987; Pruell et al., 1986; Richardson et al., 2005; Séguin et al., 2022; Sericano et al., 1996; Thorsen et al., 2004; Yakan et al., 2011, 2013). A detailed comparison of individual studies to our results is presented in online supplementary material Figure S8, and a summary of all literature data is shown in Figure 7. The observed range of elimination rates at a given Log K_ow_ typically varies over an order of magnitude and likely reflects differences in size-dependent filtration rates, lipid content, growth rates, temperature, and species-specific variation in PAH biotransformation capability. A number of studies have demonstrated that bivalves can metabolize PAHs (dos Reis et al., 2020; Lüchmann et al., 2015; Zhou et al., 2022). The low elimination rates that appear as outliers at Log K_ow_ < 4.5 may be due to possible analytical errors for these higher volatility compounds. Nevertheless, the proposed upper and lower bound model captures all elimination rates obtained in this study and approximately 84% of the compiled bivalve elimination rate data. Therefore, the model detailed in Figure 7 provides a convenient tool to predict the expected range in bivalve tissue half-life for specific PAHs.

## Summary and conclusion

Laboratory studies were performed with *C. virginica* oysters acutely exposed to WAFs prepared with two different test oils to simulate transient oil spill exposures. Our objective was to evaluate antibody-based biosensor technology as a screening tool to measure total 3–5-ring PAH concentrations in water and oyster interstitial fluid (i.e., aqueous phase) during uptake and depuration phases of these bioconcentration tests. We then examined correlations between lipid-normalized oyster tissue concentrations determined using traditional GC-MS analysis and rapid biosensor measurements of oyster interstitial fluid derived in this laboratory and previous field studies. Finally, we derived PAH specific oyster tissue elimination rates from data generated in this study and compared our results with elimination rate data reported for bivalves in the literature.

Our findings indicate that biosensor measurements were able to quantify relative differences in 3–5-ring PAH aqueous exposures in bioconcentration tests using the two different composition test oils. Biosensor measurements of oyster interstitial fluid also captured the kinetics of oyster PAH accumulation during uptake and depuration phases. However, quantitative prediction of lipid-normalized 3–5-ring PAH oyster tissue concentrations differed between laboratory and field studies, indicating that the biosensor is best used as a rapid screening tool to characterize relative tissue concentrations following exposure to a common PAH source. Thus, the low-cost, near real-time biosensor technology reported in this study and earlier work appears to offer a promising analytical tool that warrants further consideration in the context of oil spill monitoring and shellfisheries management. A generic model has also been developed from study and literature data to predict how quickly specific PAHs will be lost from bivalves after oil exposures are terminated. These predictions can help inform field monitoring of shellfish following an oil spill and estimate recovery times required to achieve pre-spill tissue concentrations.

## Supplementary Material

vgae024_Supplementary_Data

## Data Availability

All data are provided in the supplemental information and/or will be made available on request.
