# Supplementary material for: Rapid screening of shellfish tainting from oil spills using an antibody-based biosensor
Source: Environ Toxicol Chem. 2025 Jan 6;44(1):270–81. doi: 10.1093/etojnl/vgae024 (PMC11790208; doi:10.1093/etojnl/vgae024)
Supplement: vgae024_Supplementary_Data [file vgae024_supplementary_data.zip › vgae024_Supplementary_Data/Appendix I Oyster Tissue Depuration Regression Analysis.pdf]

Bivariate Fit of LnConc By day Oil=HCO, PAH=ACENAPHTHENE

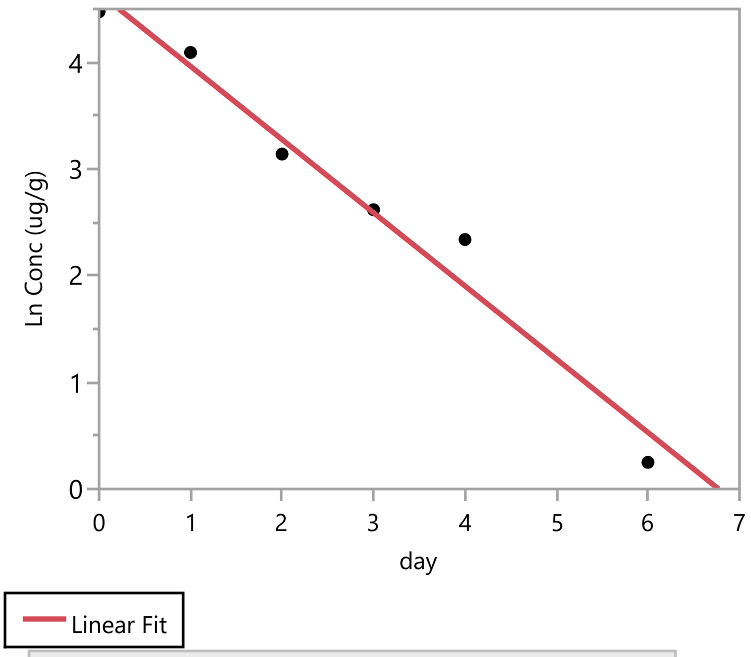

Linear Fit

LnConc = 4.6499024 - 0.6866065*day

Summary of Fit

RSquare	0.970464
RSquare Adj	0.963081
Root Mean Square Error	0.2893
Mean of Response	2.818952
Observations (or Sum Wgts)	6

Analysis of Variance

Source	DF	Sum of Squares	Mean Square	F Ratio
Model	1	10.999998	11.0000	131.4302
Error	4	0.334779	0.0837	**Prob > F**
C. Total	5	11.334777		0.0003*

Parameter Estimates

Term	Estimate	Std Error	t Ratio	Prob>|t|
Intercept	4.6499024	0.198635	23.41	<.0001*
day	-0.686607	0.059891	-11.46	0.0003*

Bivariate Fit of LnConc By day Oil=HCO, PAH=ACENAPHTHYLENE

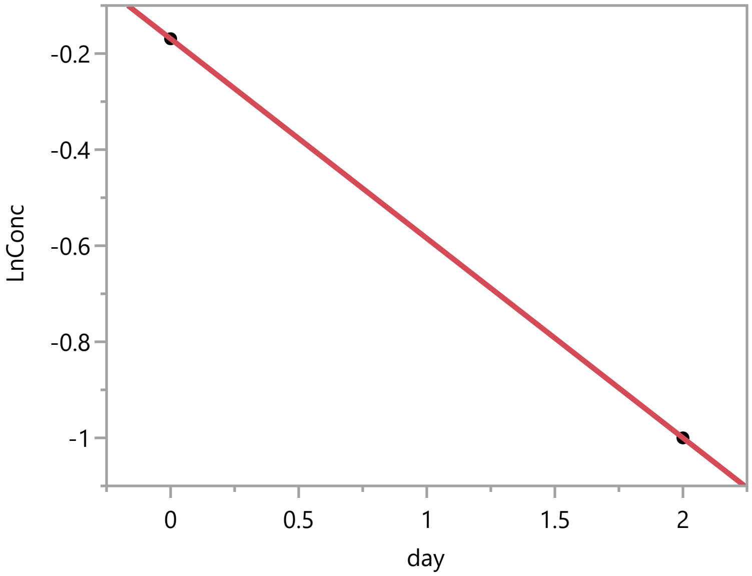

Linear Fit

LnConc = -0.169192 - 0.4154196*day

Summary of Fit

RSquare	1
RSquare Adj	.
Root Mean Square Error	.
Mean of Response	-0.58461
Observations (or Sum Wgts)	2

Analysis of Variance

Source	DF	Sum of Squares	Mean Square	MSE used	F Ratio
Model	1	0.34514688	0.345147	.	.
Error	0	0.00000000	.	**DFE used**	**Prob > F**
C. Total	1	0.34514688		.	.

Parameter Estimates

Term	Estimate	Std Error	t Ratio	Prob>|t|
Intercept	-0.169192	.	.	.
day	-0.41542	.	.	.

Bivariate Fit of LnConc By day Oil=HCO, PAH=ANTHRACENE

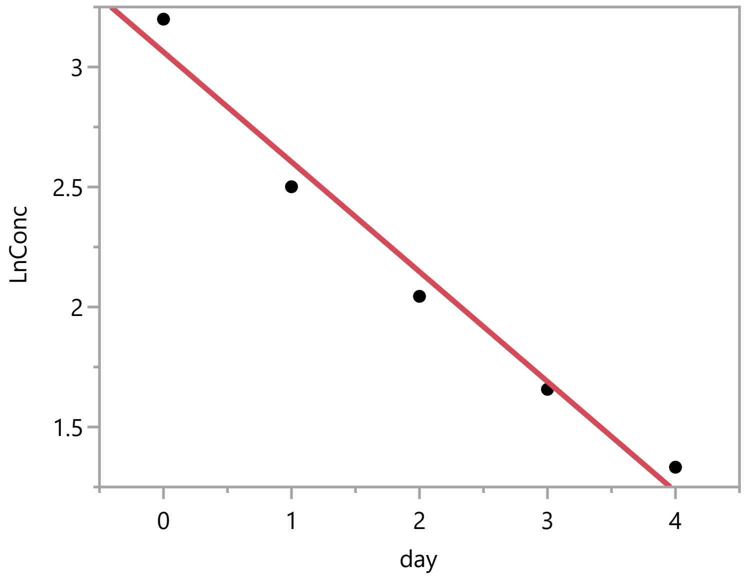

Linear Fit

LnConc = 3.0624249 - 0.4577828*day

Summary of Fit

RSquare	0.97615
RSquare Adj	0.9682
Root Mean Square Error	0.130642
Mean of Response	2.146859
Observations (or Sum Wgts)	5

Analysis of Variance

Source	DF	Sum of Squares	Mean Square	F Ratio
Model	1	2.0956510	2.09565	122.7872
Error	3	0.0512020	0.01707	**Prob > F**
C. Total	4	2.1468531		0.0016*

Parameter Estimates

Term	Estimate	Std Error	t Ratio	Prob>|t|
Intercept	3.0624249	0.101195	30.26	<.0001*
day	-0.457783	0.041313	-11.08	0.0016*

Bivariate Fit of LnConc By day Oil=HCO, PAH=BENZ(A)ANTHRACENE

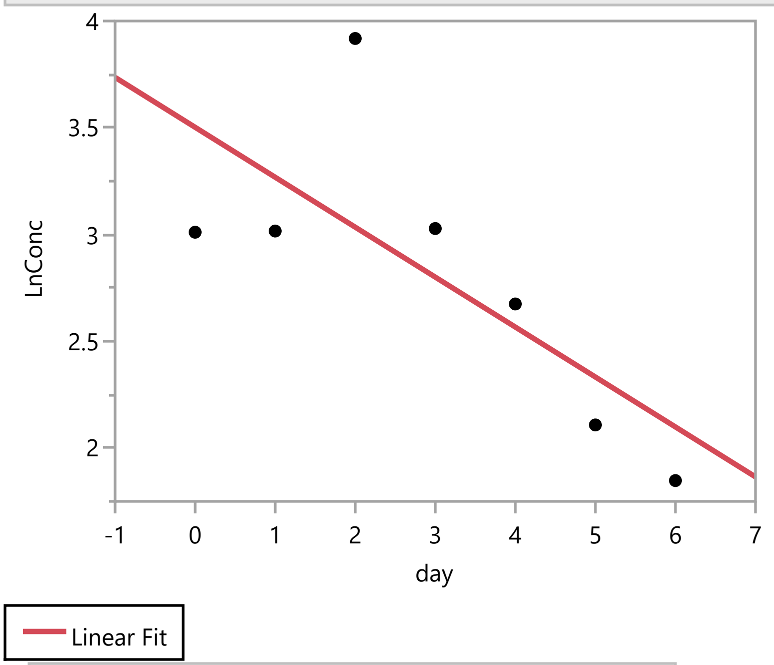

Linear Fit

LnConc = 3.5022178 - 0.233923*day

Summary of Fit

RSquare	0.548089
RSquare Adj	0.457707
Root Mean Square Error	0.502653
Mean of Response	2.800449
Observations (or Sum Wgts)	7

Analysis of Variance

Source	DF	Sum of Squares	Mean Square	F Ratio
Model	1	1.5321596	1.53216	6.0641
Error	5	1.2632980	0.25266	**Prob > F**
C. Total	6	2.7954576		0.0571

Parameter Estimates

Term	Estimate	Std Error	t Ratio	Prob>|t|
Intercept	3.5022178	0.3425	10.23	0.0002*
day	-0.233923	0.094992	-2.46	0.0571

Bivariate Fit of LnConc By day Oil=HCO, PAH=BENZO(A)PYRENE

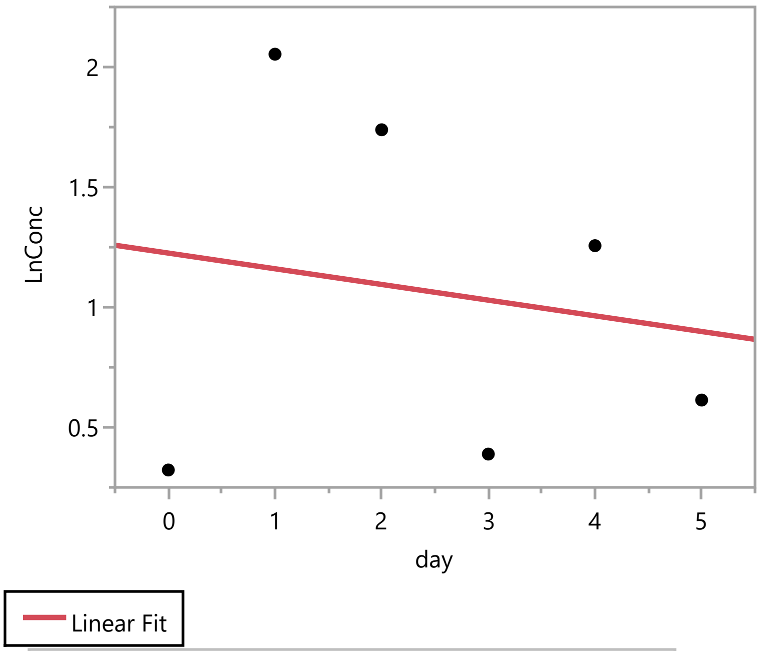

Linear Fit

LnConc = 1.2254711 - 0.0653142*day

Summary of Fit

RSquare	0.027848
RSquare Adj	-0.21519
Root Mean Square Error	0.807173
Mean of Response	1.062186
Observations (or Sum Wgts)	6

Analysis of Variance

Source	DF	Sum of Squares	Mean Square	F Ratio
Model	1	0.0746540	0.074654	0.1146
Error	4	2.6061154	0.651529	**Prob > F**
C. Total	5	2.6807695		0.7520

Parameter Estimates

Term	Estimate	Std Error	t Ratio	Prob>|t|
Intercept	1.2254711	0.584189	2.10	0.1039
day	-0.065314	0.192951	-0.34	0.7520

Bivariate Fit of LnConc By day
Oil=HCO, PAH=BENZO(B)FLUORANTHENE

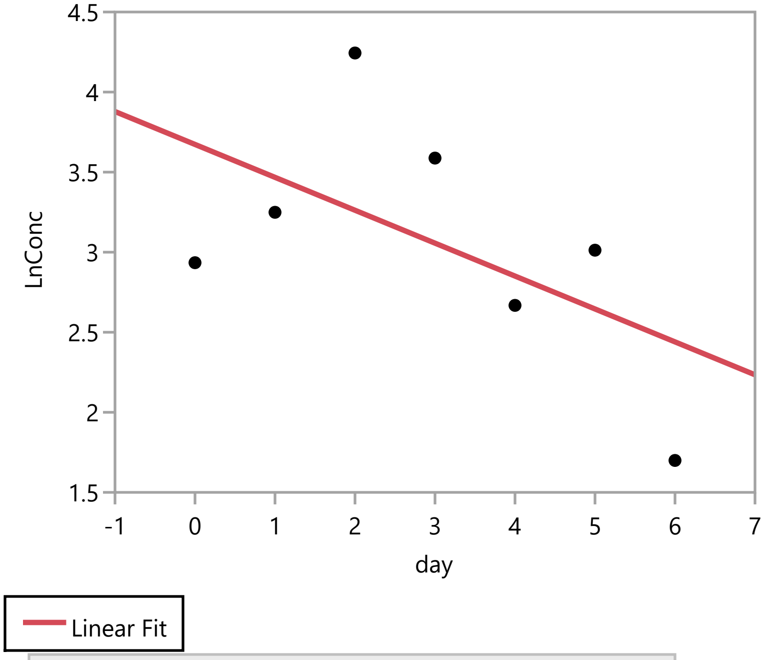

Linear Fit

LnConc = 3.672965 - 0.2055176*day

Summary of Fit

RSquare	0.316298
RSquare Adj	0.179558
Root Mean Square Error	0.715036
Mean of Response	3.056412
Observations (or Sum Wgts)	7

Analysis of Variance

Source	DF	Sum of Squares	Mean Square	F Ratio
Model	1	1.1826493	1.18265	2.3131
Error	5	2.5563855	0.51128	**Prob > F**
C. Total	6	3.7390348		0.1888

Parameter Estimates

Term	Estimate	Std Error	t Ratio	Prob>|t|
Intercept	3.672965	0.487215	7.54	0.0007*
day	-0.205518	0.135129	-1.52	0.1888

Bivariate Fit of LnConc By day Oil=HCO, PAH=BENZO(B)FLUORENE

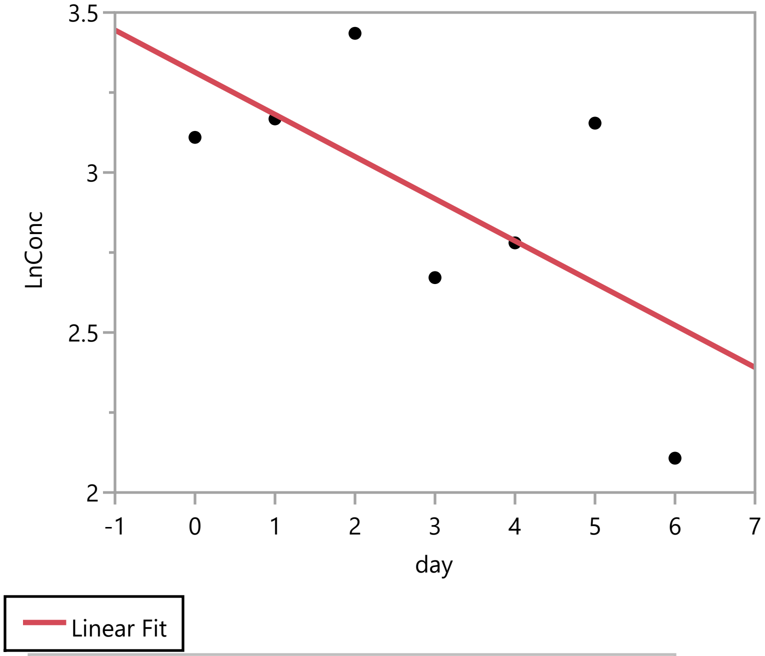

Linear Fit

LnConc = 3.3131254 - 0.1317479*day

Summary of Fit

RSquare	0.419289
RSquare Adj	0.303146
Root Mean Square Error	0.366911
Mean of Response	2.917882
Observations (or Sum Wgts)	7

Analysis of Variance

Source	DF	Sum of Squares	Mean Square	F Ratio
Model	1	0.4860100	0.486010	3.6101
Error	5	0.6731200	0.134624	**Prob > F**
C. Total	6	1.1591299		0.1159

Parameter Estimates

Term	Estimate	Std Error	t Ratio	Prob>|t|
Intercept	3.3131254	0.250008	13.25	<.0001*
day	-0.131748	0.06934	-1.90	0.1159

Bivariate Fit of LnConc By day Oil=HCO, PAH=BENZO(E)PYRENE

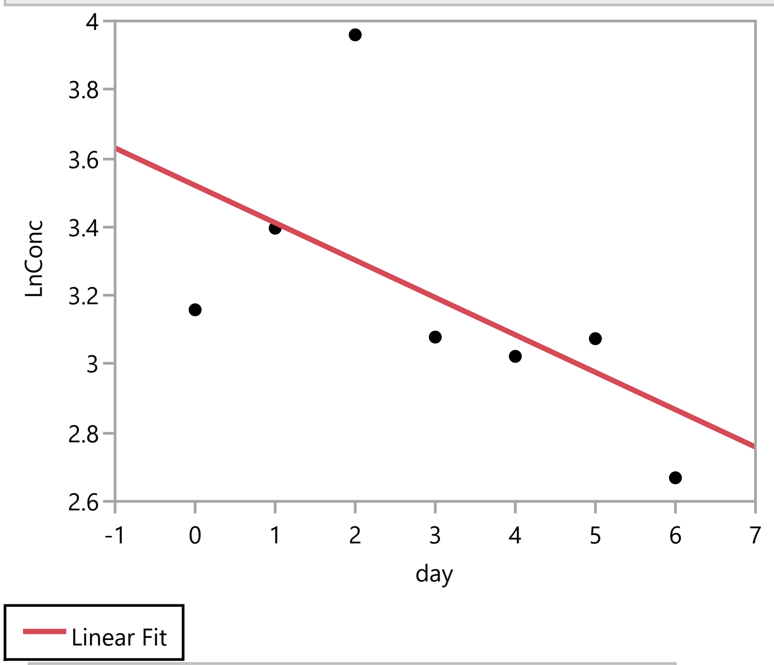

Linear Fit

LnConc = 3.5208569 - 0.1089205*day

Summary of Fit

RSquare	0.345566
RSquare Adj	0.21468
Root Mean Square Error	0.354708
Mean of Response	3.194095
Observations (or Sum Wgts)	7

Analysis of Variance

Source	DF	Sum of Squares	Mean Square	F Ratio
Model	1	0.33218292	0.332183	2.6402
Error	5	0.62908772	0.125818	Prob > F
C. Total	6	0.96127064		0.1651

Parameter Estimates

Term	Estimate	Std Error	t Ratio	Prob>|t|
Intercept	3.5208569	0.241693	14.57	<.0001*
day	-0.108921	0.067033	-1.62	0.1651

Bivariate Fit of LnConc By day
Oil=HCO, PAH=BENZO(J)+(K)FLUORANTHENE

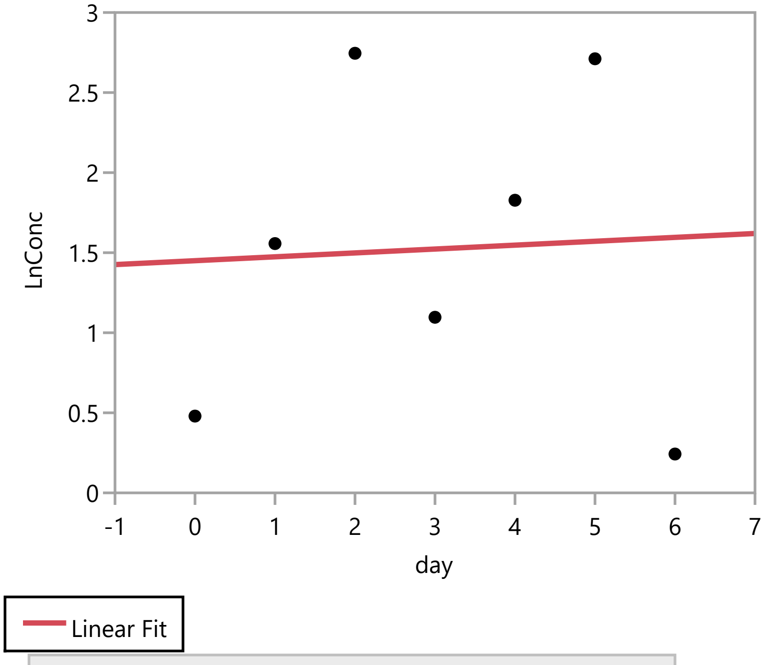

Linear Fit

LnConc = 1.4499933 + 0.0242702*day

Summary of Fit

RSquare	0.002791
RSquare Adj	-0.19665
Root Mean Square Error	1.085659
Mean of Response	1.522804
Observations (or Sum Wgts)	7

Analysis of Variance

Source	DF	Sum of Squares	Mean Square	F Ratio
Model	1	0.0164933	0.01649	0.0140
Error	5	5.8932762	1.17866	**Prob > F**
C. Total	6	5.9097694		0.9104

Parameter Estimates

Term	Estimate	Std Error	t Ratio	Prob>|t|
Intercept	1.4499933	0.739752	1.96	0.1073
day	0.0242702	0.20517	0.12	0.9104

Bivariate Fit of LnConc By day Oil=HCO, PAH=BENZOTHIOPHENE

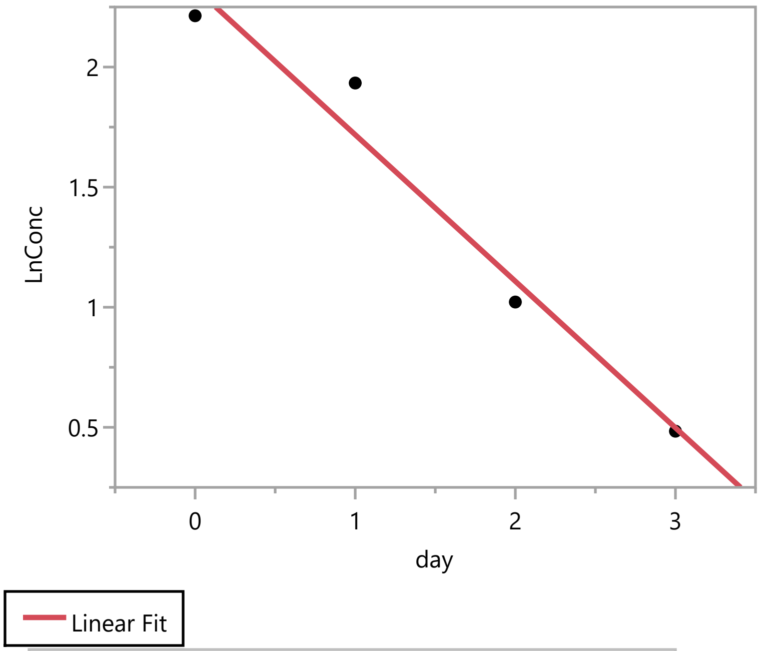

Linear Fit

LnConc = 2.3282751 - 0.610079*day

Summary of Fit

RSquare	0.965157
RSquare Adj	0.947735
Root Mean Square Error	0.183281
Mean of Response	1.413157
Observations (or Sum Wgts)	4

Analysis of Variance

Source	DF	Sum of Squares	Mean Square	F Ratio
Model	1	1.8609818	1.86098	55.3999
Error	2	0.0671836	0.03359	**Prob > F**
C. Total	3	1.9281654		0.0176*

Parameter Estimates

Term	Estimate	Std Error	t Ratio	Prob>|t|
Intercept	2.3282751	0.153344	15.18	0.0043*
day	-0.610079	0.081966	-7.44	0.0176*

Bivariate Fit of LnConc By day Oil=HCO, PAH=BIPHENYL

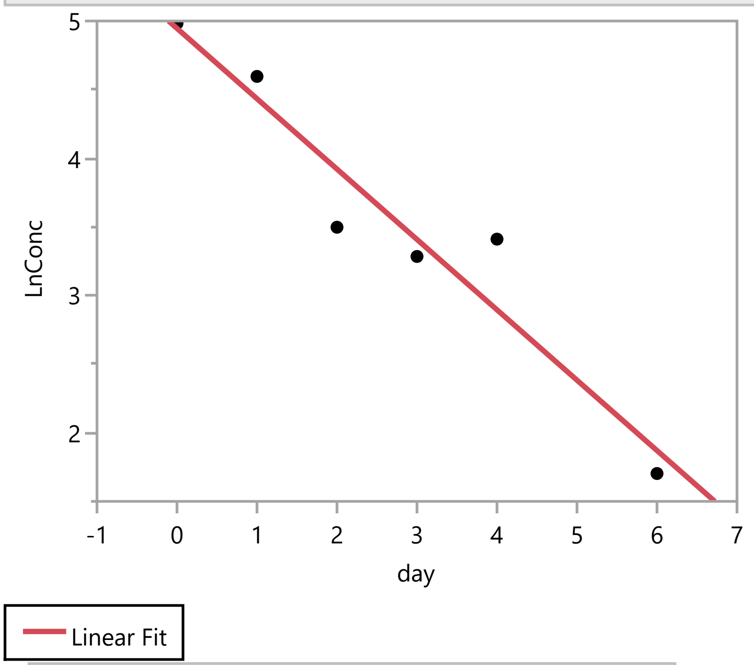

Linear Fit

LnConc = 4.9463253 - 0.512662*day

Summary of Fit

RSquare	0.922419
RSquare Adj	0.903024
Root Mean Square Error	0.359089
Mean of Response	3.579227
Observations (or Sum Wgts)	6

Analysis of Variance

Source	DF	Sum of Squares	Mean Square	F Ratio
Model	1	6.1325213	6.13252	47.5591
Error	4	0.5157809	0.12895	**Prob > F**
C. Total	5	6.6483022		0.0023*

Parameter Estimates

Term	Estimate	Std Error	t Ratio	Prob>|t|
Intercept	4.9463253	0.246553	20.06	<.0001*
day	-0.512662	0.074339	-6.90	0.0023*

Bivariate Fit of LnConc By day Oil=HCO, PAH=C1-BENZO(B)THIOPHENES

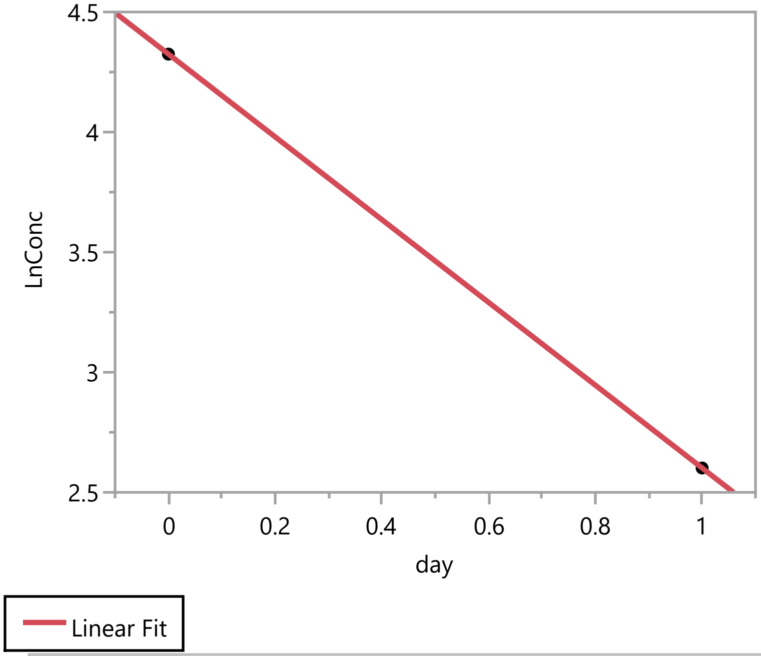

Linear Fit

LnConc = 4.3247062 - 1.7232584*day

Summary of Fit

RSquare	1
RSquare Adj	.
Root Mean Square Error	.
Mean of Response	3.463077
Observations (or Sum Wgts)	2

Analysis of Variance

Source	DF	Sum of Squares	Mean Square	MSE used	F Ratio
Model	1	1.4848098	1.48481	.	.
Error	0	0.0000000	.	**DFE used**	**Prob > F**
C. Total	1	1.4848098		.	.

Parameter Estimates

Term	Estimate	Std Error	t Ratio	Prob>|t|
Intercept	4.3247062	.	.	.
day	-1.723258	.	.	.

Bivariate Fit of LnConc By day Oil=HCO, PAH=C1-CHRYSENES

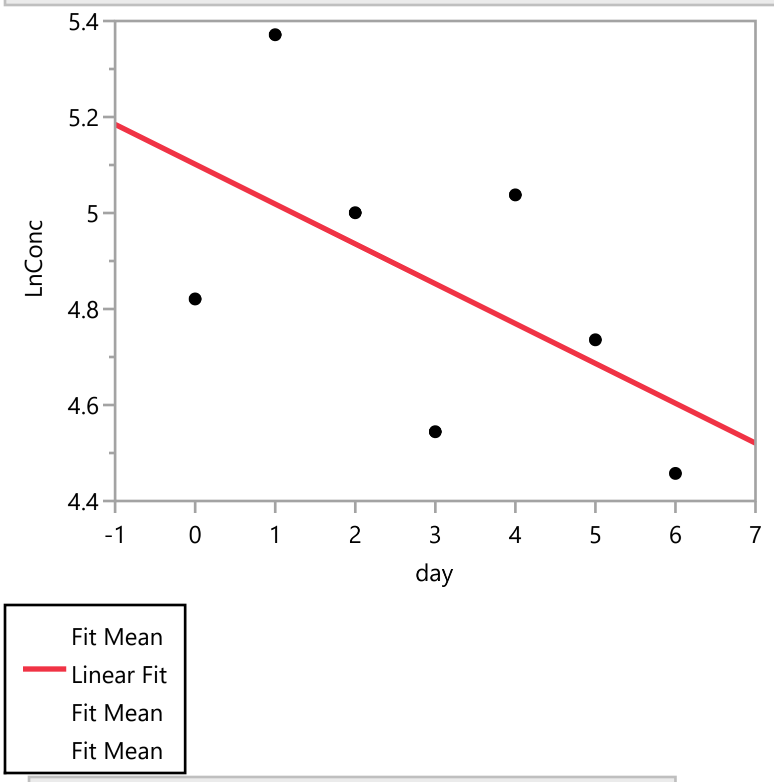

Linear Fit

LnConc = 5.1016346 - 0.0830088*day

Summary of Fit

RSquare	0.3264
RSquare Adj	0.19168
Root Mean Square Error	0.282192
Mean of Response	4.852608
Observations (or Sum Wgts)	7

Analysis of Variance

Source	DF	Sum of Squares	Mean Square	F Ratio
Model	1	0.19293289	0.192933	2.4228
Error	5	0.39816055	0.079632	**Prob > F**
C. Total	6	0.59109344		0.1803

Parameter Estimates

Term	Estimate	Std Error	t Ratio	Prob>|t|
Intercept	5.1016346	0.192281	26.53	<.0001*
day	-0.083009	0.053329	-1.56	0.1803

Bivariate Fit of LnConc By day Oil=HCO, PAH=C1-DECALINS

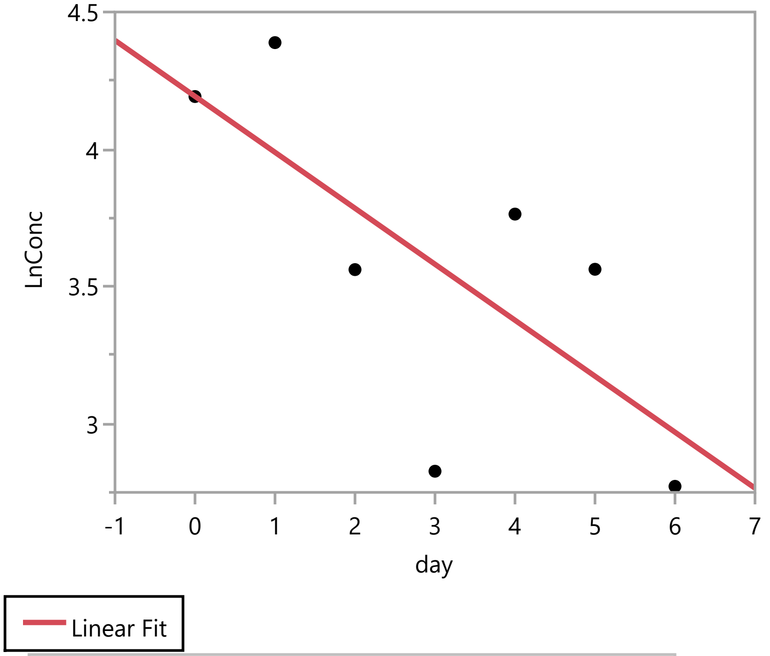

Linear Fit

LnConc = 4.192839 - 0.2039175*day

Summary of Fit

RSquare	0.510084
RSquare Adj	0.412101
Root Mean Square Error	0.472921
Mean of Response	3.581087
Observations (or Sum Wgts)	7

Analysis of Variance

Source	DF	Sum of Squares	Mean Square	F Ratio
Model	1	1.1643056	1.16431	5.2058
Error	5	1.1182709	0.22365	**Prob > F**
C. Total	6	2.2825764		0.0714

Parameter Estimates

Term	Estimate	Std Error	t Ratio	Prob>|t|
Intercept	4.192839	0.322241	13.01	<.0001*
day	-0.203917	0.089374	-2.28	0.0714

Bivariate Fit of LnConc By day Oil=HCO, PAH=C1-DIBENZOTHIOPHENES

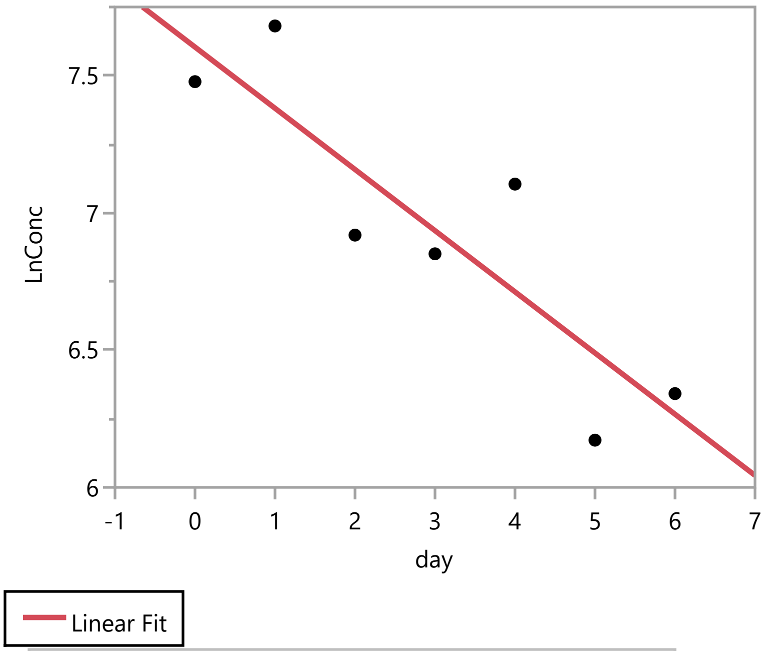

Linear Fit

LnConc = 7.6040593 - 0.2229119*day

Summary of Fit

RSquare	0.76367
RSquare Adj	0.716403
Root Mean Square Error	0.29345
Mean of Response	6.935324
Observations (or Sum Wgts)	7

Analysis of Variance

Source	DF	Sum of Squares	Mean Square	F Ratio
Model	1	1.3913121	1.39131	16.1568
Error	5	0.4305651	0.08611	Prob > F
C. Total	6	1.8218772		0.0101*

Parameter Estimates

Term	Estimate	Std Error	t Ratio	Prob>|t|
Intercept	7.6040593	0.199953	38.03	<.0001*
day	-0.222912	0.055457	-4.02	0.0101*

Bivariate Fit of LnConc By day
Oil=HCO, PAH=C1-FLUORANTHENES/PYRENES

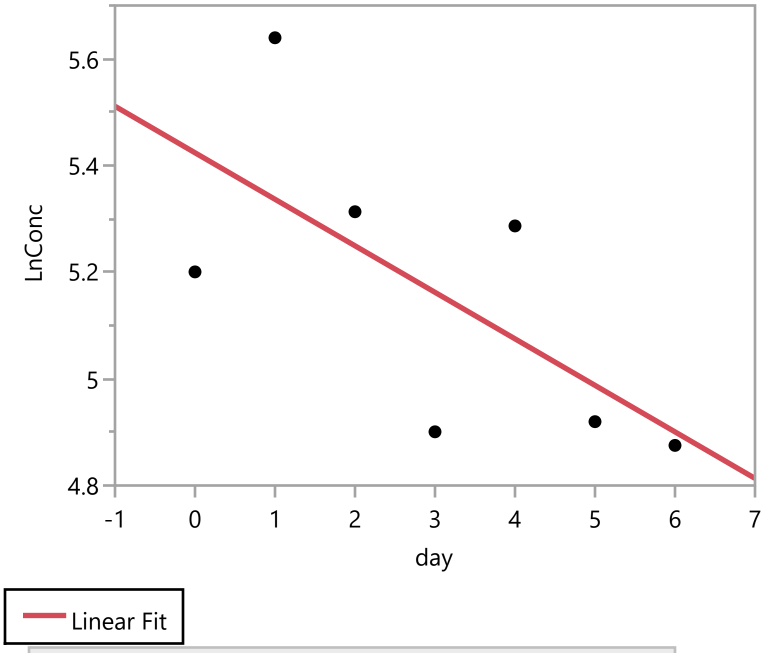

Linear Fit

LnConc = 5.4238865 - 0.0872212*day

Summary of Fit

RSquare	0.446383
RSquare Adj	0.33566
Root Mean Square Error	0.229862
Mean of Response	5.162223
Observations (or Sum Wgts)	7

Analysis of Variance

Source	DF	Sum of Squares	Mean Square	F Ratio
Model	1	0.21301085	0.213011	4.0315
Error	5	0.26418167	0.052836	**Prob > F**
C. Total	6	0.47719252		0.1009

Parameter Estimates

Term	Estimate	Std Error	t Ratio	Prob>|t|
Intercept	5.4238865	0.156624	34.63	<.0001*
day	-0.087221	0.04344	-2.01	0.1009

Bivariate Fit of LnConc By day Oil=HCO, PAH=C1-FLUORENES

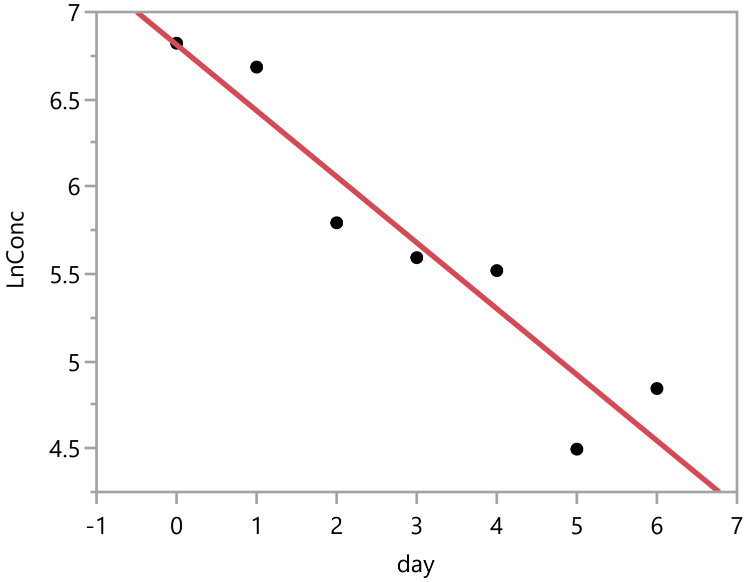

Linear Fit

LnConc = 6.8124077 - 0.3780857*day

Summary of Fit

RSquare	0.897191
RSquare Adj	0.87663
Root Mean Square Error	0.30287
Mean of Response	5.678151
Observations (or Sum Wgts)	7

Analysis of Variance

Source	DF	Sum of Squares	Mean Square	F Ratio
Model	1	4.0025664	4.00257	43.6341
Error	5	0.4586515	0.09173	**Prob > F**
C. Total	6	4.4612179		0.0012*

Parameter Estimates

Term	Estimate	Std Error	t Ratio	Prob>|t|
Intercept	6.8124077	0.206371	33.01	<.0001*
day	-0.378086	0.057237	-6.61	0.0012*

Bivariate Fit of LnConc By day Oil=HCO, PAH=C1-NAPHTHALENES

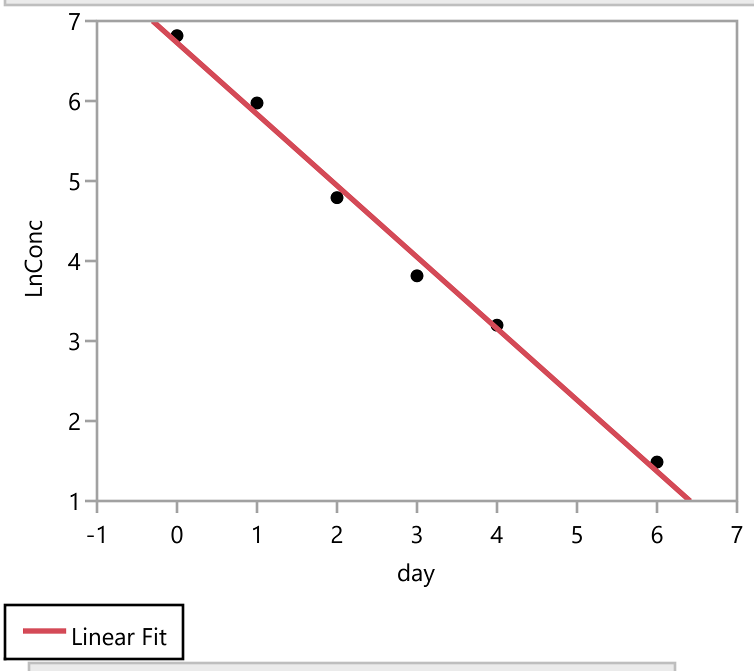

Linear Fit

LnConc = 6.7285332 - 0.8931123*day

Summary of Fit

RSquare	0.993586
RSquare Adj	0.991983
Root Mean Square Error	0.173308
Mean of Response	4.3469
Observations (or Sum Wgts)	6

Analysis of Variance

Source	DF	Sum of Squares	Mean Square	F Ratio
Model	1	18.611822	18.6118	619.6572
Error	4	0.120143	0.0300	**Prob > F**
C. Total	5	18.731965		<.0001*

Parameter Estimates

Term	Estimate	Std Error	t Ratio	Prob>|t|
Intercept	6.7285332	0.118994	56.54	<.0001*
day	-0.893112	0.035878	-24.89	<.0001*

Bivariate Fit of LnConc By day
Oil=HCO, PAH=C1-NAPHTHOBENZOTHIOPHENES

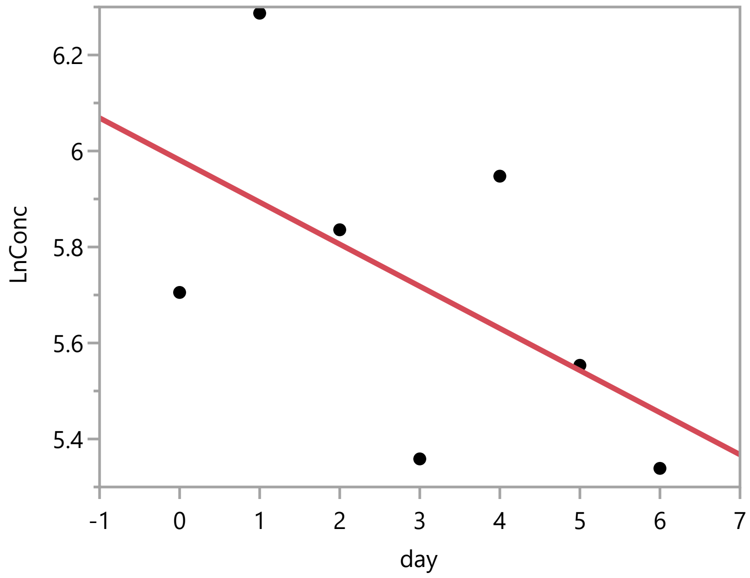

Linear Fit

LnConc = 5.9811413 - 0.0876796*day

Summary of Fit

RSquare	0.311649
RSquare Adj	0.173979
Root Mean Square Error	0.308364
Mean of Response	5.718103
Observations (or Sum Wgts)	7

Analysis of Variance

Source	DF	Sum of Squares	Mean Square	F Ratio
Model	1	0.21525586	0.215256	2.2637
Error	5	0.47544311	0.095089	**Prob > F**
C. Total	6	0.69069897		0.1928

Parameter Estimates

Term	Estimate	Std Error	t Ratio	Prob>|t|
Intercept	5.9811413	0.210115	28.47	<.0001*
day	-0.08768	0.058275	-1.50	0.1928

Bivariate Fit of LnConc By day Oil=HCO, PAH=C1-PHENANTHRENES/ANTHRACENES

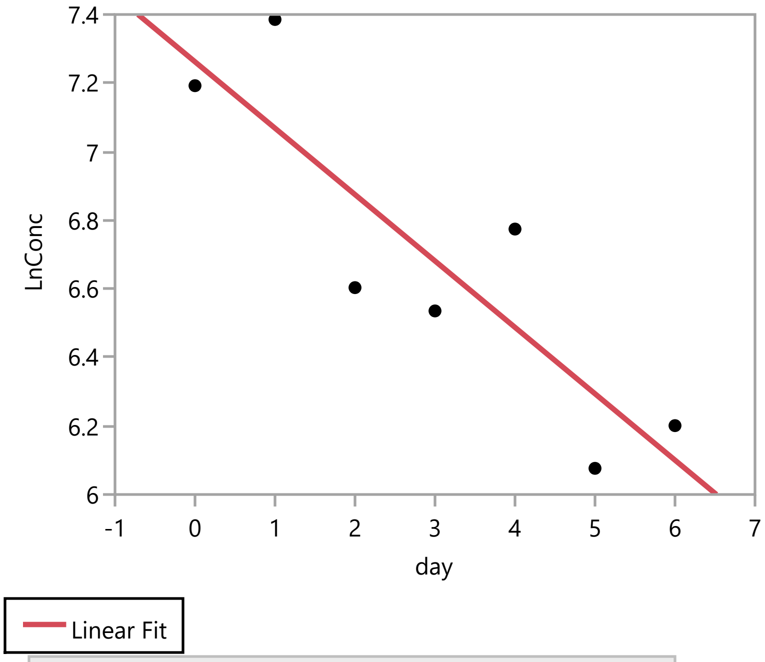

Linear Fit

LnConc = 7.2620716 - 0.1936429*day

Summary of Fit

RSquare	0.755425
RSquare Adj	0.706511
Root Mean Square Error	0.260739
Mean of Response	6.681143
Observations (or Sum Wgts)	7

Analysis of Variance

Source	DF	Sum of Squares	Mean Square	F Ratio
Model	1	1.0499325	1.04993	15.4437
Error	5	0.3399233	0.06798	**Prob > F**
C. Total	6	1.3898558		0.0111*

Parameter Estimates

Term	Estimate	Std Error	t Ratio	Prob>|t|
Intercept	7.2620716	0.177663	40.88	<.0001*
day	-0.193643	0.049275	-3.93	0.0111*

Bivariate Fit of LnConc By day
Oil=HCO, PAH=C2-BENZO(B)THIOPHENES

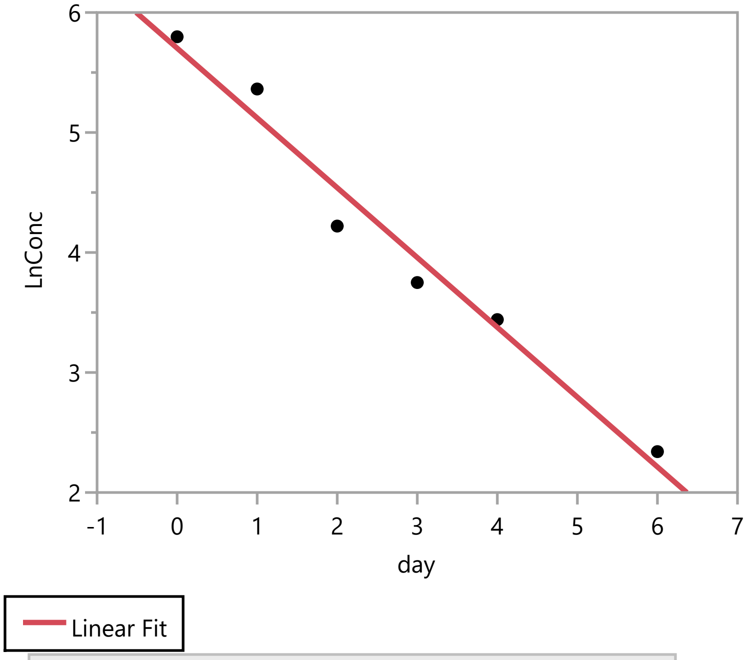

Linear Fit

LnConc = 5.7029583 - 0.5816366*day

Summary of Fit

RSquare	0.971316
RSquare Adj	0.964145
Root Mean Square Error	0.241407
Mean of Response	4.151927
Observations (or Sum Wgts)	6

Analysis of Variance

Source	DF	Sum of Squares	Mean Square	F Ratio
Model	1	7.8936924	7.89369	135.4506
Error	4	0.2331091	0.05828	**Prob > F**
C. Total	5	8.1268015		0.0003*

Parameter Estimates

Term	Estimate	Std Error	t Ratio	Prob>|t|
Intercept	5.7029583	0.165752	34.41	<.0001*
day	-0.581637	0.049976	-11.64	0.0003*

Bivariate Fit of LnConc By day Oil=HCO, PAH=C2-CHRYSENES

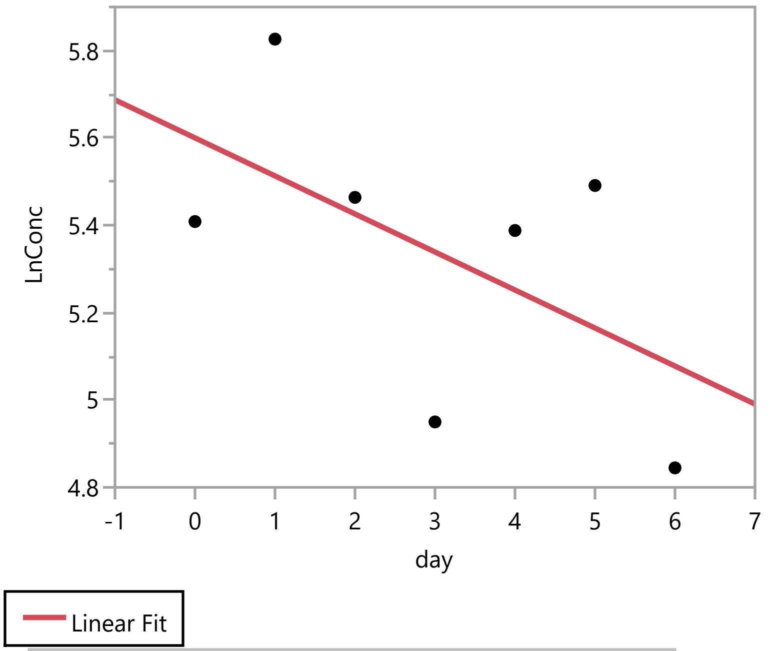

Linear Fit

LnConc = 5.6002733 - 0.0870834*day

Summary of Fit

RSquare	0.312472
RSquare Adj	0.174966
Root Mean Square Error	0.305682
Mean of Response	5.339023
Observations (or Sum Wgts)	7

Analysis of Variance

Source	DF	Sum of Squares	Mean Square	F Ratio
Model	1	0.21233857	0.212339	2.2724
Error	5	0.46720654	0.093441	**Prob > F**
C. Total	6	0.67954511		0.1921

Parameter Estimates

Term	Estimate	Std Error	t Ratio	Prob>|t|
Intercept	5.6002733	0.208287	26.89	<.0001*
day	-0.087083	0.057768	-1.51	0.1921

Bivariate Fit of LnConc By day Oil=HCO, PAH=C2-DECALINS

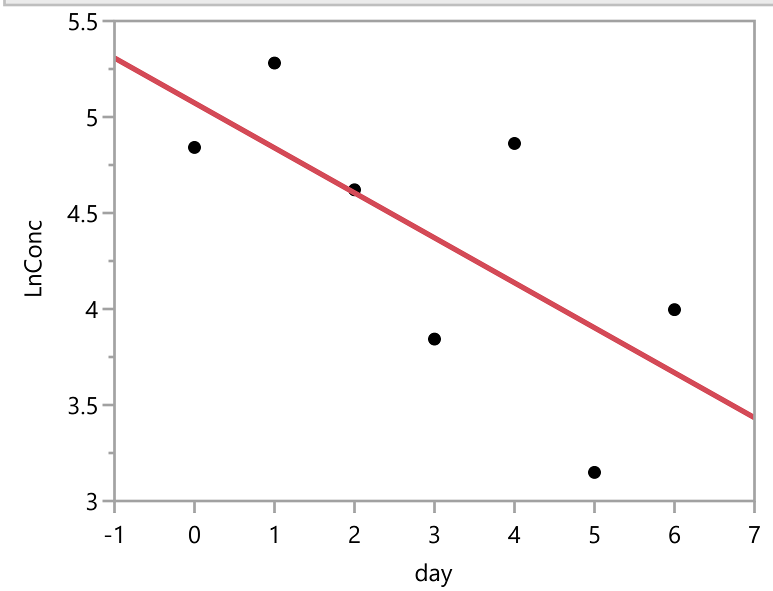

Linear Fit

LnConc = 5.0731772 - 0.2341917*day

Summary of Fit

RSquare	0.470334
RSquare Adj	0.3644
Root Mean Square Error	0.588117
Mean of Response	4.370602
Observations (or Sum Wgts)	7

Analysis of Variance

Source	DF	Sum of Squares	Mean Square	F Ratio
Model	1	1.5356807	1.53568	4.4399
Error	5	1.7294074	0.34588	**Prob > F**
C. Total	6	3.2650881		0.0889

Parameter Estimates

Term	Estimate	Std Error	t Ratio	Prob>|t|
Intercept	5.0731772	0.400734	12.66	<.0001*
day	-0.234192	0.111144	-2.11	0.0889

Bivariate Fit of LnConc By day Oil=HCO, PAH=C2-DIBENZOTHIOPHENES

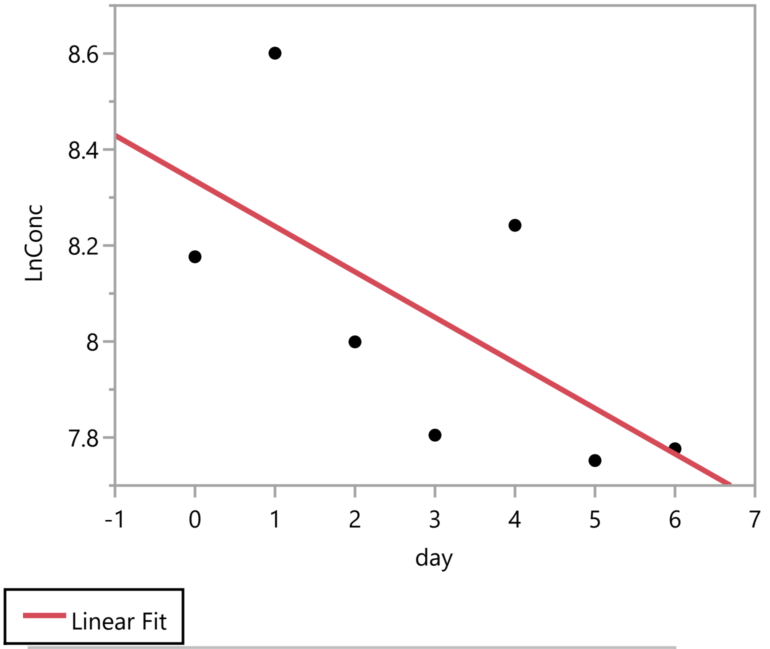

Linear Fit

LnConc = 8.3346553 - 0.0948037*day

Summary of Fit

RSquare	0.432117
RSquare Adj	0.31854
Root Mean Square Error	0.257187
Mean of Response	8.050244
Observations (or Sum Wgts)	7

Analysis of Variance

Source	DF	Sum of Squares	Mean Square	F Ratio
Model	1	0.25165681	0.251657	3.8046
Error	5	0.33072455	0.066145	**Prob > F**
C. Total	6	0.58238136		0.1086

Parameter Estimates

Term	Estimate	Std Error	t Ratio	Prob>|t|
Intercept	8.3346553	0.175243	47.56	<.0001*
day	-0.094804	0.048604	-1.95	0.1086

Bivariate Fit of LnConc By day
Oil=HCO, PAH=C2-FLUORANTHENES/PYRENES

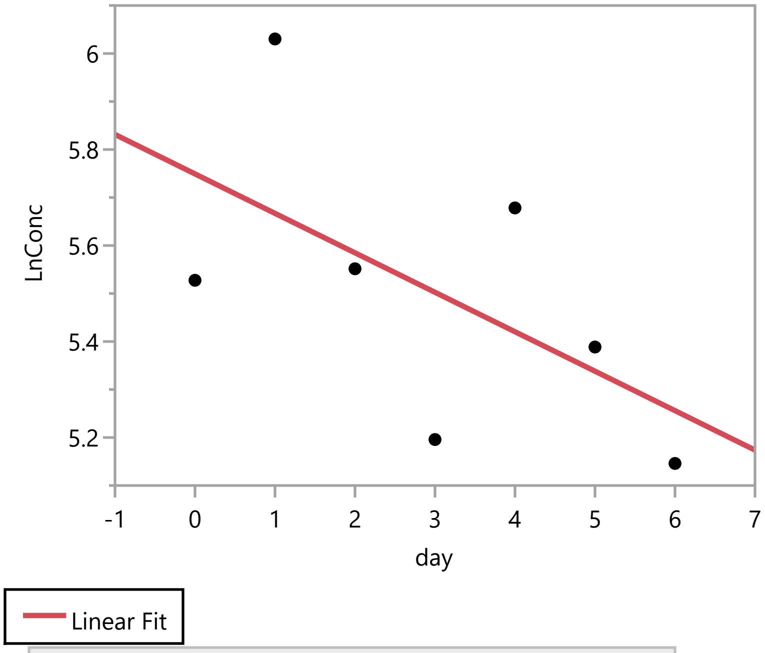

Linear Fit

LnConc = 5.7491404 - 0.0821997*day

Summary of Fit

RSquare	0.346038
RSquare Adj	0.215246
Root Mean Square Error	0.26741
Mean of Response	5.502541
Observations (or Sum Wgts)	7

Analysis of Variance

Source	DF	Sum of Squares	Mean Square	F Ratio
Model	1	0.18919020	0.189190	2.6457
Error	5	0.35754163	0.071508	**Prob > F**
C. Total	6	0.54673183		0.1648

Parameter Estimates

Term	Estimate	Std Error	t Ratio	Prob>|t|
Intercept	5.7491404	0.182209	31.55	<.0001*
day	-0.0822	0.050536	-1.63	0.1648

Bivariate Fit of LnConc By day Oil=HCO, PAH=C2-FLUORENES

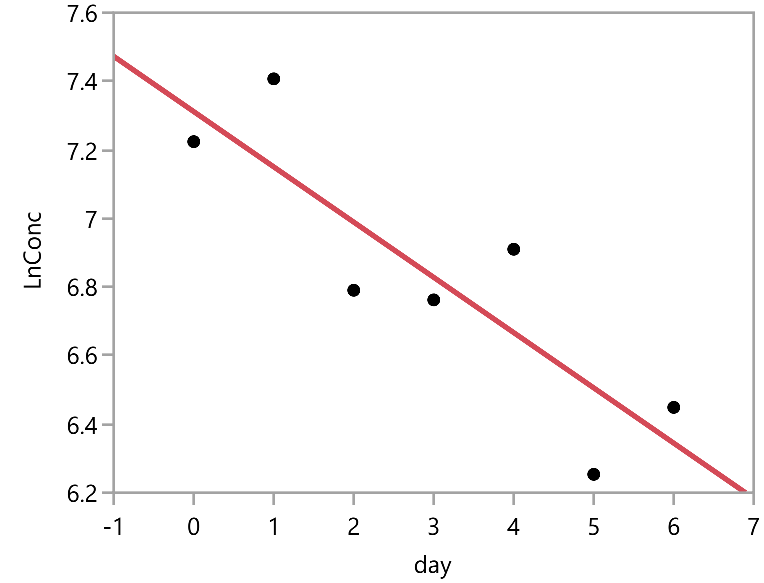

Linear Fit

Linear Fit

LnConc = 7.311673 - 0.1611692*day

Summary of Fit

RSquare	0.743431
RSquare Adj	0.692117
Root Mean Square Error	0.224057
Mean of Response	6.828166
Observations (or Sum Wgts)	7

Analysis of Variance

Source	DF	Sum of Squares	Mean Square	F Ratio
Model	1	0.72731393	0.727314	14.4879
Error	5	0.25100722	0.050201	**Prob > F**
C. Total	6	0.97832114		0.0125*

Parameter Estimates

Term	Estimate	Std Error	t Ratio	Prob>|t|
Intercept	7.311673	0.152669	47.89	<.0001*
day	-0.161169	0.042343	-3.81	0.0125*

Bivariate Fit of LnConc By day Oil=HCO, PAH=C2-NAPHTHALENES

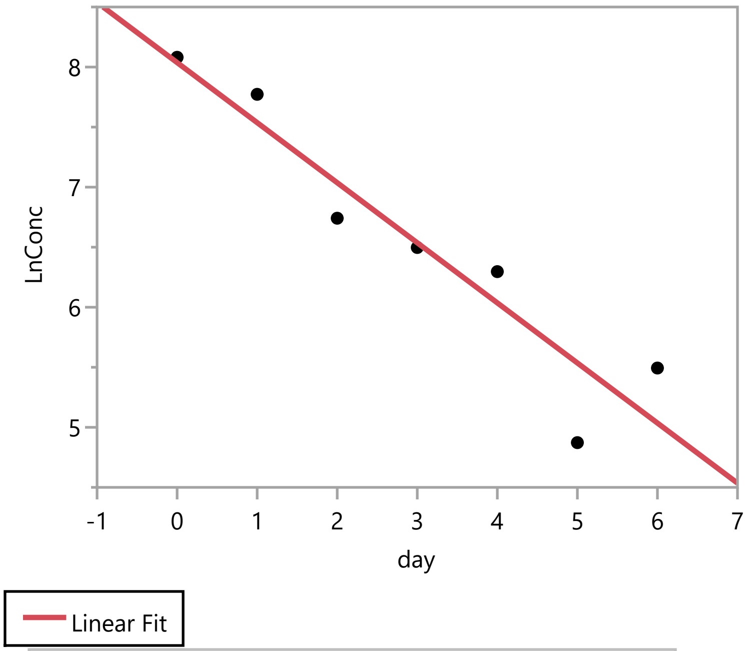

Linear Fit

LnConc = 8.0373484 - 0.5002833*day

Summary of Fit

RSquare	0.890233
RSquare Adj	0.868279
Root Mean Square Error	0.415714
Mean of Response	6.536498
Observations (or Sum Wgts)	7

Analysis of Variance

Source	DF	Sum of Squares	Mean Square	F Ratio
Model	1	7.0079350	7.00793	40.5509
Error	5	0.8640907	0.17282	**Prob > F**
C. Total	6	7.8720257		0.0014*

Parameter Estimates

Term	Estimate	Std Error	t Ratio	Prob>|t|
Intercept	8.0373484	0.283261	28.37	<.0001*
day	-0.500283	0.078563	-6.37	0.0014*

Bivariate Fit of LnConc By day
Oil=HCO, PAH=C2-NAPHTHOBENZOTHIOPHENES

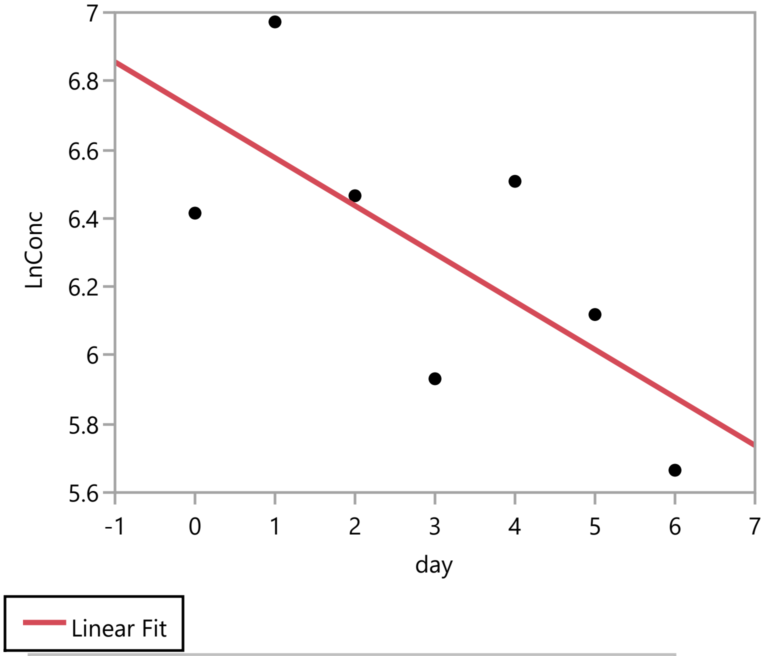

Linear Fit

LnConc = 6.7160178 - 0.1398071*day

Summary of Fit

RSquare	0.49411
RSquare Adj	0.392932
Root Mean Square Error	0.334764
Mean of Response	6.296597
Observations (or Sum Wgts)	7

Analysis of Variance

Source	DF	Sum of Squares	Mean Square	F Ratio
Model	1	0.5472884	0.547288	4.8836
Error	5	0.5603355	0.112067	**Prob > F**
C. Total	6	1.1076239		0.0781

Parameter Estimates

Term	Estimate	Std Error	t Ratio	Prob>|t|
Intercept	6.7160178	0.228103	29.44	<.0001*
day	-0.139807	0.063264	-2.21	0.0781

Bivariate Fit of LnConc By day Oil=HCO, PAH=C2-PHENANTHRENES/ANTHRACENES

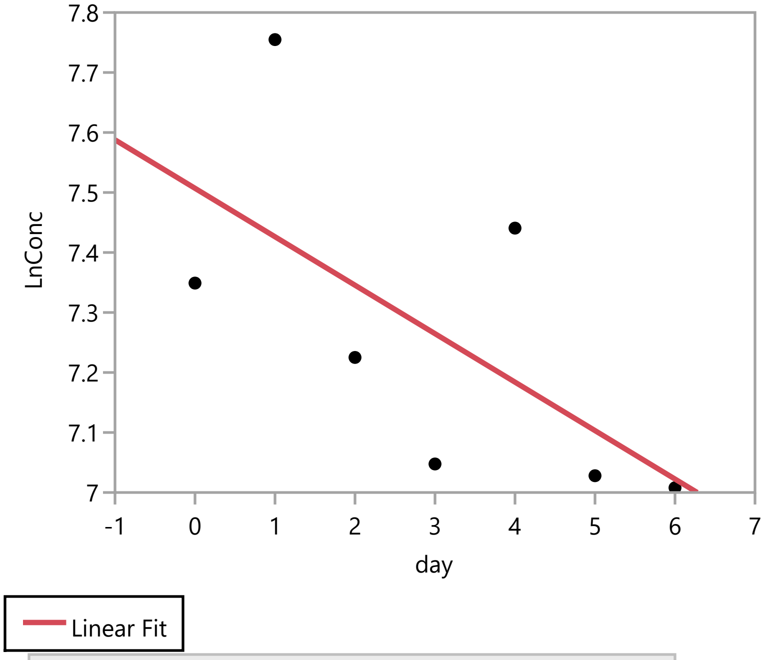

Linear Fit

LnConc = 7.5070119 - 0.0807693*day

Summary of Fit

RSquare	0.406814
RSquare Adj	0.288177
Root Mean Square Error	0.230801
Mean of Response	7.264704
Observations (or Sum Wgts)	7

Analysis of Variance

Source	DF	Sum of Squares	Mean Square	F Ratio
Model	1	0.18266292	0.182663	3.4291
Error	5	0.26634547	0.053269	**Prob > F**
C. Total	6	0.44900839		0.1233

Parameter Estimates

Term	Estimate	Std Error	t Ratio	Prob>|t|
Intercept	7.5070119	0.157264	47.73	<.0001*
day	-0.080769	0.043617	-1.85	0.1233

Bivariate Fit of LnConc By day
Oil=HCO, PAH=C3-BENZO(B)THIOPHENES

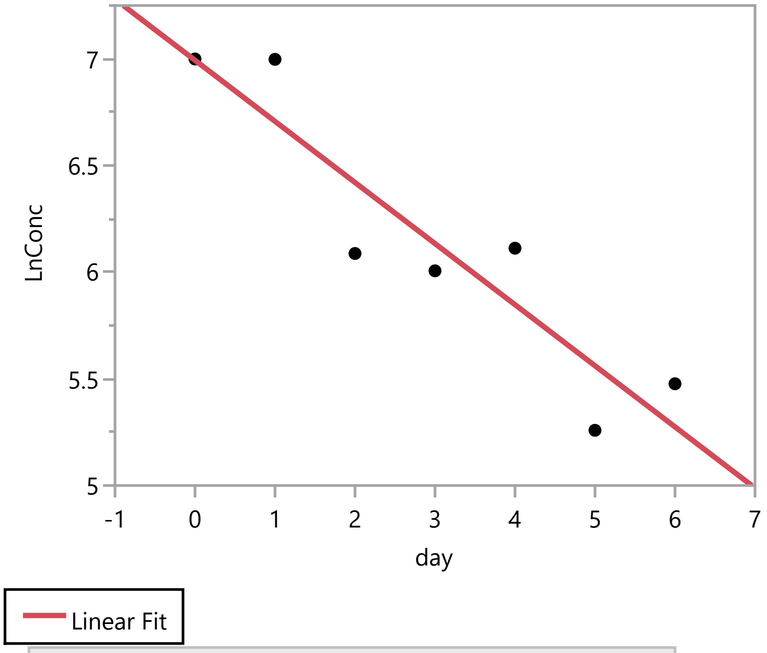

Linear Fit

LnConc = 6.9933265 - 0.2864218*day

Summary of Fit

RSquare	0.84726
RSquare Adj	0.816712
Root Mean Square Error	0.287785
Mean of Response	6.134061
Observations (or Sum Wgts)	7

Analysis of Variance

Source	DF	Sum of Squares	Mean Square	F Ratio
Model	1	2.2970482	2.29705	27.7354
Error	5	0.4141002	0.08282	**Prob > F**
C. Total	6	2.7111484		0.0033*

Parameter Estimates

Term	Estimate	Std Error	t Ratio	Prob>|t|
Intercept	6.9933265	0.196092	35.66	<.0001*
day	-0.286422	0.054386	-5.27	0.0033*

Bivariate Fit of LnConc By day Oil=HCO, PAH=C3-CHRYSENES

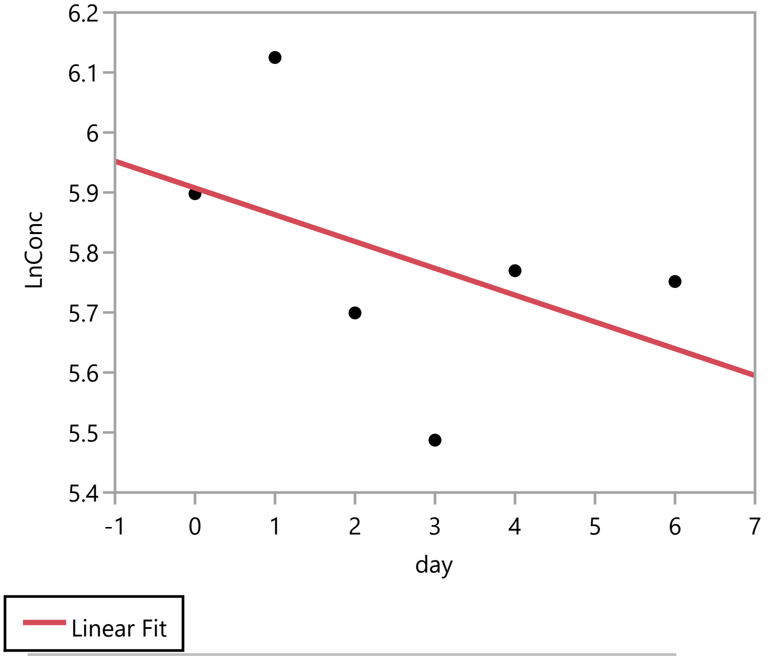

Linear Fit

LnConc = 5.9075378 - 0.0446488*day

Summary of Fit

RSquare	0.20614
RSquare Adj	0.007674
Root Mean Square Error	0.211622
Mean of Response	5.788474
Observations (or Sum Wgts)	6

Analysis of Variance

Source	DF	Sum of Squares	Mean Square	F Ratio
Model	1	0.04651543	0.046515	1.0387
Error	4	0.17913478	0.044784	**Prob > F**
C. Total	5	0.22565021		0.3658

Parameter Estimates

Term	Estimate	Std Error	t Ratio	Prob>|t|
Intercept	5.9075378	0.145301	40.66	<.0001*
day	-0.044649	0.04381	-1.02	0.3658

Bivariate Fit of LnConc By day Oil=HCO, PAH=C3-DECALINS

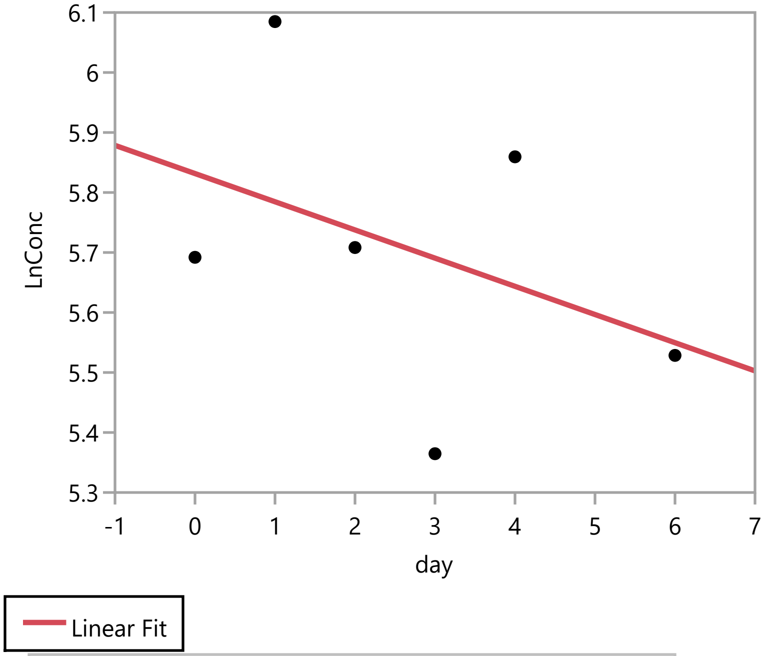

Linear Fit

LnConc = 5.8315814 - 0.0469864*day

Summary of Fit

RSquare	0.163376
RSquare Adj	-0.04578
Root Mean Square Error	0.256804
Mean of Response	5.706284
Observations (or Sum Wgts)	6

Analysis of Variance

Source	DF	Sum of Squares	Mean Square	F Ratio
Model	1	0.05151352	0.051514	0.7811
Error	4	0.26379367	0.065948	**Prob > F**
C. Total	5	0.31530719		0.4267

Parameter Estimates

Term	Estimate	Std Error	t Ratio	Prob>|t|
Intercept	5.8315814	0.176323	33.07	<.0001*
day	-0.046986	0.053164	-0.88	0.4267

Bivariate Fit of LnConc By day Oil=HCO, PAH=C3-DIBENZOTHIOPHENES

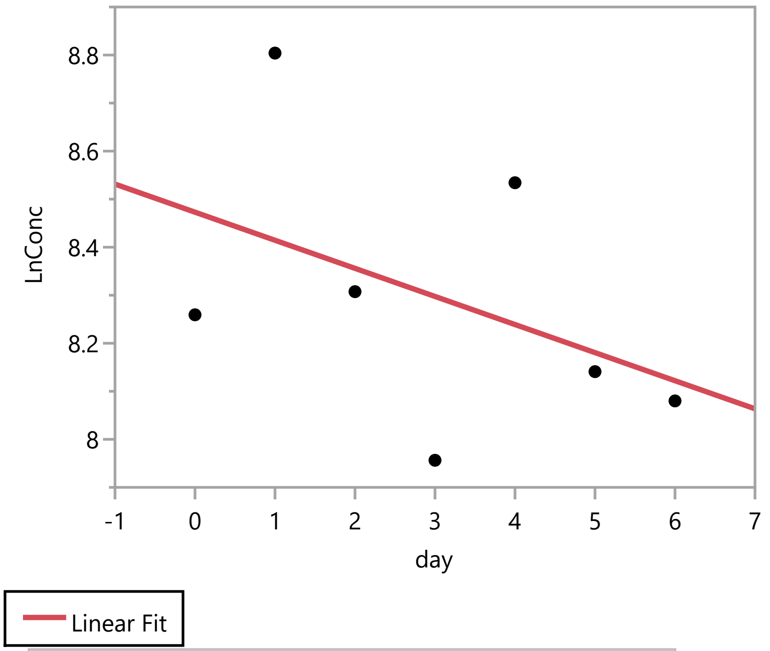

Linear Fit

LnConc = 8.4728857 - 0.0584755*day

Summary of Fit

RSquare	0.190586
RSquare Adj	0.028703
Root Mean Square Error	0.285173
Mean of Response	8.297459
Observations (or Sum Wgts)	7

Analysis of Variance

Source	DF	Sum of Squares	Mean Square	F Ratio
Model	1	0.09574279	0.095743	1.1773
Error	5	0.40661739	0.081323	**Prob > F**
C. Total	6	0.50236018		0.3274

Parameter Estimates

Term	Estimate	Std Error	t Ratio	Prob>|t|
Intercept	8.4728857	0.194312	43.60	<.0001*
day	-0.058476	0.053893	-1.09	0.3274

Bivariate Fit of LnConc By day
Oil=HCO, PAH=C3-FLUORANTHENES/PYRENES

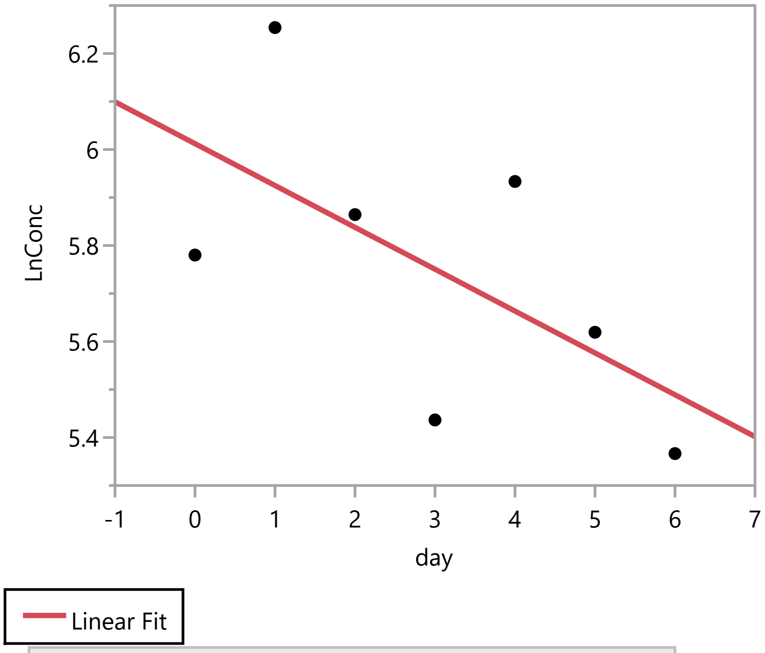

Linear Fit

LnConc = 6.012156 - 0.0871893*day

Summary of Fit

RSquare	0.377363
RSquare Adj	0.252836
Root Mean Square Error	0.26503
Mean of Response	5.750588
Observations (or Sum Wgts)	7

Analysis of Variance

Source	DF	Sum of Squares	Mean Square	F Ratio
Model	1	0.21285524	0.212855	3.0304
Error	5	0.35120390	0.070241	**Prob > F**
C. Total	6	0.56405913		0.1422

Parameter Estimates

Term	Estimate	Std Error	t Ratio	Prob>|t|
Intercept	6.012156	0.180587	33.29	<.0001*
day	-0.087189	0.050086	-1.74	0.1422

Bivariate Fit of LnConc By day Oil=HCO, PAH=C3-FLUORENES

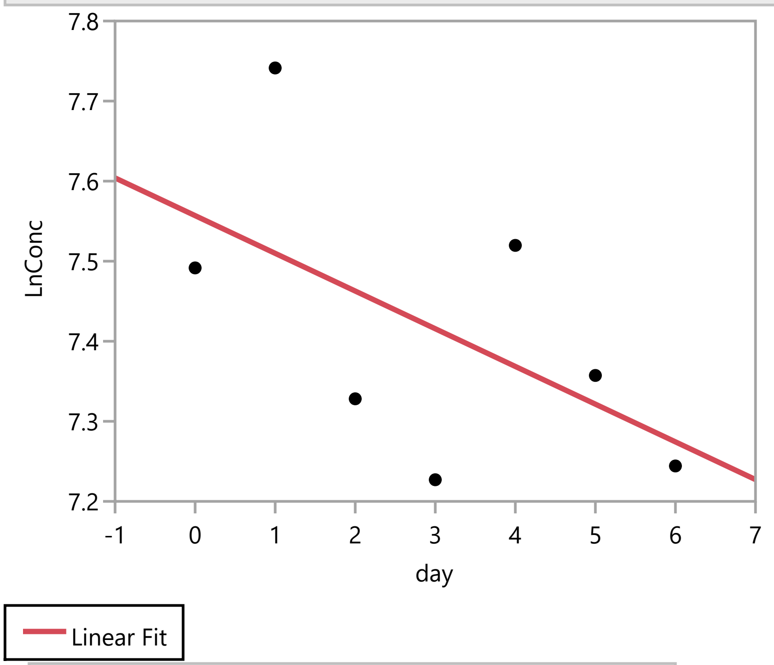

Linear Fit

LnConc = 7.5569122 - 0.0470966*day

Summary of Fit

RSquare	0.312523
RSquare Adj	0.175028
Root Mean Square Error	0.1653
Mean of Response	7.415622
Observations (or Sum Wgts)	7

Analysis of Variance

Source	DF	Sum of Squares	Mean Square	F Ratio
Model	1	0.06210657	0.062107	2.2730
Error	5	0.13661982	0.027324	**Prob > F**
C. Total	6	0.19872638		0.1920

Parameter Estimates

Term	Estimate	Std Error	t Ratio	Prob>|t|
Intercept	7.5569122	0.112633	67.09	<.0001*
day	-0.047097	0.031239	-1.51	0.1920

Bivariate Fit of LnConc By day Oil=HCO, PAH=C3-NAPHTHALENES

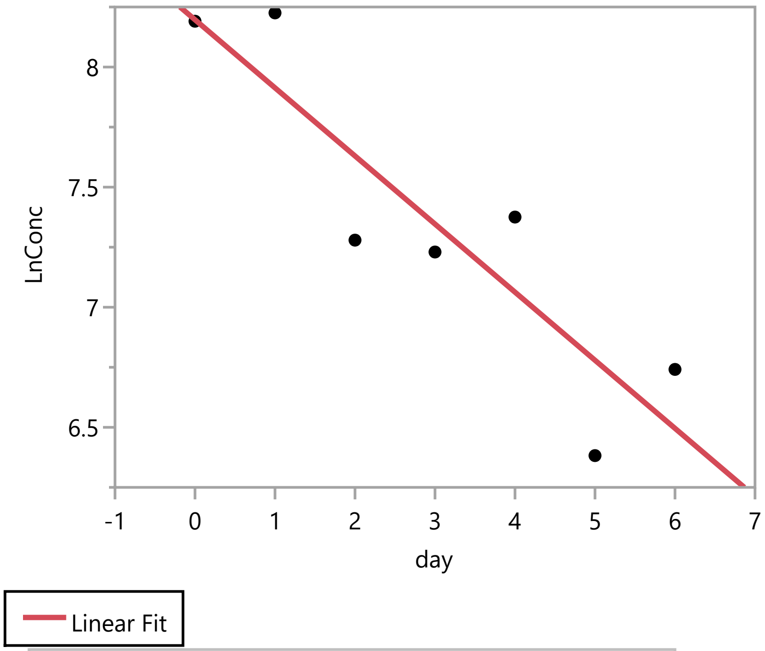

Linear Fit

LnConc = 8.1969781 - 0.2835456*day

Summary of Fit

RSquare	0.803819
RSquare Adj	0.764583
Root Mean Square Error	0.331487
Mean of Response	7.346341
Observations (or Sum Wgts)	7

Analysis of Variance

Source	DF	Sum of Squares	Mean Square	F Ratio
Model	1	2.2511477	2.25115	20.4867
Error	5	0.5494165	0.10988	**Prob > F**
C. Total	6	2.8005643		0.0062*

Parameter Estimates

Term	Estimate	Std Error	t Ratio	Prob>|t|
Intercept	8.1969781	0.22587	36.29	<.0001*
day	-0.283546	0.062645	-4.53	0.0062*

Bivariate Fit of LnConc By day
Oil=HCO, PAH=C3-NAPHTHOBENZOTHIOPHENES

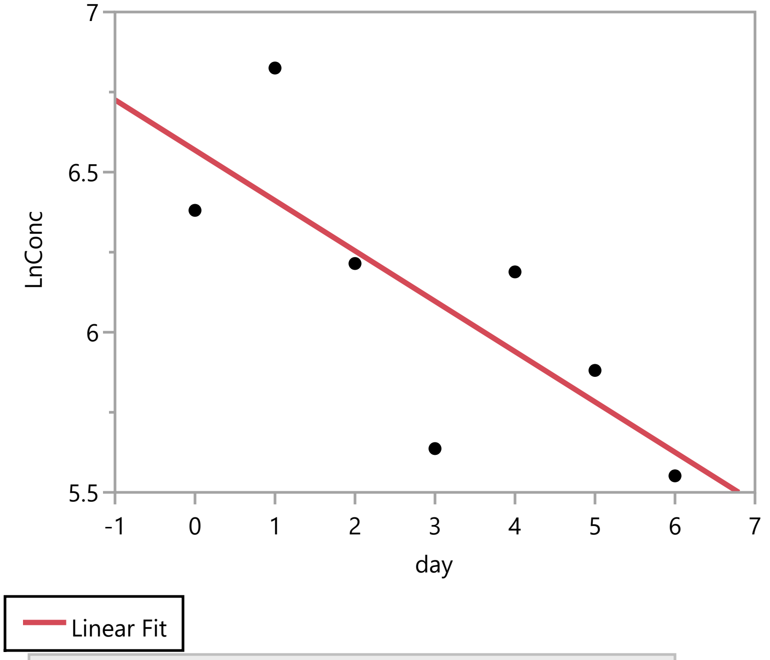

Linear Fit

LnConc = 6.5684591 - 0.1572059*day

Summary of Fit

RSquare	0.582077
RSquare Adj	0.498493
Root Mean Square Error	0.315225
Mean of Response	6.096841
Observations (or Sum Wgts)	7

Analysis of Variance

Source	DF	Sum of Squares	Mean Square	F Ratio
Model	1	0.6919836	0.691984	6.9639
Error	5	0.4968337	0.099367	**Prob > F**
C. Total	6	1.1888174		0.0460*

Parameter Estimates

Term	Estimate	Std Error	t Ratio	Prob>|t|
Intercept	6.5684591	0.21479	30.58	<.0001*
day	-0.157206	0.059572	-2.64	0.0460*

Bivariate Fit of LnConc By day Oil=HCO, PAH=C3-PHENANTHRENES/ANTHRACENES

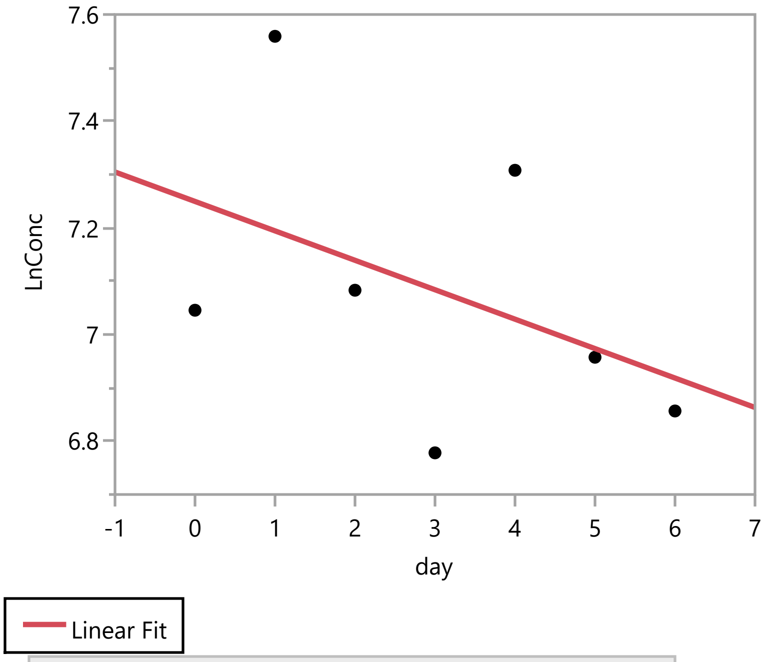

Linear Fit

LnConc = 7.2495023 - 0.0551896*day

Summary of Fit

RSquare	0.194424
RSquare Adj	0.033309
Root Mean Square Error	0.265845
Mean of Response	7.083934
Observations (or Sum Wgts)	7

Analysis of Variance

Source	DF	Sum of Squares	Mean Square	F Ratio
Model	1	0.08528487	0.085285	1.2067
Error	5	0.35336831	0.070674	**Prob > F**
C. Total	6	0.43865317		0.3220

Parameter Estimates

Term	Estimate	Std Error	t Ratio	Prob>|t|
Intercept	7.2495023	0.181143	40.02	<.0001*
day	-0.05519	0.05024	-1.10	0.3220

Bivariate Fit of LnConc By day
Oil=HCO, PAH=C4-BENZO(B)THIOPHENES

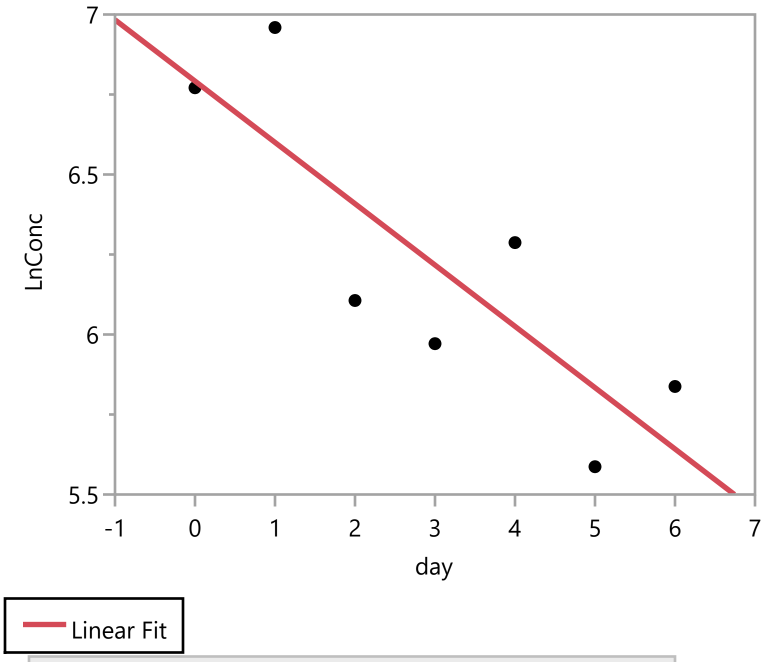

Linear Fit

LnConc = 6.791852 - 0.1915907*day

Summary of Fit

RSquare	0.69604
RSquare Adj	0.635248
Root Mean Square Error	0.299612
Mean of Response	6.21708
Observations (or Sum Wgts)	7

Analysis of Variance

Source	DF	Sum of Squares	Mean Square	F Ratio
Model	1	1.0277961	1.02780	11.4496
Error	5	0.4488369	0.08977	**Prob > F**
C. Total	6	1.4766330		0.0196*

Parameter Estimates

Term	Estimate	Std Error	t Ratio	Prob>|t|
Intercept	6.791852	0.204151	33.27	<.0001*
day	-0.191591	0.056621	-3.38	0.0196*

Bivariate Fit of LnConc By day Oil=HCO, PAH=C4-DECALINS

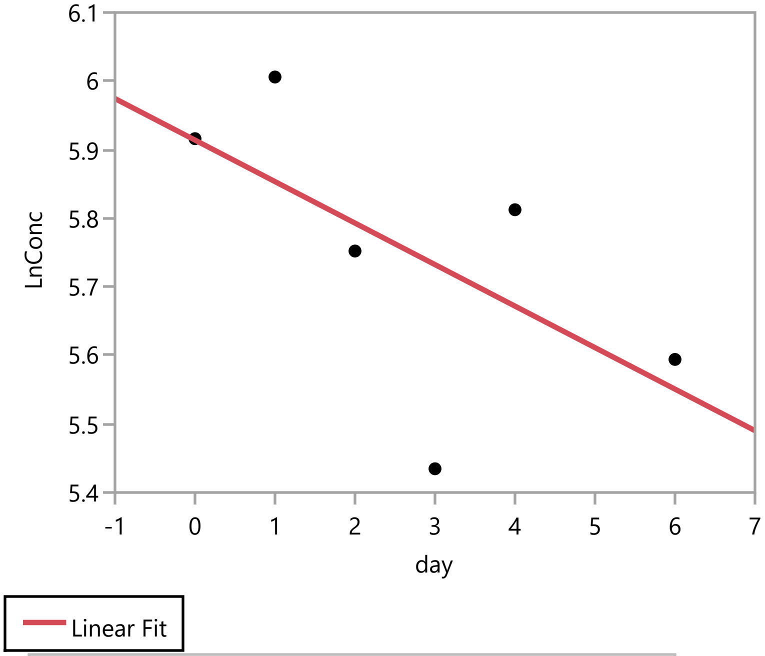

Linear Fit

LnConc = 5.9139318 - 0.0605317*day

Summary of Fit

RSquare	0.387399
RSquare Adj	0.234248
Root Mean Square Error	0.183845
Mean of Response	5.752514
Observations (or Sum Wgts)	6

Analysis of Variance

Source	DF	Sum of Squares	Mean Square	F Ratio
Model	1	0.08549548	0.085495	2.5295
Error	4	0.13519577	0.033799	**Prob > F**
C. Total	5	0.22069125		0.1869

Parameter Estimates

Term	Estimate	Std Error	t Ratio	Prob>|t|
Intercept	5.9139318	0.126229	46.85	<.0001*
day	-0.060532	0.03806	-1.59	0.1869

Bivariate Fit of LnConc By day Oil=HCO, PAH=C4-DIBENZOTHIOPHENES

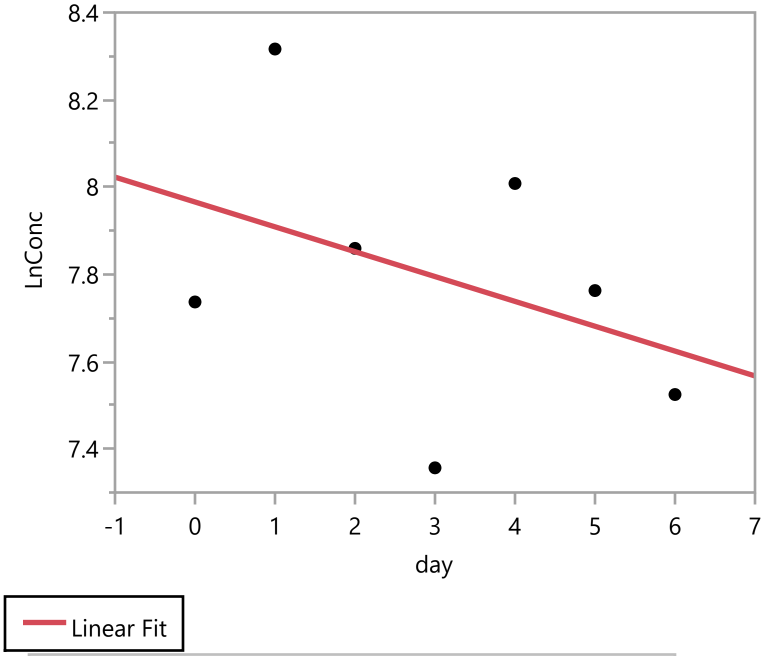

Linear Fit

LnConc = 7.9657621 - 0.0569531*day

Summary of Fit

RSquare	0.153624
RSquare Adj	-0.01565
Root Mean Square Error	0.316347
Mean of Response	7.794903
Observations (or Sum Wgts)	7

Analysis of Variance

Source	DF	Sum of Squares	Mean Square	F Ratio
Model	1	0.09082241	0.090822	0.9075
Error	5	0.50037630	0.100075	**Prob > F**
C. Total	6	0.59119871		0.3845

Parameter Estimates

Term	Estimate	Std Error	t Ratio	Prob>|t|
Intercept	7.9657621	0.215554	36.95	<.0001*
day	-0.056953	0.059784	-0.95	0.3845

Bivariate Fit of LnConc By day
Oil=HCO, PAH=C4-FLUORANTHENES/PYRENES

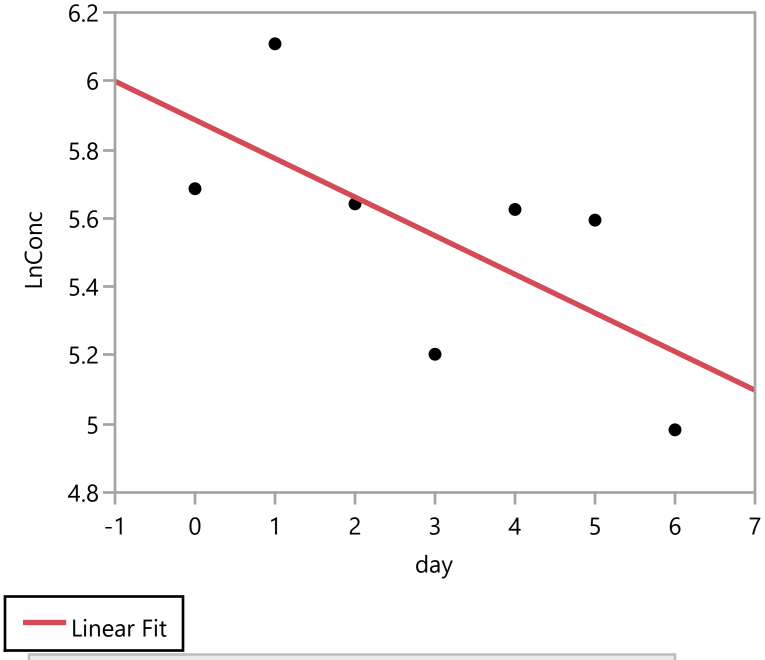

Linear Fit

LnConc = 5.8868991 - 0.112658*day

Summary of Fit

RSquare	0.450597
RSquare Adj	0.340716
Root Mean Square Error	0.29438
Mean of Response	5.548925
Observations (or Sum Wgts)	7

Analysis of Variance

Source	DF	Sum of Squares	Mean Square	F Ratio
Model	1	0.35537094	0.355371	4.1008
Error	5	0.43329663	0.086659	**Prob > F**
C. Total	6	0.78866758		0.0987

Parameter Estimates

Term	Estimate	Std Error	t Ratio	Prob>|t|
Intercept	5.8868991	0.200586	29.35	<.0001*
day	-0.112658	0.055633	-2.03	0.0987

Bivariate Fit of LnConc By day Oil=HCO, PAH=C4-NAPHTHALENES

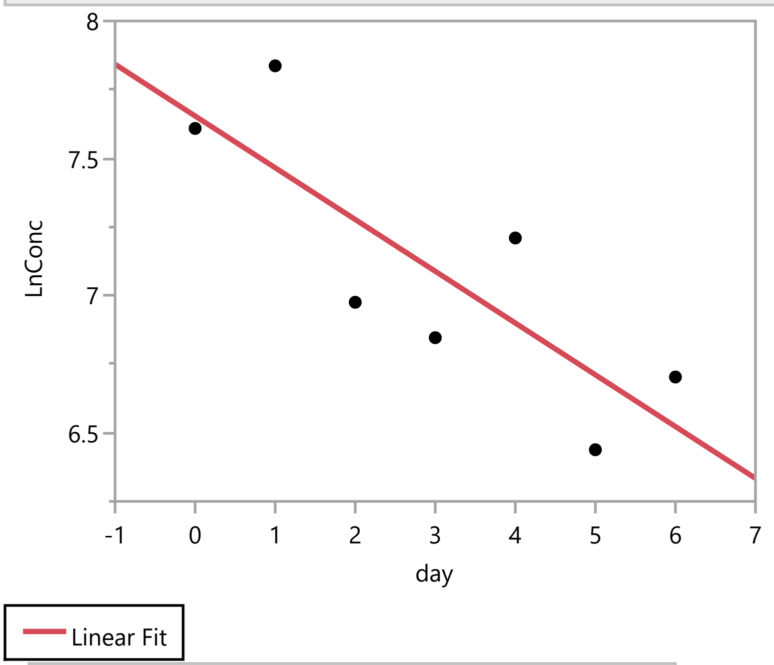

Linear Fit

LnConc = 7.6539513 - 0.1885703*day

Summary of Fit

RSquare	0.669041
RSquare Adj	0.602849
Root Mean Square Error	0.313854
Mean of Response	7.08824
Observations (or Sum Wgts)	7

Analysis of Variance

Source	DF	Sum of Squares	Mean Square	F Ratio
Model	1	0.9956455	0.995646	10.1076
Error	5	0.4925224	0.098504	**Prob > F**
C. Total	6	1.4881679		0.0246*

Parameter Estimates

Term	Estimate	Std Error	t Ratio	Prob>|t|
Intercept	7.6539513	0.213856	35.79	<.0001*
day	-0.18857	0.059313	-3.18	0.0246*

Bivariate Fit of LnConc By day
Oil=HCO, PAH=C4-NAPHTHOBENZOTHIOPHENES

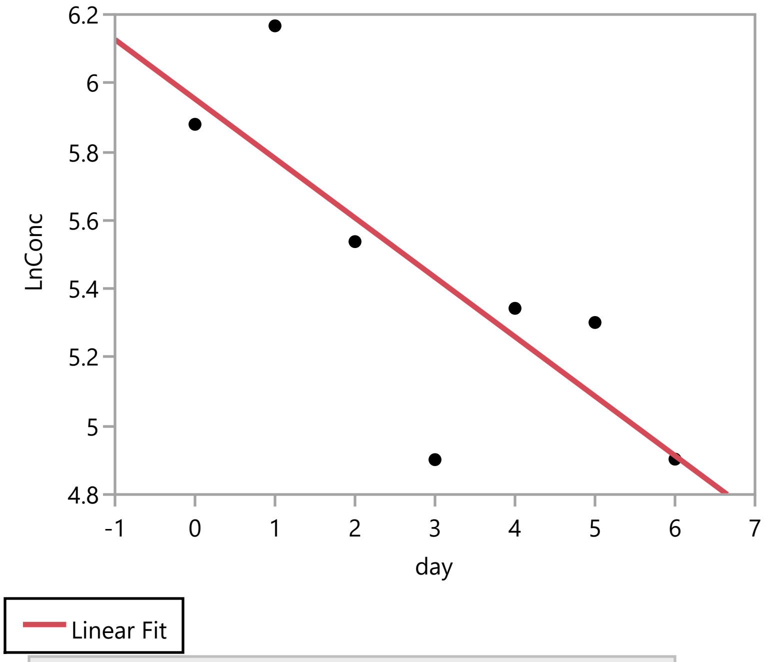

Linear Fit

LnConc = 5.9535459 - 0.1733893*day

Summary of Fit

RSquare	0.629327
RSquare Adj	0.555192
Root Mean Square Error	0.314901
Mean of Response	5.433378
Observations (or Sum Wgts)	7

Analysis of Variance

Source	DF	Sum of Squares	Mean Square	F Ratio
Model	1	0.8417881	0.841788	8.4890
Error	5	0.4958127	0.099163	**Prob > F**
C. Total	6	1.3376008		0.0333*

Parameter Estimates

Term	Estimate	Std Error	t Ratio	Prob>|t|
Intercept	5.9535459	0.214569	27.75	<.0001*
day	-0.173389	0.059511	-2.91	0.0333*

Bivariate Fit of LnConc By day Oil=HCO, PAH=C4-PHENANTHRENES/ANTHRACENES

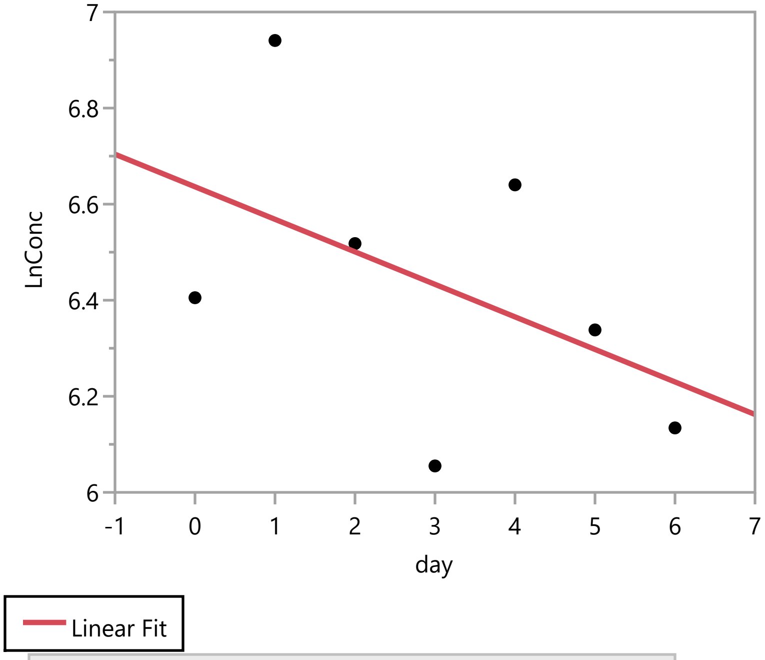

Linear Fit

LnConc = 6.6361866 - 0.0677106*day

Summary of Fit

RSquare	0.233479
RSquare Adj	0.080175
Root Mean Square Error	0.290328
Mean of Response	6.433055
Observations (or Sum Wgts)	7

Analysis of Variance

Source	DF	Sum of Squares	Mean Square	F Ratio
Model	1	0.12837218	0.128372	1.5230
Error	5	0.42145072	0.084290	**Prob > F**
C. Total	6	0.54982290		0.2720

Parameter Estimates

Term	Estimate	Std Error	t Ratio	Prob>|t|
Intercept	6.6361866	0.197825	33.55	<.0001*
day	-0.067711	0.054867	-1.23	0.2720

Bivariate Fit of LnConc By day
Oil=HCO, PAH=CHRYSENE/TRIPHENYLENE

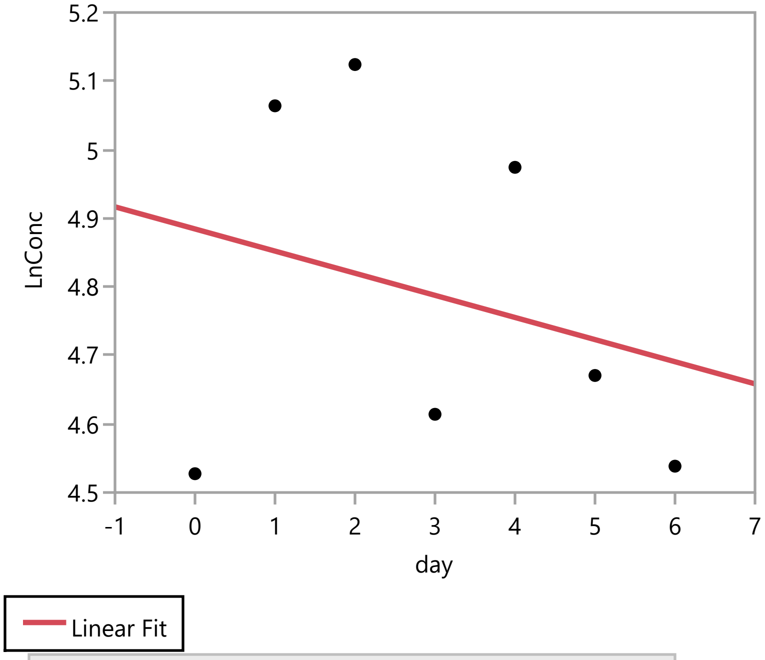

Linear Fit

LnConc = 4.88434 - 0.0322688*day

Summary of Fit

RSquare	0.073252
RSquare Adj	-0.1121
Root Mean Square Error	0.271611
Mean of Response	4.787534
Observations (or Sum Wgts)	7

Analysis of Variance

Source	DF	Sum of Squares	Mean Square	F Ratio
Model	1	0.02915569	0.029156	0.3952
Error	5	0.36886390	0.073773	**Prob > F**
C. Total	6	0.39801959		0.5572

Parameter Estimates

Term	Estimate	Std Error	t Ratio	Prob>|t|
Intercept	4.88434	0.185072	26.39	<.0001*
day	-0.032269	0.05133	-0.63	0.5572

Bivariate Fit of LnConc By day Oil=HCO, PAH=CIS/TRANS-DECALIN

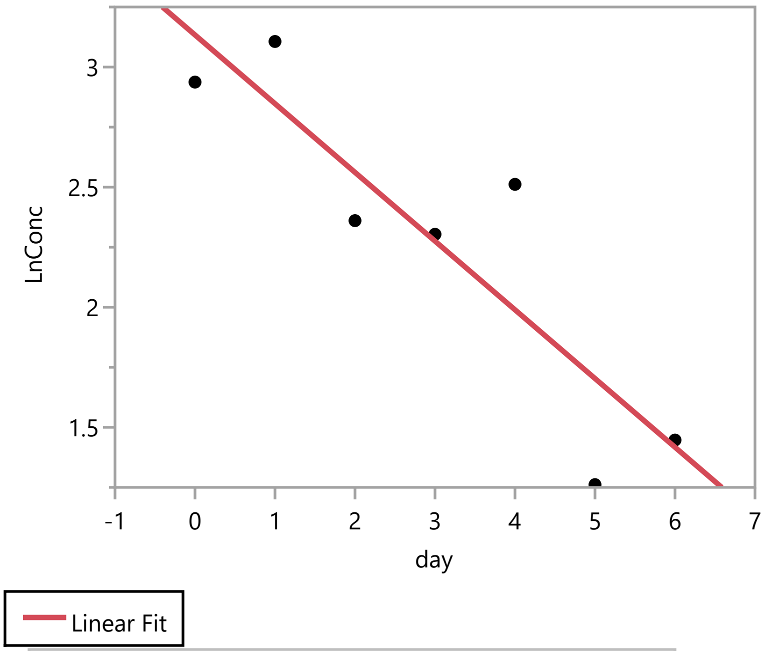

Linear Fit

LnConc = 3.1336677 - 0.2860793*day

Summary of Fit

RSquare	0.788234
RSquare Adj	0.745881
Root Mean Square Error	0.350898
Mean of Response	2.27543
Observations (or Sum Wgts)	7

Analysis of Variance

Source	DF	Sum of Squares	Mean Square	F Ratio
Model	1	2.2915583	2.29156	18.6110
Error	5	0.6156472	0.12313	**Prob > F**
C. Total	6	2.9072055		0.0076*

Parameter Estimates

Term	Estimate	Std Error	t Ratio	Prob>|t|
Intercept	3.1336677	0.239097	13.11	<.0001*
day	-0.286079	0.066313	-4.31	0.0076*

Bivariate Fit of LnConc By day Oil=HCO, PAH=DIBENZOFURAN

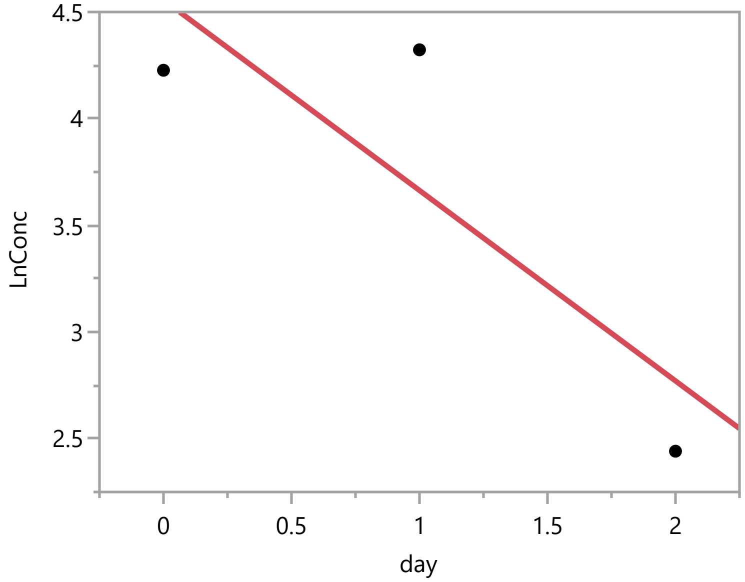

Linear Fit

Linear Fit

LnConc = 4.5564481 - 0.8928473*day

Summary of Fit

RSquare	0.709832
RSquare Adj	0.419664
Root Mean Square Error	0.807308
Mean of Response	3.663601
Observations (or Sum Wgts)	3

Analysis of Variance

Source	DF	Sum of Squares	Mean Square	F Ratio
Model	1	1.5943527	1.59435	2.4463
Error	1	0.6517466	0.65175	**Prob > F**
C. Total	2	2.2460992		0.3621

Parameter Estimates

Term	Estimate	Std Error	t Ratio	Prob>|t|
Intercept	4.5564481	0.736968	6.18	0.1021
day	-0.892847	0.570853	-1.56	0.3621

Bivariate Fit of LnConc By day Oil=HCO, PAH=DIBENZOTHIOPHENE

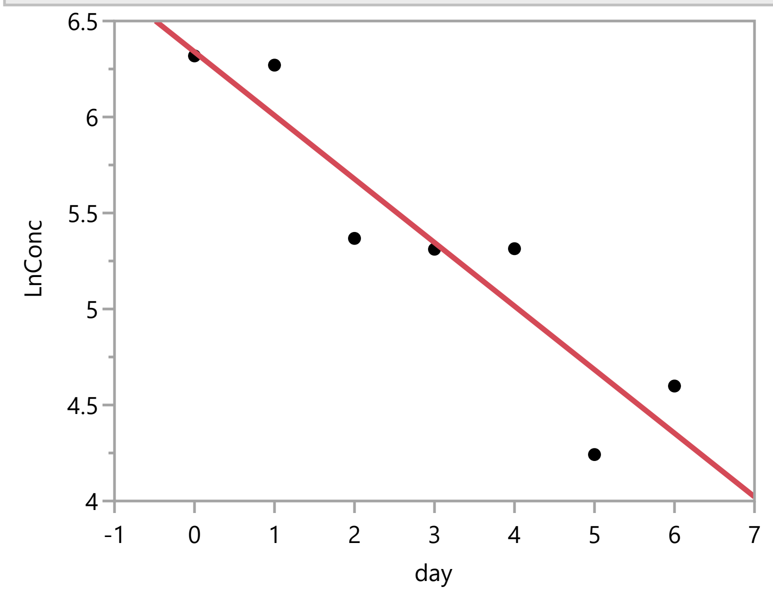

Linear Fit

LnConc = 6.339064 - 0.3310151*day

Summary of Fit

RSquare	0.857148
RSquare Adj	0.828578
Root Mean Square Error	0.319784
Mean of Response	5.346019
Observations (or Sum Wgts)	7

Analysis of Variance

Source	DF	Sum of Squares	Mean Square	F Ratio
Model	1	3.0679872	3.06799	30.0012
Error	5	0.5113100	0.10226	**Prob > F**
C. Total	6	3.5792971		0.0028*

Parameter Estimates

Term	Estimate	Std Error	t Ratio	Prob>|t|
Intercept	6.339064	0.217896	29.09	<.0001*
day	-0.331015	0.060434	-5.48	0.0028*

Bivariate Fit of LnConc By day Oil=HCO, PAH=FLUORANTHENE

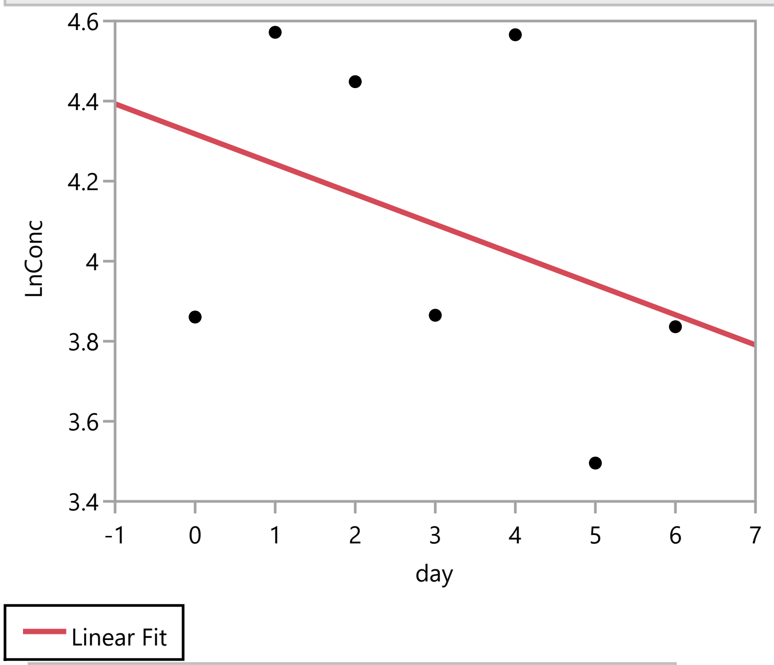

Linear Fit

LnConc = 4.3177292 - 0.0752738*day

Summary of Fit

RSquare	0.143212
RSquare Adj	-0.02815
Root Mean Square Error	0.435697
Mean of Response	4.091908
Observations (or Sum Wgts)	7

Analysis of Variance

Source	DF	Sum of Squares	Mean Square	F Ratio
Model	1	0.1586521	0.158652	0.8357
Error	5	0.9491608	0.189832	**Prob > F**
C. Total	6	1.1078129		0.4025

Parameter Estimates

Term	Estimate	Std Error	t Ratio	Prob>|t|
Intercept	4.3177292	0.296878	14.54	<.0001*
day	-0.075274	0.082339	-0.91	0.4025

Bivariate Fit of LnConc By day Oil=HCO, PAH=FLUORENE

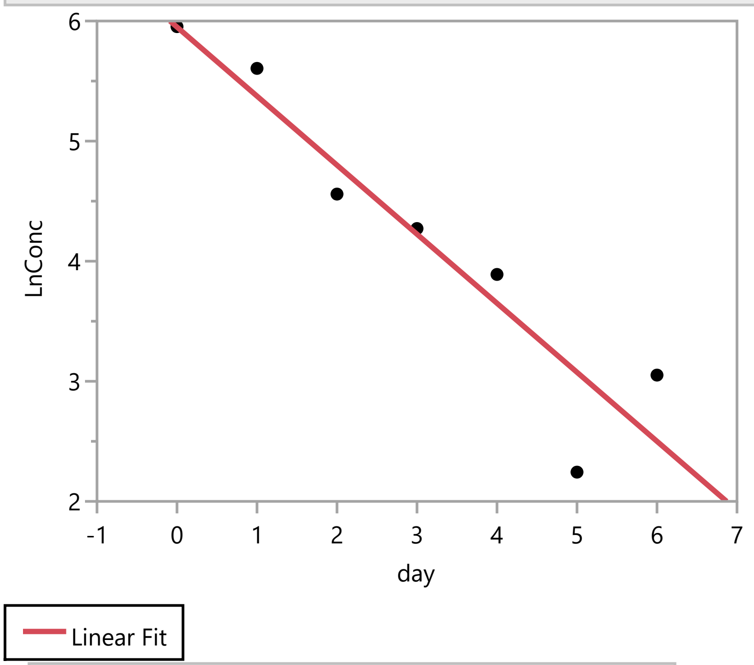

Linear Fit

LnConc = 5.9497358 - 0.5749486*day

Summary of Fit

RSquare	0.888065
RSquare Adj	0.865678
Root Mean Square Error	0.483041
Mean of Response	4.22489
Observations (or Sum Wgts)	7

Analysis of Variance

Source	DF	Sum of Squares	Mean Square	F Ratio
Model	1	9.255845	9.25584	39.6687
Error	5	1.166643	0.23333	**Prob > F**
C. Total	6	10.422488		0.0015*

Parameter Estimates

Term	Estimate	Std Error	t Ratio	Prob>|t|
Intercept	5.9497358	0.329137	18.08	<.0001*
day	-0.574949	0.091286	-6.30	0.0015*

Bivariate Fit of LnConc By day Oil=HCO, PAH=NAPHTHALENE

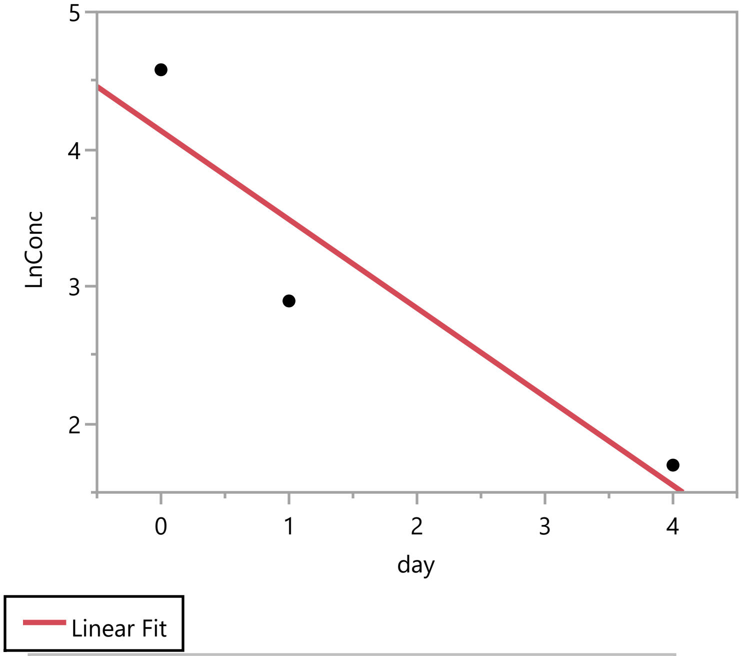

Linear Fit

LnConc = 4.134069 - 0.6459718*day

Summary of Fit

RSquare	0.863382
RSquare Adj	0.726765
Root Mean Square Error	0.75647
Mean of Response	3.057449
Observations (or Sum Wgts)	3

Analysis of Variance

Source	DF	Sum of Squares	Mean Square	F Ratio
Model	1	3.6164224	3.61642	6.3197
Error	1	0.5722463	0.57225	**Prob > F**
C. Total	2	4.1886687		0.2410

Parameter Estimates

Term	Estimate	Std Error	t Ratio	Prob>|t|
Intercept	4.134069	0.611687	6.76	0.0935
day	-0.645972	0.25696	-2.51	0.2410

Bivariate Fit of LnConc By day
Oil=HCO, PAH=NAPHTHOBENZOTHIOPHENE

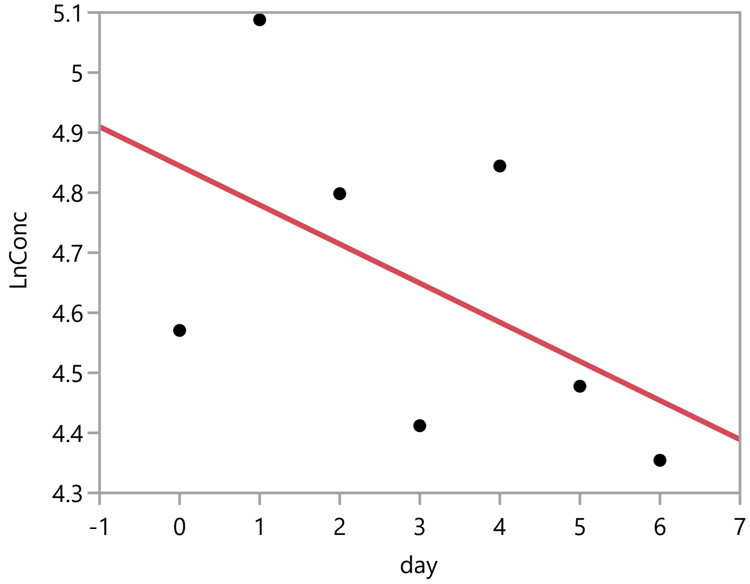

Linear Fit

LnConc = 4.8446386 - 0.0651118*day

Summary of Fit

RSquare	0.274978
RSquare Adj	0.129974
Root Mean Square Error	0.250196
Mean of Response	4.649303
Observations (or Sum Wgts)	7

Analysis of Variance

Source	DF	Sum of Squares	Mean Square	F Ratio
Model	1	0.11870742	0.118707	1.8963
Error	5	0.31299032	0.062598	**Prob > F**
C. Total	6	0.43169774		0.2269

Parameter Estimates

Term	Estimate	Std Error	t Ratio	Prob>|t|
Intercept	4.8446386	0.17048	28.42	<.0001*
day	-0.065112	0.047283	-1.38	0.2269

Bivariate Fit of LnConc By day Oil=HCO, PAH=PERYLENE

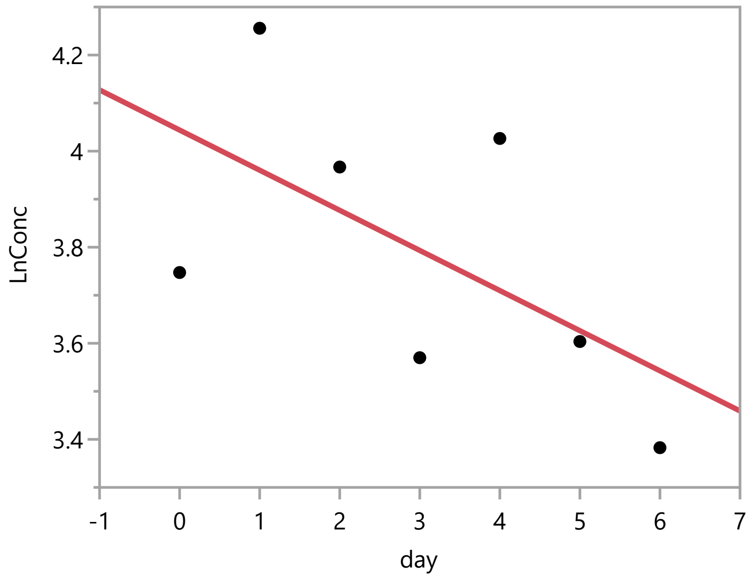

Linear Fit

LnConc = 4.0438245 - 0.0835195*day

Summary of Fit

RSquare	0.35201
RSquare Adj	0.222412
Root Mean Square Error	0.268157
Mean of Response	3.793266
Observations (or Sum Wgts)	7

Analysis of Variance

Source	DF	Sum of Squares	Mean Square	F Ratio
Model	1	0.19531415	0.195314	2.7162
Error	5	0.35953969	0.071908	**Prob > F**
C. Total	6	0.55485384		0.1603

Parameter Estimates

Term	Estimate	Std Error	t Ratio	Prob>|t|
Intercept	4.0438245	0.182718	22.13	<.0001*
day	-0.083519	0.050677	-1.65	0.1603

Bivariate Fit of LnConc By day Oil=HCO, PAH=PHENANTHRENE

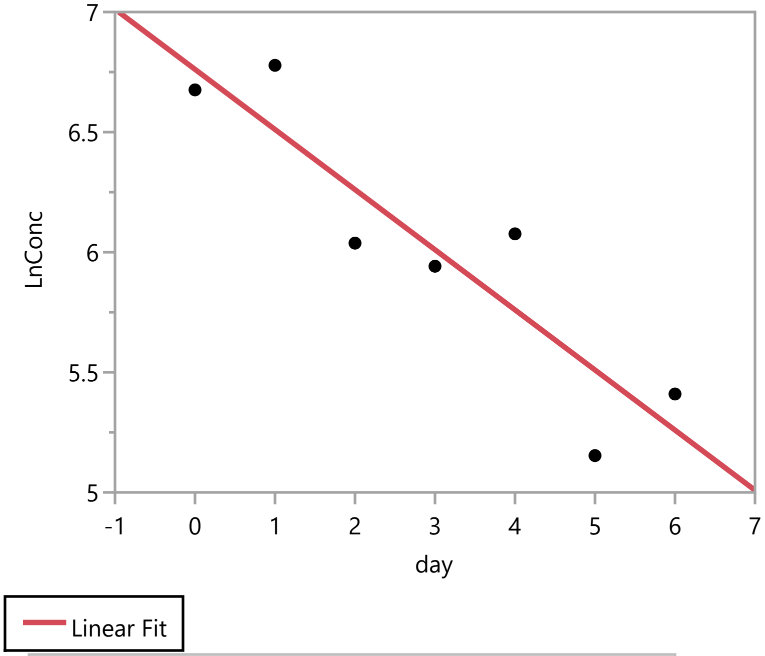

Linear Fit

LnConc = 6.761334 - 0.2503428*day

Summary of Fit

RSquare	0.820888
RSquare Adj	0.785066
Root Mean Square Error	0.276726
Mean of Response	6.010306
Observations (or Sum Wgts)	7

Analysis of Variance

Source	DF	Sum of Squares	Mean Square	F Ratio
Model	1	1.7548025	1.75480	22.9155
Error	5	0.3828853	0.07658	**Prob > F**
C. Total	6	2.1376878		0.0049*

Parameter Estimates

Term	Estimate	Std Error	t Ratio	Prob>|t|
Intercept	6.761334	0.188557	35.86	<.0001*
day	-0.250343	0.052296	-4.79	0.0049*

Bivariate Fit of LnConc By day Oil=HCO, PAH=PYRENE

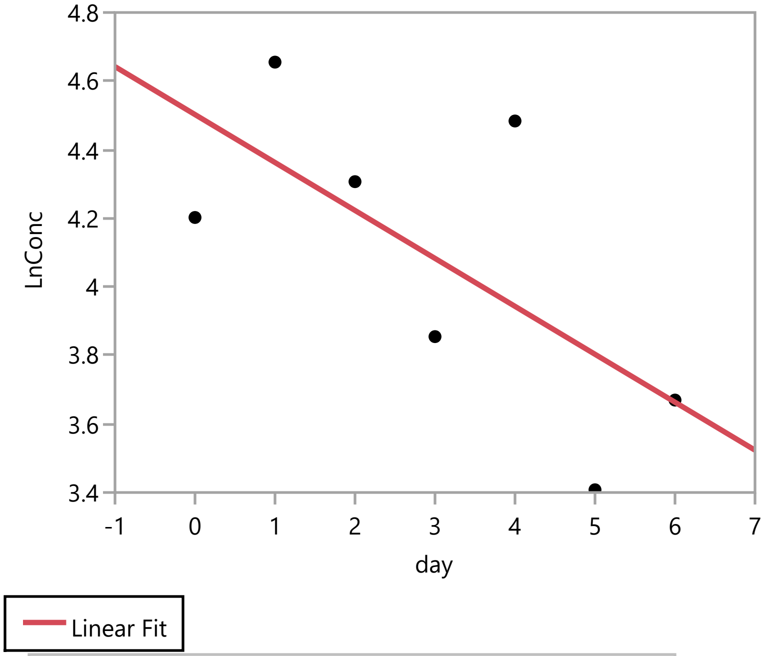

Linear Fit

LnConc = 4.5023063 - 0.1398318*day

Summary of Fit

RSquare	0.444801
RSquare Adj	0.333761
Root Mean Square Error	0.369693
Mean of Response	4.082811
Observations (or Sum Wgts)	7

Analysis of Variance

Source	DF	Sum of Squares	Mean Square	F Ratio
Model	1	0.5474820	0.547482	4.0058
Error	5	0.6833655	0.136673	**Prob > F**
C. Total	6	1.2308476		0.1018

Parameter Estimates

Term	Estimate	Std Error	t Ratio	Prob>|t|
Intercept	4.5023063	0.251903	17.87	<.0001*
day	-0.139832	0.069865	-2.00	0.1018

Bivariate Fit of LnConc By day Oil=HFO, PAH=ACENAPHTHENE

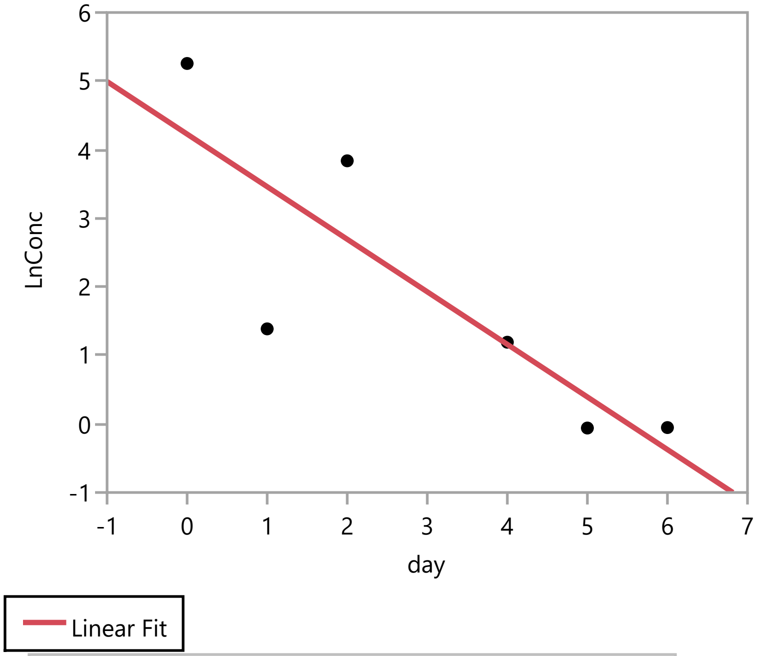

Linear Fit

LnConc = 4.2264709 - 0.7666825*day

Summary of Fit

RSquare	0.702247
RSquare Adj	0.627809
Root Mean Square Error	1.320832
Mean of Response	1.926424
Observations (or Sum Wgts)	6

Analysis of Variance

Source	DF	Sum of Squares	Mean Square	F Ratio
Model	1	16.458455	16.4585	9.4340
Error	4	6.978386	1.7446	**Prob > F**
C. Total	5	23.436842		0.0372*

Parameter Estimates

Term	Estimate	Std Error	t Ratio	Prob>|t|
Intercept	4.2264709	0.922783	4.58	0.0102*
day	-0.766682	0.249614	-3.07	0.0372*

Bivariate Fit of LnConc By day Oil=HFO, PAH=ANTHRACENE

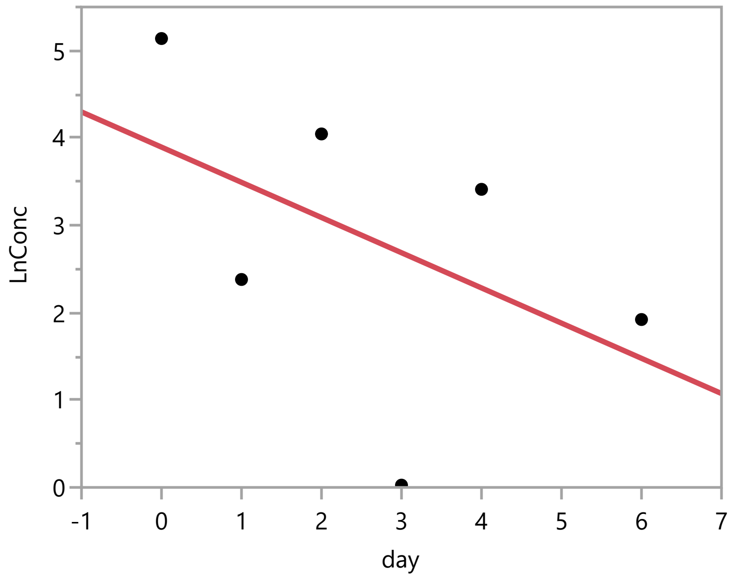

Linear Fit

LnConc = 3.895884 - 0.4030108*day

Summary of Fit

RSquare	0.236271
RSquare Adj	0.045339
Root Mean Square Error	1.750007
Mean of Response	2.821188
Observations (or Sum Wgts)	6

Analysis of Variance

Source	DF	Sum of Squares	Mean Square	F Ratio
Model	1	3.789747	3.78975	1.2375
Error	4	12.250093	3.06252	**Prob > F**
C. Total	5	16.039840		0.3283

Parameter Estimates

Term	Estimate	Std Error	t Ratio	Prob>|t|
Intercept	3.895884	1.201566	3.24	0.0316*
day	-0.403011	0.362286	-1.11	0.3283

Bivariate Fit of LnConc By day Oil=HFO, PAH=BENZ(A)ANTHRACENE

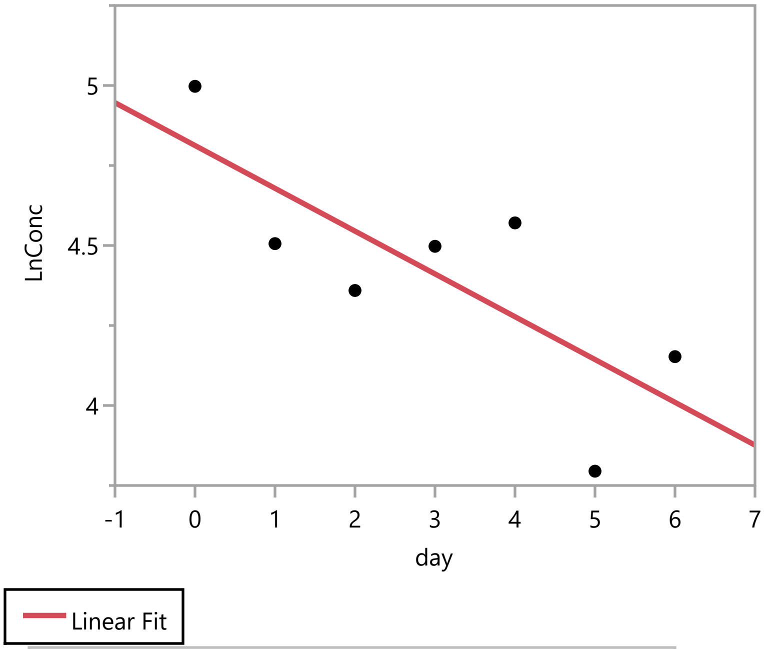

Linear Fit

LnConc = 4.8124487 - 0.1337603*day

Summary of Fit

RSquare	0.599911
RSquare Adj	0.519893
Root Mean Square Error	0.258497
Mean of Response	4.411168
Observations (or Sum Wgts)	7

Analysis of Variance

Source	DF	Sum of Squares	Mean Square	F Ratio
Model	1	0.50097060	0.500971	7.4972
Error	5	0.33410438	0.066821	**Prob > F**
C. Total	6	0.83507497		0.0409*

Parameter Estimates

Term	Estimate	Std Error	t Ratio	Prob>|t|
Intercept	4.8124487	0.176136	27.32	<.0001*
day	-0.13376	0.048851	-2.74	0.0409*

Bivariate Fit of LnConc By day Oil=HFO, PAH=BENZO(B)FLUORENE

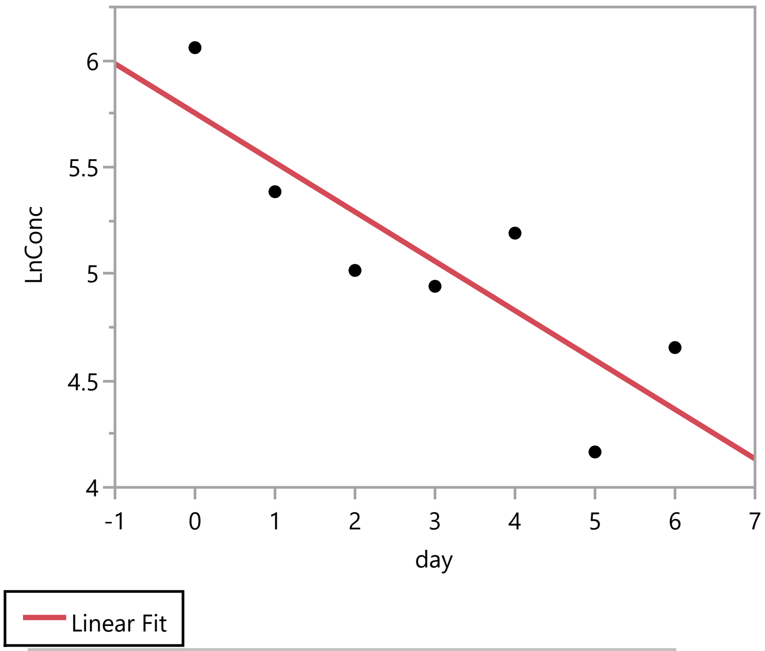

Linear Fit

LnConc = 5.7531546 - 0.2313401*day

Summary of Fit

RSquare	0.713134
RSquare Adj	0.655761
Root Mean Square Error	0.347215
Mean of Response	5.059134
Observations (or Sum Wgts)	7

Analysis of Variance

Source	DF	Sum of Squares	Mean Square	F Ratio
Model	1	1.4985112	1.49851	12.4297
Error	5	0.6027922	0.12056	**Prob > F**
C. Total	6	2.1013034		0.0168*

Parameter Estimates

Term	Estimate	Std Error	t Ratio	Prob>|t|
Intercept	5.7531546	0.236587	24.32	<.0001*
day	-0.23134	0.065618	-3.53	0.0168*

Bivariate Fit of LnConc By day Oil=HFO, PAH=BIPHENYL

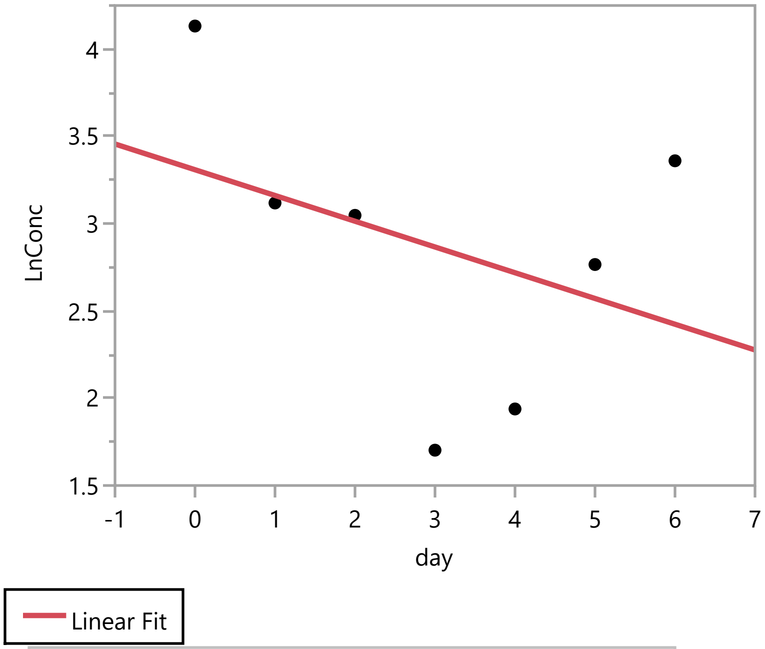

Linear Fit

LnConc = 3.3090965 - 0.1475178*day

Summary of Fit

RSquare	0.14615
RSquare Adj	-0.02462
Root Mean Square Error	0.843782
Mean of Response	2.866543
Observations (or Sum Wgts)	7

Analysis of Variance

Source	DF	Sum of Squares	Mean Square	F Ratio
Model	1	0.6093217	0.609322	0.8558
Error	5	3.5598423	0.711968	**Prob > F**
C. Total	6	4.1691639		0.3974

Parameter Estimates

Term	Estimate	Std Error	t Ratio	Prob>|t|
Intercept	3.3090965	0.574941	5.76	0.0022*
day	-0.147518	0.15946	-0.93	0.3974

Bivariate Fit of LnConc By day Oil=HFO, PAH=C1-CHRYSENES

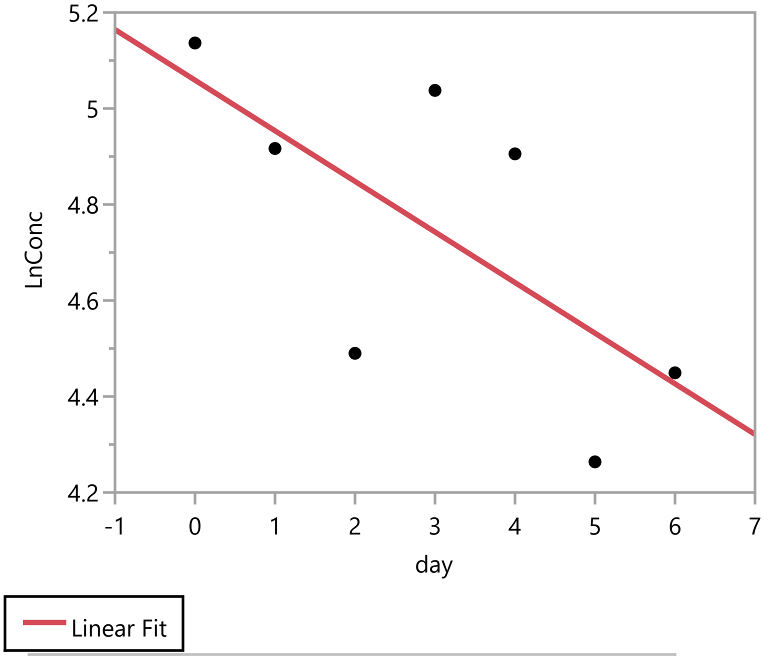

Linear Fit

LnConc = 5.059014 - 0.1054127*day

Summary of Fit

RSquare	0.458804
RSquare Adj	0.350565
Root Mean Square Error	0.270926
Mean of Response	4.742776
Observations (or Sum Wgts)	7

Analysis of Variance

Source	DF	Sum of Squares	Mean Square	F Ratio
Model	1	0.31113145	0.311131	4.2388
Error	5	0.36700461	0.073401	**Prob > F**
C. Total	6	0.67813606		0.0946

Parameter Estimates

Term	Estimate	Std Error	t Ratio	Prob>|t|
Intercept	5.059014	0.184605	27.40	<.0001*
day	-0.105413	0.0512	-2.06	0.0946

Bivariate Fit of LnConc By day Oil=HFO, PAH=C1-DIBENZOTHIOPHENES

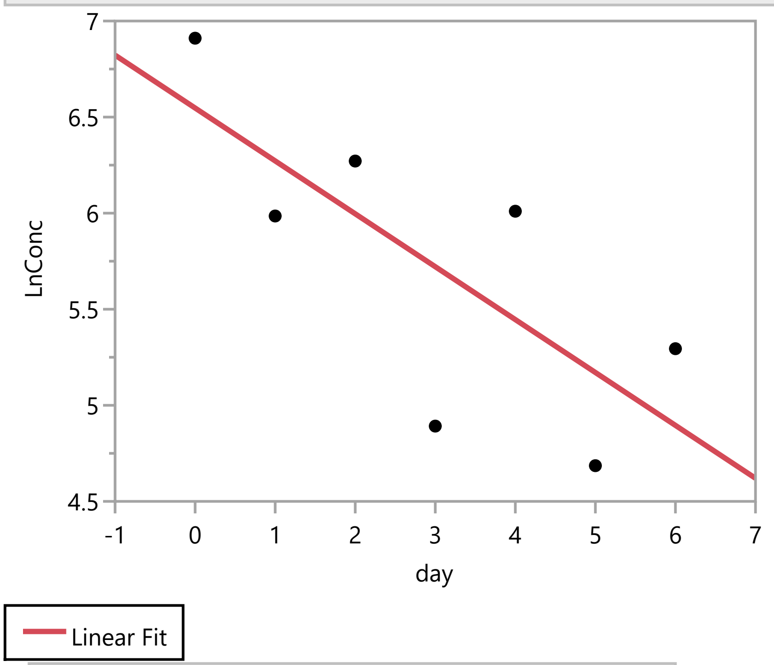

Linear Fit

LnConc = 6.547139 - 0.2752741*day

Summary of Fit

RSquare	0.556554
RSquare Adj	0.467864
Root Mean Square Error	0.581469
Mean of Response	5.721317
Observations (or Sum Wgts)	7

Analysis of Variance

Source	DF	Sum of Squares	Mean Square	F Ratio
Model	1	2.1217227	2.12172	6.2753
Error	5	1.6905289	0.33811	**Prob > F**
C. Total	6	3.8122516		0.0542

Parameter Estimates

Term	Estimate	Std Error	t Ratio	Prob>|t|
Intercept	6.547139	0.396204	16.52	<.0001*
day	-0.275274	0.109887	-2.51	0.0542

Bivariate Fit of LnConc By day
Oil=HFO, PAH=C1-FLUORANTHENES/PYRENES

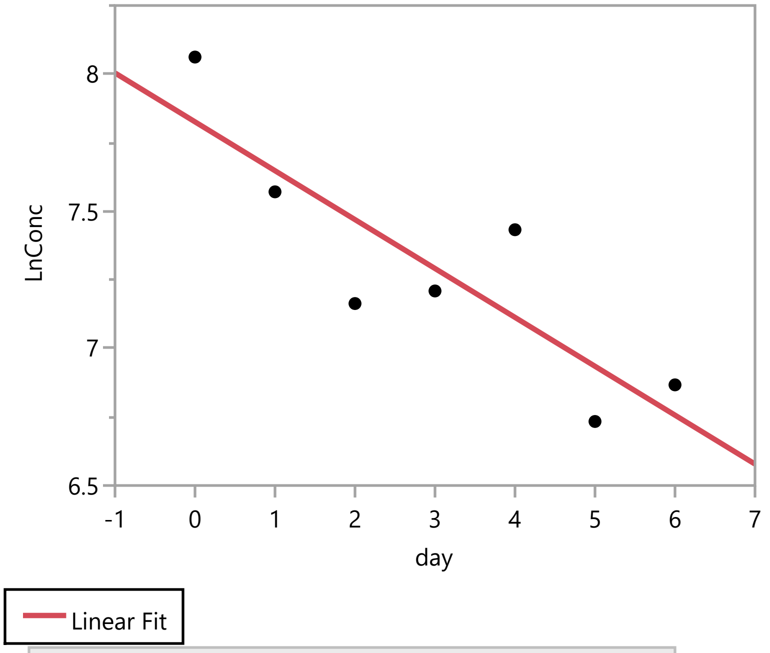

Linear Fit

LnConc = 7.8258706 - 0.1782375*day

Summary of Fit

RSquare	0.737336
RSquare Adj	0.684803
Root Mean Square Error	0.251745
Mean of Response	7.291158
Observations (or Sum Wgts)	7

Analysis of Variance

Source	DF	Sum of Squares	Mean Square	F Ratio
Model	1	0.8895214	0.889521	14.0357
Error	5	0.3168773	0.063375	**Prob > F**
C. Total	6	1.2063986		0.0133*

Parameter Estimates

Term	Estimate	Std Error	t Ratio	Prob>|t|
Intercept	7.8258706	0.171535	45.62	<.0001*
day	-0.178238	0.047575	-3.75	0.0133*

Bivariate Fit of LnConc By day Oil=HFO, PAH=C1-FLUORENES

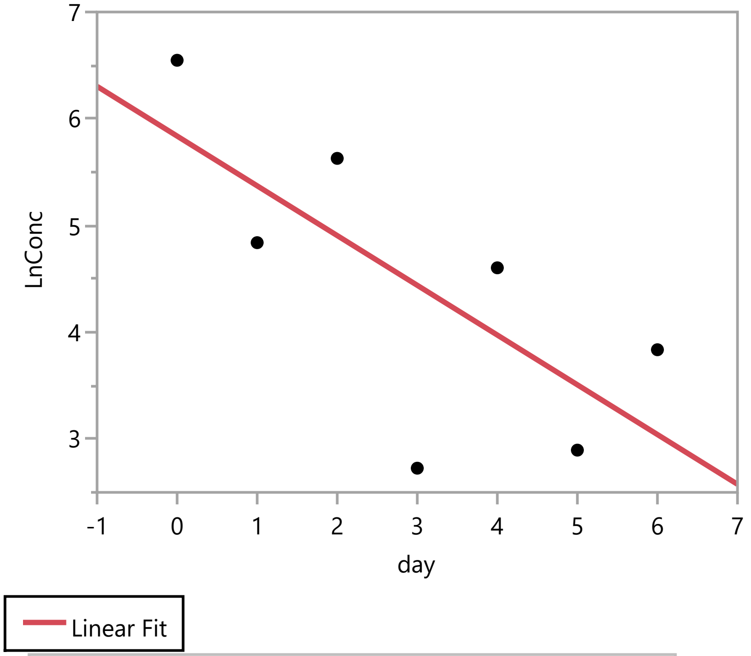

Linear Fit

LnConc = 5.8376478 - 0.4659697*day

Summary of Fit

RSquare	0.518379
RSquare Adj	0.422055
Root Mean Square Error	1.062871
Mean of Response	4.439739
Observations (or Sum Wgts)	7

Analysis of Variance

Source	DF	Sum of Squares	Mean Square	F Ratio
Model	1	6.079577	6.07958	5.3816
Error	5	5.648474	1.12969	**Prob > F**
C. Total	6	11.728051		0.0681

Parameter Estimates

Term	Estimate	Std Error	t Ratio	Prob>|t|
Intercept	5.8376478	0.724224	8.06	0.0005*
day	-0.46597	0.200864	-2.32	0.0681

Bivariate Fit of LnConc By day Oil=HFO, PAH=C1-NAPHTHALENES

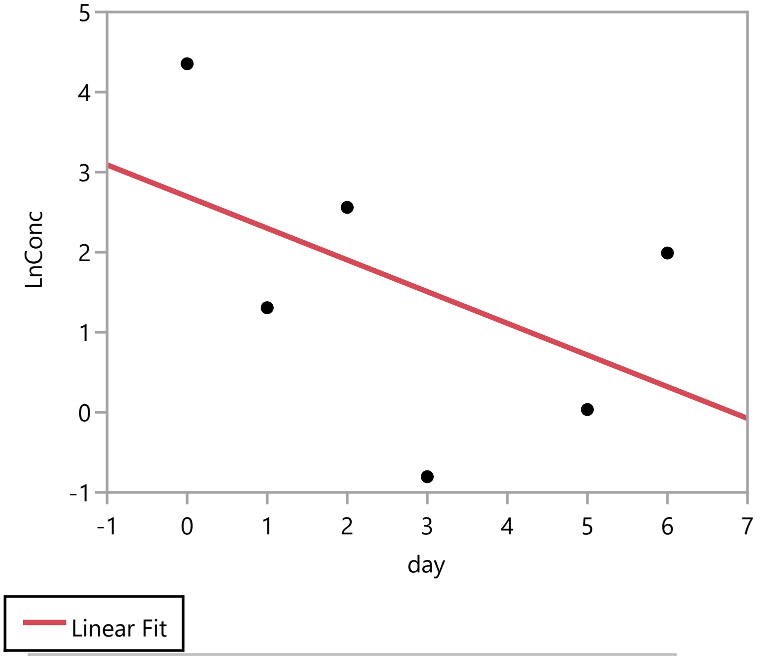

Linear Fit

LnConc = 2.6948656 - 0.3958593*day

Summary of Fit

RSquare	0.24777
RSquare Adj	0.059712
Root Mean Square Error	1.786484
Mean of Response	1.573264
Observations (or Sum Wgts)	6

Analysis of Variance

Source	DF	Sum of Squares	Mean Square	F Ratio
Model	1	4.204907	4.20491	1.3175
Error	4	12.766105	3.19153	**Prob > F**
C. Total	5	16.971012		0.3150

Parameter Estimates

Term	Estimate	Std Error	t Ratio	Prob>|t|
Intercept	2.6948656	1.219318	2.21	0.0916
day	-0.395859	0.344875	-1.15	0.3150

Bivariate Fit of LnConc By day
Oil=HFO, PAH=C1-NAPHTHOBENZOTHIOPHENES

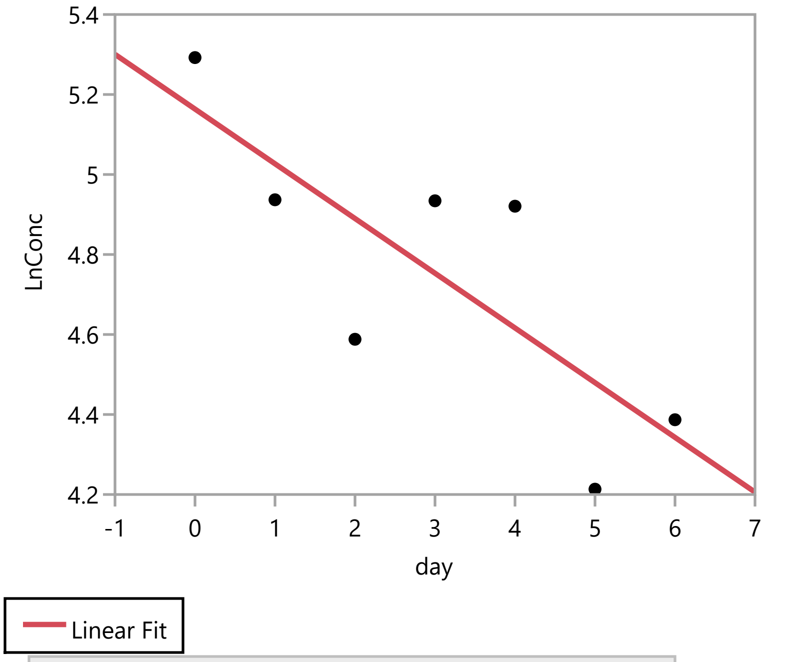

Linear Fit

LnConc = 5.1635976 - 0.1368046*day

Summary of Fit

RSquare	0.625243
RSquare Adj	0.550291
Root Mean Square Error	0.250637
Mean of Response	4.753184
Observations (or Sum Wgts)	7

Analysis of Variance

Source	DF	Sum of Squares	Mean Square	F Ratio
Model	1	0.52403367	0.524034	8.3420
Error	5	0.31409480	0.062819	Prob > F
C. Total	6	0.83812847		0.0343*

Parameter Estimates

Term	Estimate	Std Error	t Ratio	Prob>|t|
Intercept	5.1635976	0.17078	30.24	<.0001*
day	-0.136805	0.047366	-2.89	0.0343*

Bivariate Fit of LnConc By day Oil=HFO, PAH=C1-PHENANTHRENES/ANTHRACENES

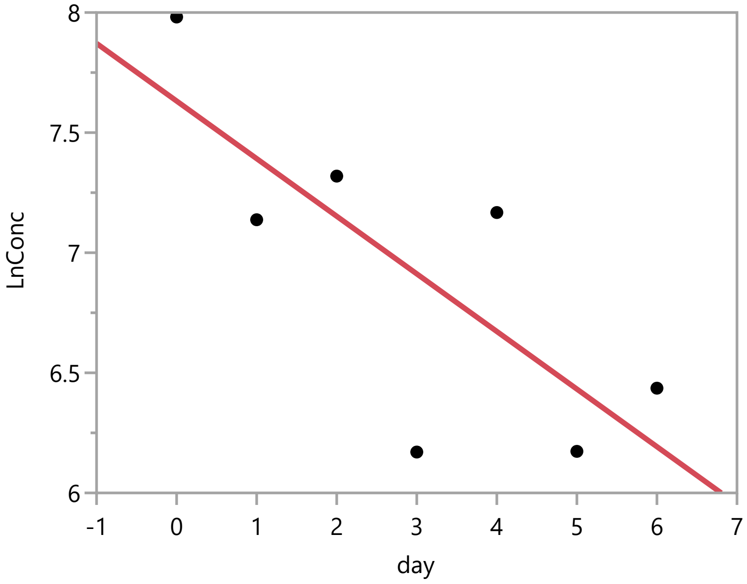

Linear Fit

LnConc = 7.6315546 - 0.2398531*day

Summary of Fit

RSquare	0.58622
RSquare Adj	0.503464
Root Mean Square Error	0.476863
Mean of Response	6.911995
Observations (or Sum Wgts)	7

Analysis of Variance

Source	DF	Sum of Squares	Mean Square	F Ratio
Model	1	1.6108263	1.61083	7.0837
Error	5	1.1369925	0.22740	**Prob > F**
C. Total	6	2.7478188		0.0448*

Parameter Estimates

Term	Estimate	Std Error	t Ratio	Prob>|t|
Intercept	7.6315546	0.324927	23.49	<.0001*
day	-0.239853	0.090119	-2.66	0.0448*

Bivariate Fit of LnConc By day
Oil=HFO, PAH=C2-BENZO(B)THIOPHENES

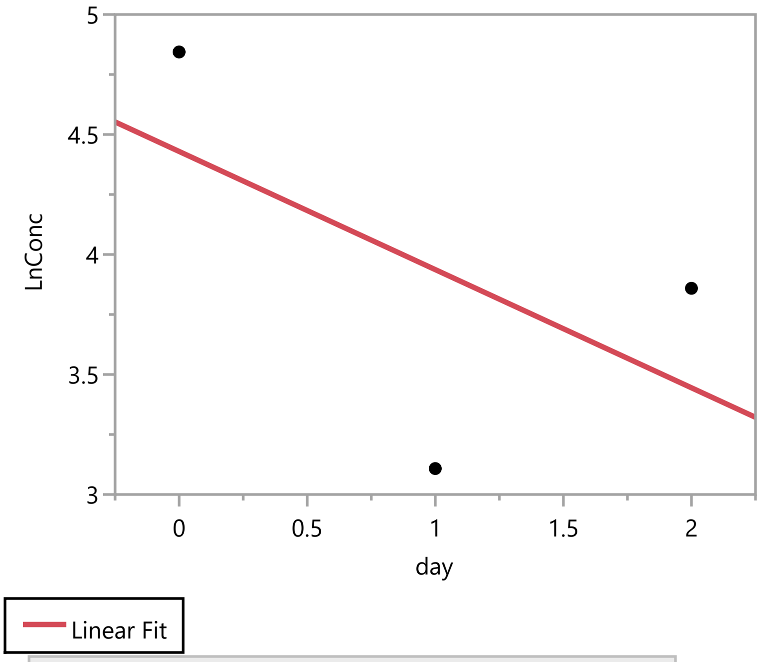

Linear Fit

LnConc = 4.4293721 - 0.4923654*day

Summary of Fit

RSquare	0.31991
RSquare Adj	-0.36018
Root Mean Square Error	1.015246
Mean of Response	3.937007
Observations (or Sum Wgts)	3

Analysis of Variance

Source	DF	Sum of Squares	Mean Square	F Ratio
Model	1	0.4848473	0.48485	0.4704
Error	1	1.0307253	1.03073	**Prob > F**
C. Total	2	1.5155726		0.6173

Parameter Estimates

Term	Estimate	Std Error	t Ratio	Prob>|t|
Intercept	4.4293721	0.926789	4.78	0.1313
day	-0.492365	0.717888	-0.69	0.6173

Bivariate Fit of LnConc By day Oil=HFO, PAH=C2-CHRYSENES

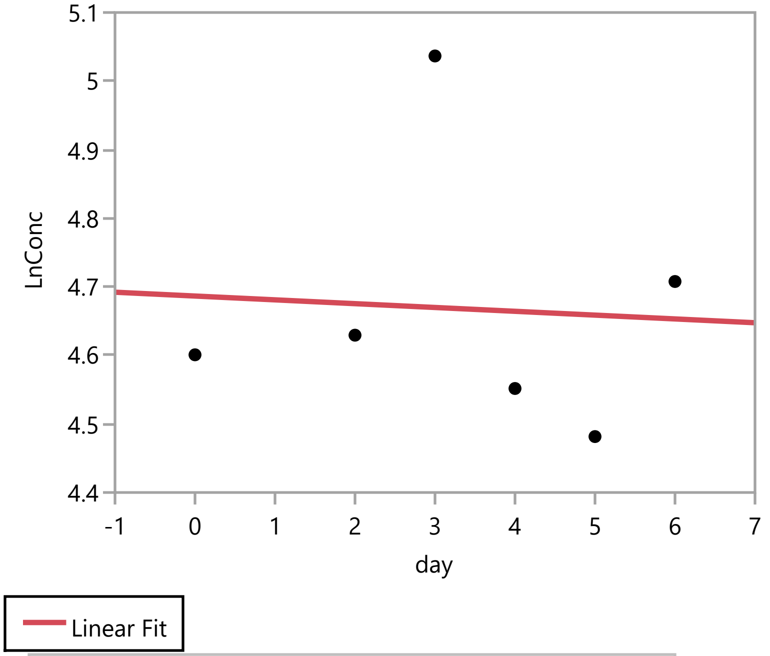

Linear Fit

LnConc = 4.6865152 - 0.0055653*day

Summary of Fit

RSquare	0.003769
RSquare Adj	-0.24529
Root Mean Square Error	0.218531
Mean of Response	4.667964
Observations (or Sum Wgts)	6

Analysis of Variance

Source	DF	Sum of Squares	Mean Square	F Ratio
Model	1	0.00072270	0.000723	0.0151
Error	4	0.19102235	0.047756	**Prob > F**
C. Total	5	0.19174505		0.9080

Parameter Estimates

Term	Estimate	Std Error	t Ratio	Prob>|t|
Intercept	4.6865152	0.175214	26.75	<.0001*
day	-0.005565	0.04524	-0.12	0.9080

Bivariate Fit of LnConc By day Oil=HFO, PAH=C2-DIBENZOTHIOPHENES

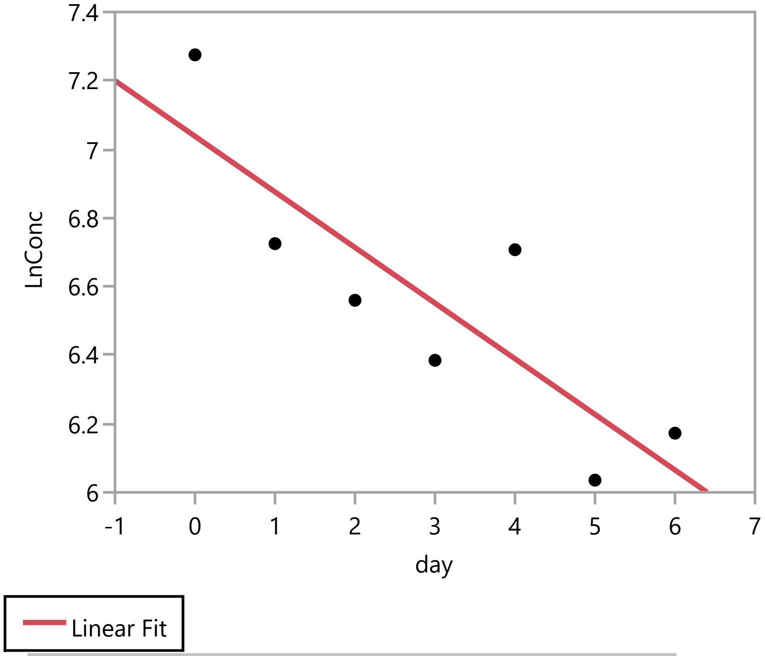

Linear Fit

LnConc = 7.0377843 - 0.1621162*day

Summary of Fit

RSquare	0.724595
RSquare Adj	0.669514
Root Mean Square Error	0.236515
Mean of Response	6.551436
Observations (or Sum Wgts)	7

Analysis of Variance

Source	DF	Sum of Squares	Mean Square	F Ratio
Model	1	0.7358862	0.735886	13.1551
Error	5	0.2796971	0.055939	**Prob > F**
C. Total	6	1.0155833		0.0151*

Parameter Estimates

Term	Estimate	Std Error	t Ratio	Prob>|t|
Intercept	7.0377843	0.161158	43.67	<.0001*
day	-0.162116	0.044697	-3.63	0.0151*

Bivariate Fit of LnConc By day
Oil=HFO, PAH=C2-FLUORANTHENES/PYRENES

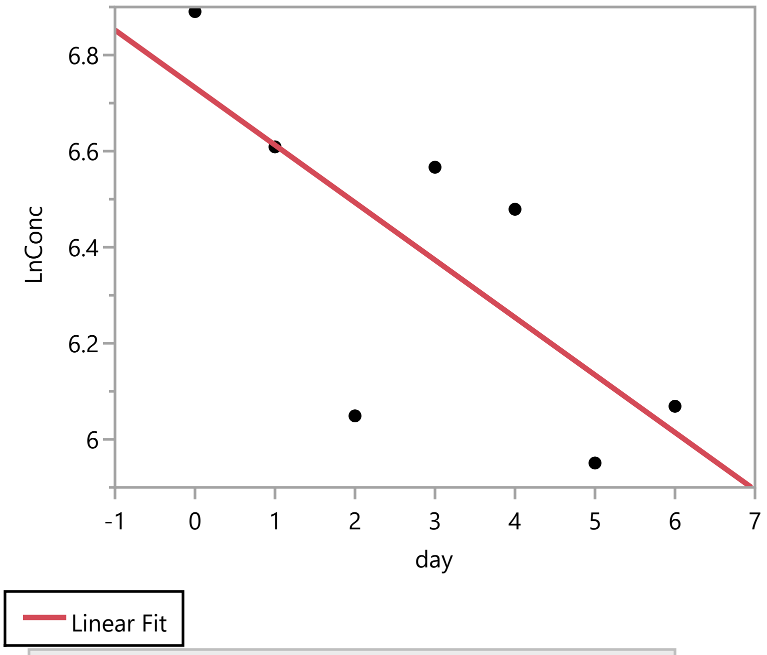

Linear Fit

LnConc = 6.732329 - 0.1196811*day

Summary of Fit

RSquare	0.536183
RSquare Adj	0.443419
Root Mean Square Error	0.263413
Mean of Response	6.373286
Observations (or Sum Wgts)	7

Analysis of Variance

Source	DF	Sum of Squares	Mean Square	F Ratio
Model	1	0.40105986	0.401060	5.7801
Error	5	0.34693116	0.069386	**Prob > F**
C. Total	6	0.74799101		0.0613

Parameter Estimates

Term	Estimate	Std Error	t Ratio	Prob>|t|
Intercept	6.732329	0.179485	37.51	<.0001*
day	-0.119681	0.04978	-2.40	0.0613

Bivariate Fit of LnConc By day Oil=HFO, PAH=C2-FLUORENES

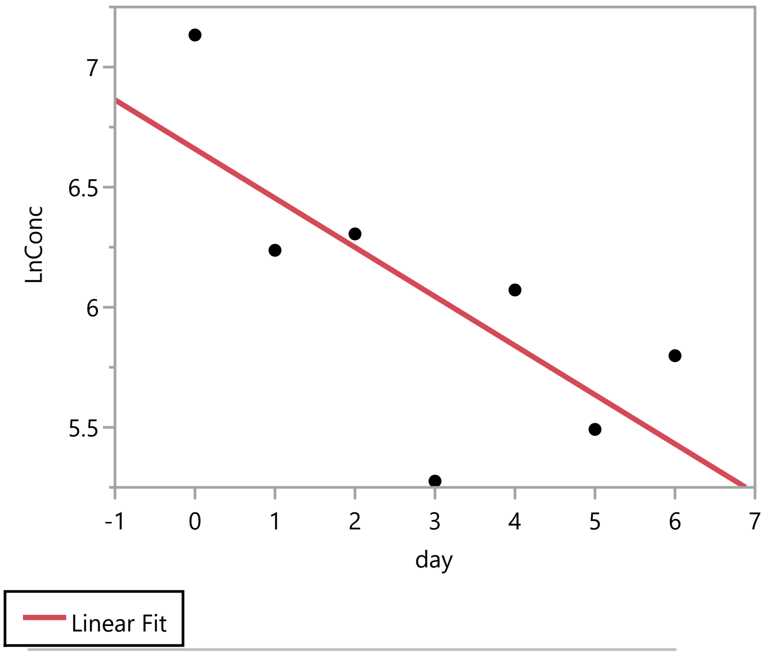

Linear Fit

LnConc = 6.6586822 - 0.2046624*day

Summary of Fit

RSquare	0.521497
RSquare Adj	0.425796
Root Mean Square Error	0.463926
Mean of Response	6.044695
Observations (or Sum Wgts)	7

Analysis of Variance

Source	DF	Sum of Squares	Mean Square	F Ratio
Model	1	1.1728278	1.17283	5.4492
Error	5	1.0761378	0.21523	**Prob > F**
C. Total	6	2.2489656		0.0668

Parameter Estimates

Term	Estimate	Std Error	t Ratio	Prob>|t|
Intercept	6.6586822	0.316112	21.06	<.0001*
day	-0.204662	0.087674	-2.33	0.0668

Bivariate Fit of LnConc By day Oil=HFO, PAH=C2-NAPHTHALENES

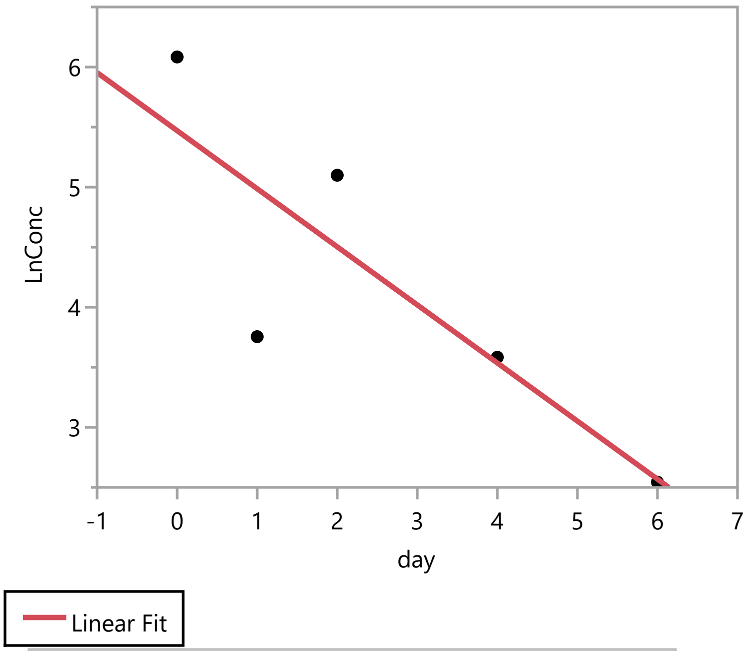

Linear Fit

LnConc = 5.4704334 - 0.4836723*day

Summary of Fit

RSquare	0.706745
RSquare Adj	0.608993
Root Mean Square Error	0.866415
Mean of Response	4.212885
Observations (or Sum Wgts)	5

Analysis of Variance

Source	DF	Sum of Squares	Mean Square	F Ratio
Model	1	5.4273824	5.42738	7.2300
Error	3	2.2520241	0.75067	**Prob > F**
C. Total	4	7.6794065		0.0745

Parameter Estimates

Term	Estimate	Std Error	t Ratio	Prob>|t|
Intercept	5.4704334	0.607343	9.01	0.0029*
day	-0.483672	0.17988	-2.69	0.0745

Bivariate Fit of LnConc By day
Oil=HFO, PAH=C2-NAPHTHOBENZOTHIOPHENES

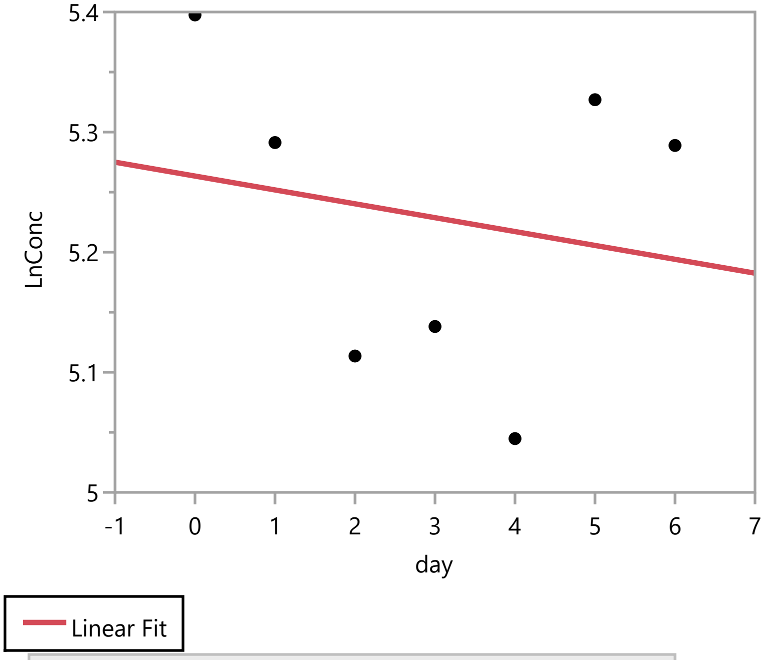

Linear Fit

LnConc = 5.263396 - 0.0115533*day

Summary of Fit

RSquare	0.036999
RSquare Adj	-0.1556
Root Mean Square Error	0.139483
Mean of Response	5.228736
Observations (or Sum Wgts)	7

Analysis of Variance

Source	DF	Sum of Squares	Mean Square	F Ratio
Model	1	0.00373739	0.003737	0.1921
Error	5	0.09727686	0.019455	**Prob > F**
C. Total	6	0.10101425		0.6795

Parameter Estimates

Term	Estimate	Std Error	t Ratio	Prob>|t|
Intercept	5.263396	0.095041	55.38	<.0001*
day	-0.011553	0.02636	-0.44	0.6795

Bivariate Fit of LnConc By day Oil=HFO, PAH=C2-PHENANTHRENES/ANTHRACENES

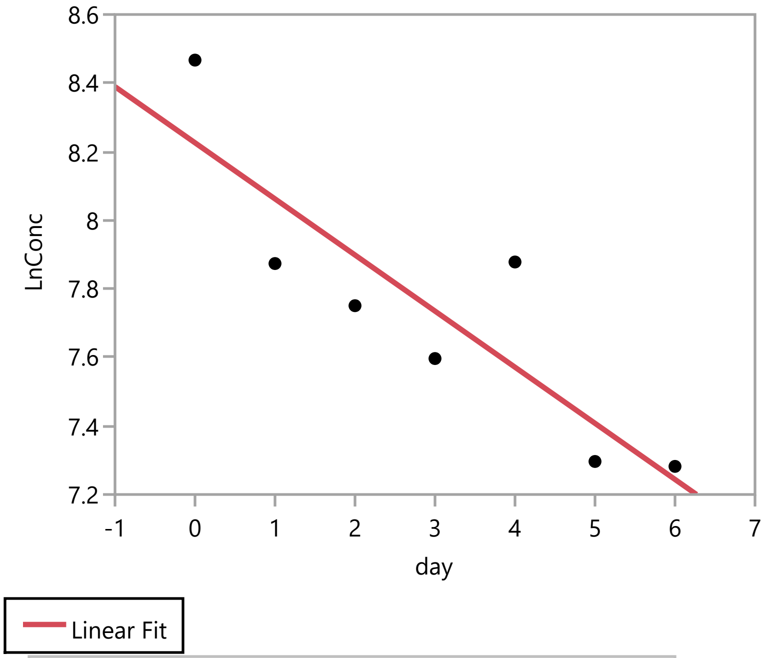

Linear Fit

LnConc = 8.2258483 - 0.163647*day

Summary of Fit

RSquare	0.755474
RSquare Adj	0.706569
Root Mean Square Error	0.22032
Mean of Response	7.734907
Observations (or Sum Wgts)	7

Analysis of Variance

Source	DF	Sum of Squares	Mean Square	F Ratio
Model	1	0.74984930	0.749849	15.4477
Error	5	0.24270543	0.048541	**Prob > F**
C. Total	6	0.99255473		0.0111*

Parameter Estimates

Term	Estimate	Std Error	t Ratio	Prob>|t|
Intercept	8.2258483	0.150123	54.79	<.0001*
day	-0.163647	0.041637	-3.93	0.0111*

Bivariate Fit of LnConc By day
Oil=HFO, PAH=C3-BENZO(B)THIOPHENES

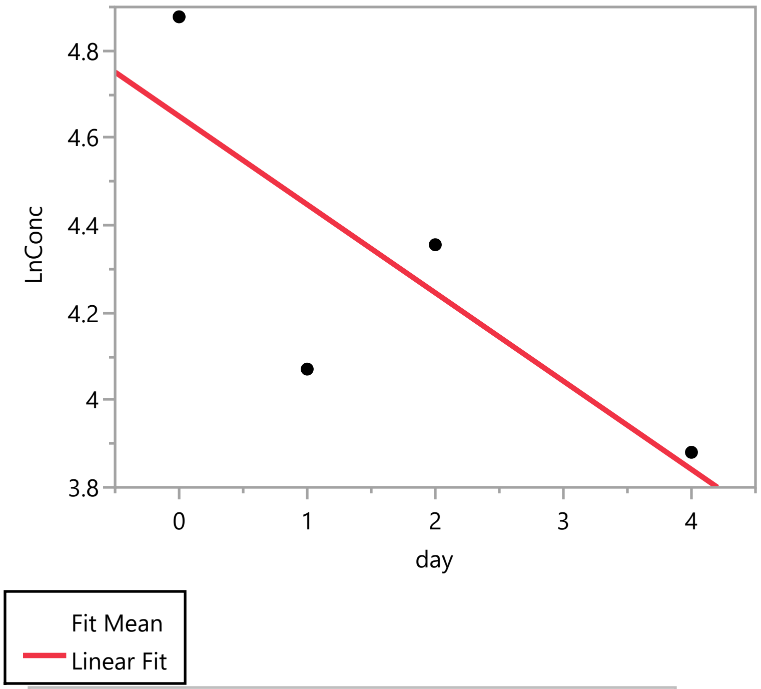

Linear Fit

LnConc = 4.6498807 - 0.202195*day

Summary of Fit

RSquare	0.63283
RSquare Adj	0.449245
Root Mean Square Error	0.322144
Mean of Response	4.296039
Observations (or Sum Wgts)	4

Analysis of Variance

Source	DF	Sum of Squares	Mean Square	F Ratio
Model	1	0.35772467	0.357725	3.4471
Error	2	0.20755295	0.103776	**Prob > F**
C. Total	3	0.56527761		0.2045

Parameter Estimates

Term	Estimate	Std Error	t Ratio	Prob>|t|
Intercept	4.6498807	0.249531	18.63	0.0029*
day	-0.202195	0.108904	-1.86	0.2045

Bivariate Fit of LnConc By day Oil=HFO, PAH=C3-DIBENZOTHIOPHENES

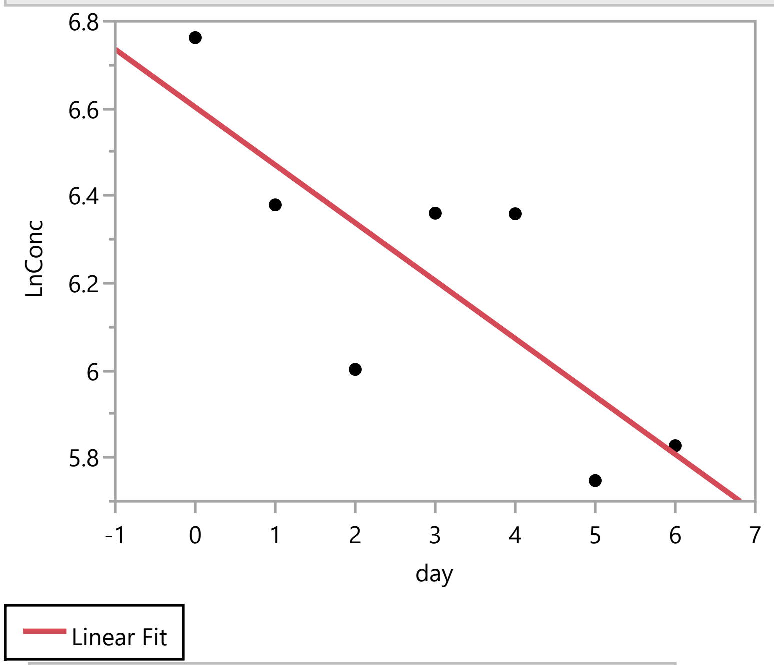

Linear Fit

LnConc = 6.6031469 - 0.1325939*day

Summary of Fit

RSquare	0.629414
RSquare Adj	0.555297
Root Mean Square Error	0.240765
Mean of Response	6.205365
Observations (or Sum Wgts)	7

Analysis of Variance

Source	DF	Sum of Squares	Mean Square	F Ratio
Model	1	0.49227218	0.492272	8.4921
Error	5	0.28983997	0.057968	**Prob > F**
C. Total	6	0.78211214		0.0332*

Parameter Estimates

Term	Estimate	Std Error	t Ratio	Prob>|t|
Intercept	6.6031469	0.164054	40.25	<.0001*
day	-0.132594	0.0455	-2.91	0.0332*

Bivariate Fit of LnConc By day
Oil=HFO, PAH=C3-FLUORANTHENES/PYRENES

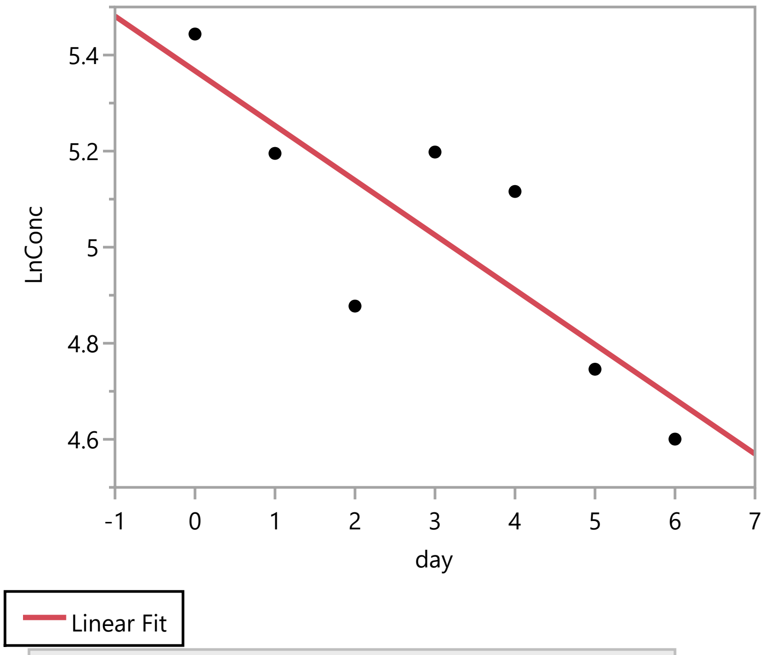

Linear Fit

LnConc = 5.3670429 - 0.1139248*day

Summary of Fit

RSquare	0.695577
RSquare Adj	0.634693
Root Mean Square Error	0.178352
Mean of Response	5.025268
Observations (or Sum Wgts)	7

Analysis of Variance

Source	DF	Sum of Squares	Mean Square	F Ratio
Model	1	0.36340826	0.363408	11.4245
Error	5	0.15904745	0.031809	**Prob > F**
C. Total	6	0.52245572		0.0197*

Parameter Estimates

Term	Estimate	Std Error	t Ratio	Prob>|t|
Intercept	5.3670429	0.121527	44.16	<.0001*
day	-0.113925	0.033705	-3.38	0.0197*

Bivariate Fit of LnConc By day Oil=HFO, PAH=C3-FLUORENES

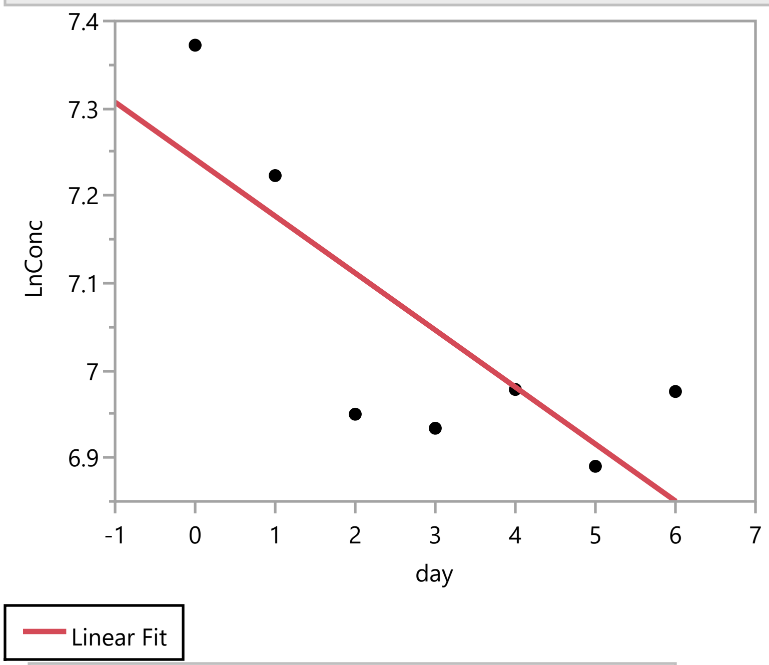

Linear Fit

LnConc = 7.2418784 - 0.0652465*day

Summary of Fit

RSquare	0.616039
RSquare Adj	0.539247
Root Mean Square Error	0.121896
Mean of Response	7.046139
Observations (or Sum Wgts)	7

Analysis of Variance

Source	DF	Sum of Squares	Mean Square	F Ratio
Model	1	0.11919879	0.119199	8.0222
Error	5	0.07429352	0.014859	**Prob > F**
C. Total	6	0.19349231		0.0366*

Parameter Estimates

Term	Estimate	Std Error	t Ratio	Prob>|t|
Intercept	7.2418784	0.083058	87.19	<.0001*
day	-0.065246	0.023036	-2.83	0.0366*

Bivariate Fit of LnConc By day Oil=HFO, PAH=C3-NAPHTHALENES

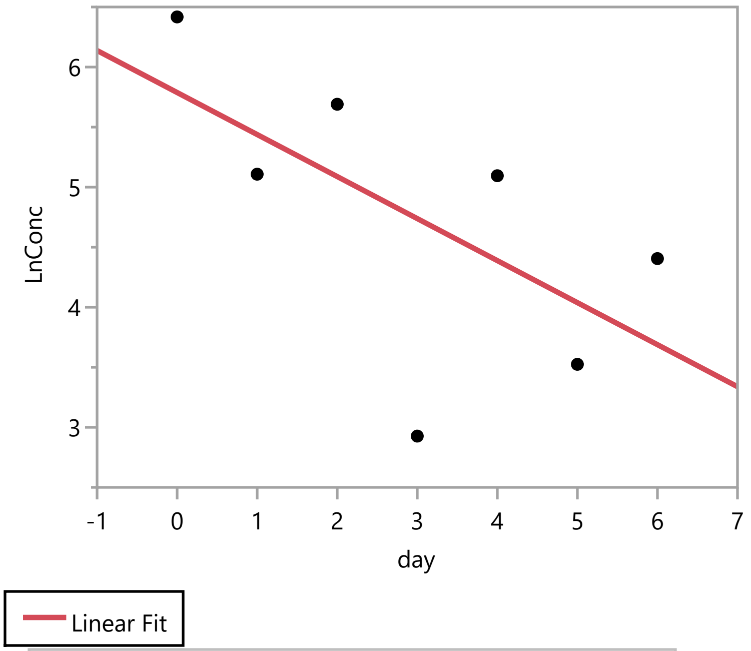

Linear Fit

LnConc = 5.787913 - 0.3499218*day

Summary of Fit

RSquare	0.387236
RSquare Adj	0.264683
Root Mean Square Error	1.041654
Mean of Response	4.738148
Observations (or Sum Wgts)	7

Analysis of Variance

Source	DF	Sum of Squares	Mean Square	F Ratio
Model	1	3.4284668	3.42847	3.1598
Error	5	5.4252174	1.08504	**Prob > F**
C. Total	6	8.8536842		0.1356

Parameter Estimates

Term	Estimate	Std Error	t Ratio	Prob>|t|
Intercept	5.787913	0.709768	8.15	0.0005*
day	-0.349922	0.196854	-1.78	0.1356

Bivariate Fit of LnConc By day Oil=HFO, PAH=C3-PHENANTHRENES/ANTHRACENES

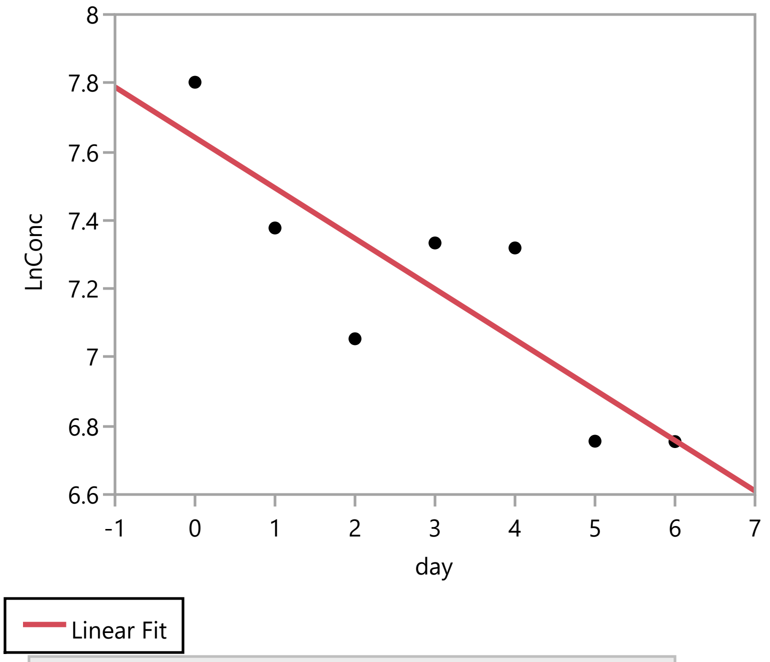

Linear Fit

LnConc = 7.6412571 - 0.1472545*day

Summary of Fit

RSquare	0.719438
RSquare Adj	0.663326
Root Mean Square Error	0.217611
Mean of Response	7.199493
Observations (or Sum Wgts)	7

Analysis of Variance

Source	DF	Sum of Squares	Mean Square	F Ratio
Model	1	0.60714922	0.607149	12.8214
Error	5	0.23677237	0.047354	**Prob > F**
C. Total	6	0.84392158		0.0159*

Parameter Estimates

Term	Estimate	Std Error	t Ratio	Prob>|t|
Intercept	7.6412571	0.148277	51.53	<.0001*
day	-0.147255	0.041125	-3.58	0.0159*

Bivariate Fit of LnConc By day Oil=HFO, PAH=C4-BENZO(B)THIOPHENES

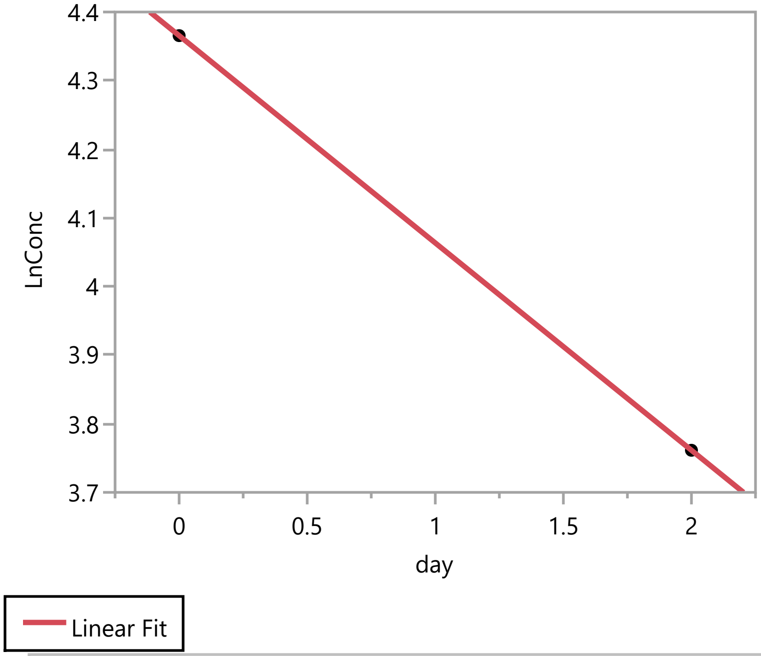

Linear Fit

LnConc = 4.3655879 - 0.3021226*day

Summary of Fit

RSquare	1
RSquare Adj	.
Root Mean Square Error	.
Mean of Response	4.063465
Observations (or Sum Wgts)	2

Analysis of Variance

Source	DF	Sum of Squares	Mean Square	MSE used	F Ratio
Model	1	0.18255609	0.182556	.	.
Error	0	0.00000000	.	**DFE used**	**Prob > F**
C. Total	1	0.18255609		.	.

Parameter Estimates

Term	Estimate	Std Error	t Ratio	Prob>|t|
Intercept	4.3655879	.	.	.
day	-0.302123	.	.	.

Bivariate Fit of LnConc By day Oil=HFO, PAH=C4-DIBENZOTHIOPHENES

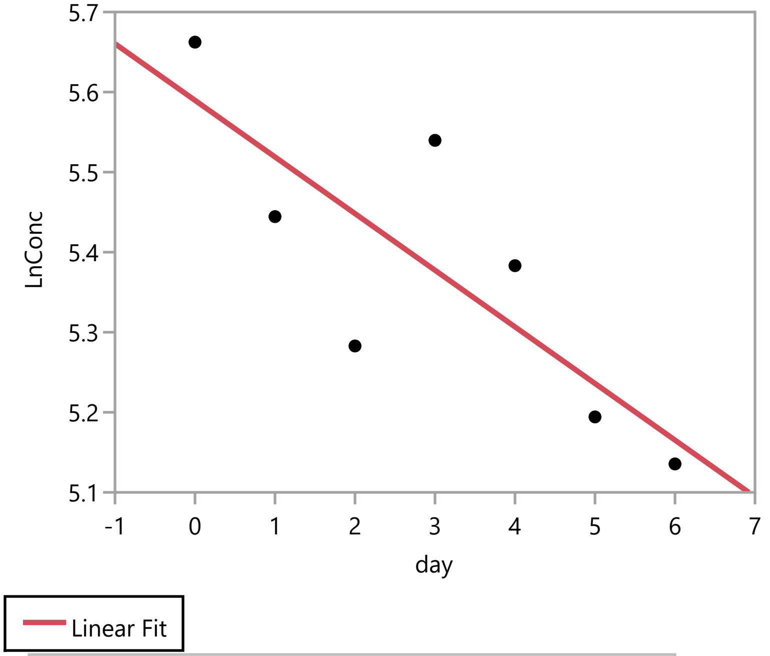

Linear Fit

LnConc = 5.5896788 - 0.0707357*day

Summary of Fit

RSquare	0.657553
RSquare Adj	0.589064
Root Mean Square Error	0.120799
Mean of Response	5.377472
Observations (or Sum Wgts)	7

Analysis of Variance

Source	DF	Sum of Squares	Mean Square	F Ratio
Model	1	0.14009906	0.140099	9.6008
Error	5	0.07296216	0.014592	**Prob > F**
C. Total	6	0.21306122		0.0269*

Parameter Estimates

Term	Estimate	Std Error	t Ratio	Prob>|t|
Intercept	5.5896788	0.082311	67.91	<.0001*
day	-0.070736	0.022829	-3.10	0.0269*

Bivariate Fit of LnConc By day
Oil=HFO, PAH=C4-FLUORANTHENES/PYRENES

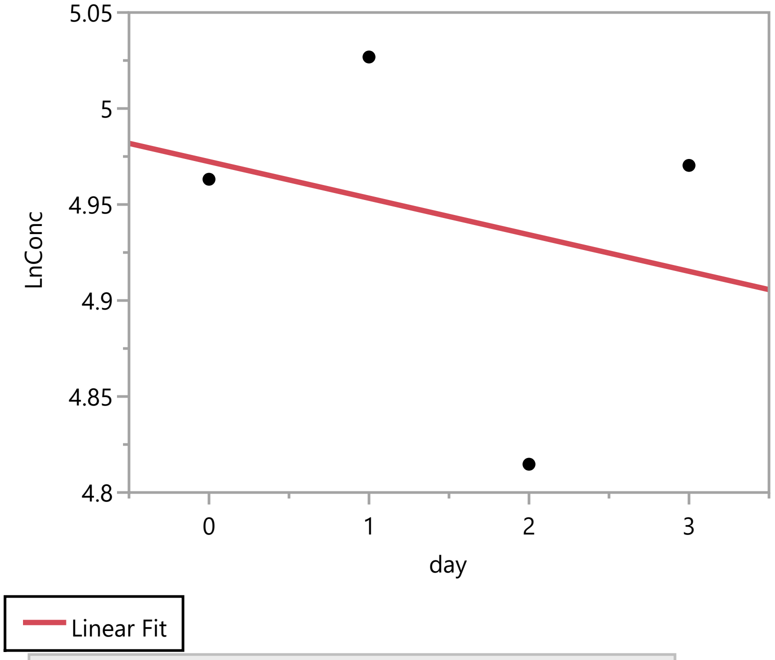

Linear Fit

LnConc = 4.9723325 - 0.0190457*day

Summary of Fit

RSquare	0.07363
RSquare Adj	-0.38956
Root Mean Square Error	0.106815
Mean of Response	4.943764
Observations (or Sum Wgts)	4

Analysis of Variance

Source	DF	Sum of Squares	Mean Square	F Ratio
Model	1	0.00181370	0.001814	0.1590
Error	2	0.02281898	0.011409	**Prob > F**
C. Total	3	0.02463268		0.7287

Parameter Estimates

Term	Estimate	Std Error	t Ratio	Prob>|t|
Intercept	4.9723325	0.089368	55.64	0.0003*
day	-0.019046	0.047769	-0.40	0.7287

Bivariate Fit of LnConc By day Oil=HFO, PAH=C4-NAPHTHALENES

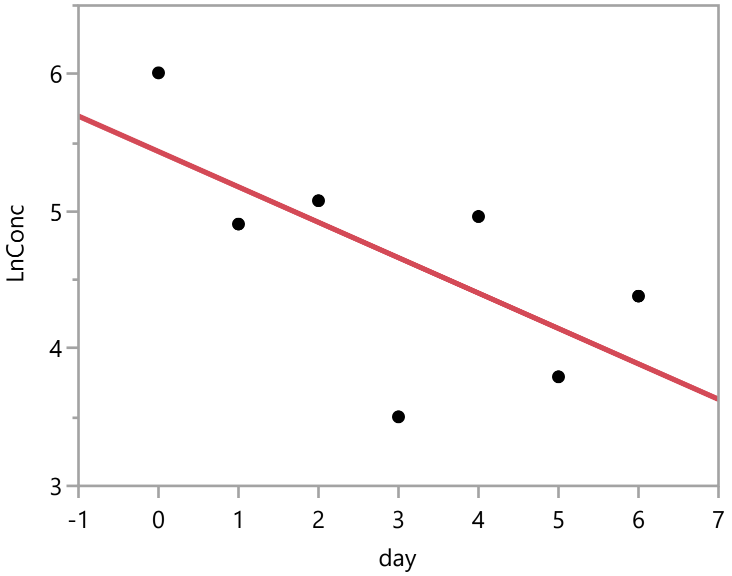

Linear Fit

LnConc = 5.4362077 - 0.2578708*day

Summary of Fit

RSquare	0.431885
RSquare Adj	0.318262
Root Mean Square Error	0.699891
Mean of Response	4.662595
Observations (or Sum Wgts)	7

Analysis of Variance

Source	DF	Sum of Squares	Mean Square	F Ratio
Model	1	1.8619259	1.86193	3.8010
Error	5	2.4492355	0.48985	**Prob > F**
C. Total	6	4.3111614		0.1087

Parameter Estimates

Term	Estimate	Std Error	t Ratio	Prob>|t|
Intercept	5.4362077	0.476895	11.40	<.0001*
day	-0.257871	0.132267	-1.95	0.1087

Bivariate Fit of LnConc By day Oil=HFO, PAH=C4-PHENANTHRENES/ANTHRACENES

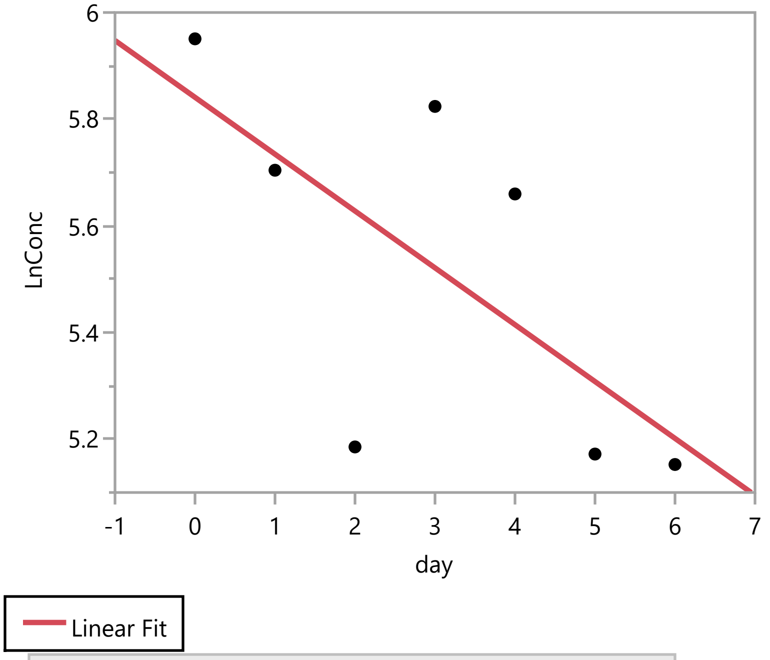

Linear Fit

LnConc = 5.8409848 - 0.1065869*day

Summary of Fit

RSquare	0.454866
RSquare Adj	0.34584
Root Mean Square Error	0.276126
Mean of Response	5.521224
Observations (or Sum Wgts)	7

Analysis of Variance

Source	DF	Sum of Squares	Mean Square	F Ratio
Model	1	0.31810143	0.318101	4.1721
Error	5	0.38122779	0.076246	**Prob > F**
C. Total	6	0.69932922		0.0966

Parameter Estimates

Term	Estimate	Std Error	t Ratio	Prob>|t|
Intercept	5.8409848	0.188148	31.04	<.0001*
day	-0.106587	0.052183	-2.04	0.0966

Bivariate Fit of LnConc By day
Oil=HFO, PAH=CHRYSENE/TRIPHENYLENE

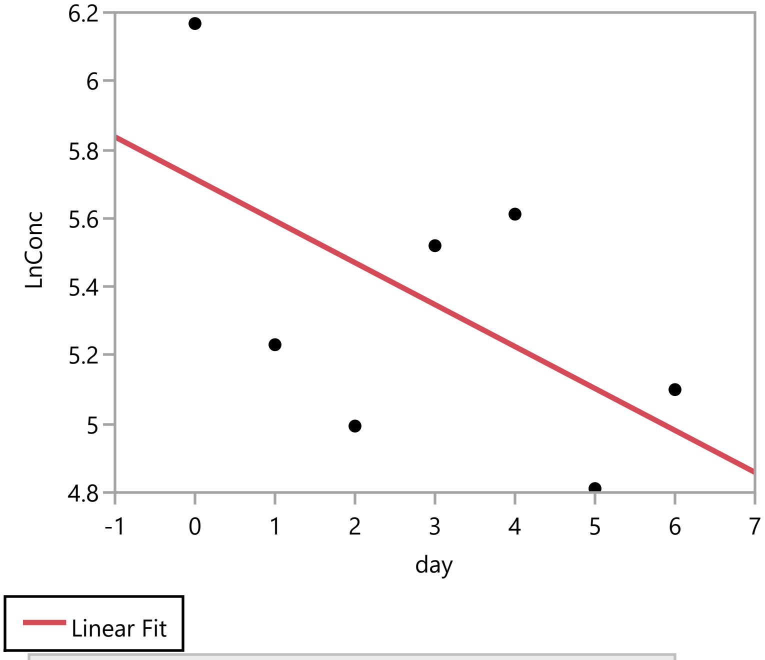

Linear Fit

LnConc = 5.7148598 - 0.122295*day

Summary of Fit

RSquare	0.332421
RSquare Adj	0.198905
Root Mean Square Error	0.410119
Mean of Response	5.347975
Observations (or Sum Wgts)	7

Analysis of Variance

Source	DF	Sum of Squares	Mean Square	F Ratio
Model	1	0.4187698	0.418770	2.4898
Error	5	0.8409875	0.168197	**Prob > F**
C. Total	6	1.2597573		0.1754

Parameter Estimates

Term	Estimate	Std Error	t Ratio	Prob>|t|
Intercept	5.7148598	0.279449	20.45	<.0001*
day	-0.122295	0.077505	-1.58	0.1754

Bivariate Fit of LnConc By day Oil=HFO, PAH=DIBENZOFURAN

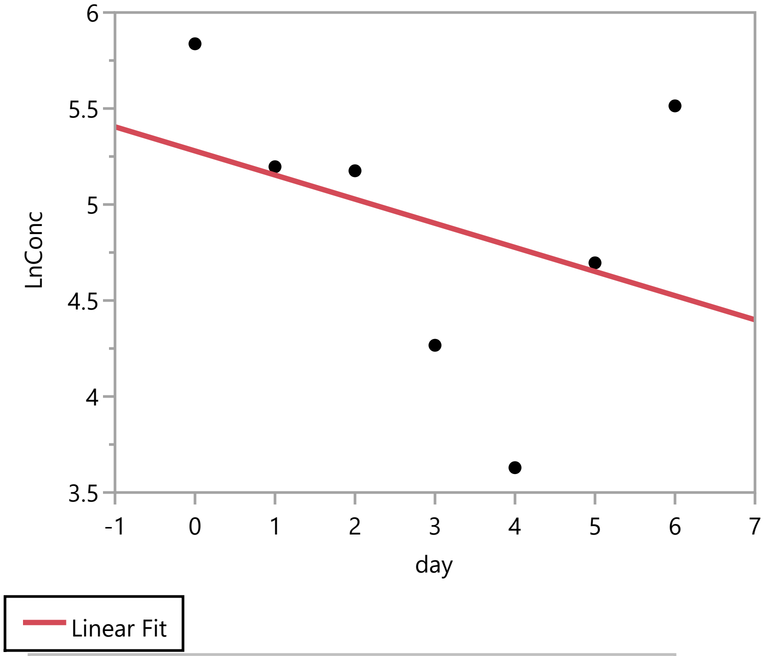

Linear Fit

LnConc = 5.2790636 - 0.1256346*day

Summary of Fit

RSquare	0.127151
RSquare Adj	-0.04742
Root Mean Square Error	0.778954
Mean of Response	4.90216
Observations (or Sum Wgts)	7

Analysis of Variance

Source	DF	Sum of Squares	Mean Square	F Ratio
Model	1	0.4419533	0.441953	0.7284
Error	5	3.0338495	0.606770	**Prob > F**
C. Total	6	3.4758028		0.4324

Parameter Estimates

Term	Estimate	Std Error	t Ratio	Prob>|t|
Intercept	5.2790636	0.530768	9.95	0.0002*
day	-0.125635	0.147209	-0.85	0.4324

Bivariate Fit of LnConc By day Oil=HFO, PAH=DIBENZOTHIOPHENE

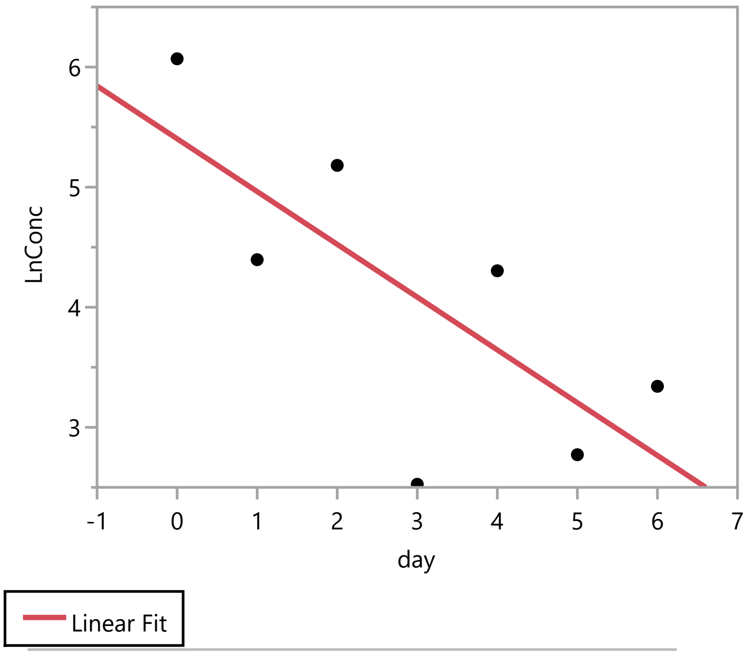

Linear Fit

LnConc = 5.4027846 - 0.4395622*day

Summary of Fit

RSquare	0.541531
RSquare Adj	0.449837
Root Mean Square Error	0.957101
Mean of Response	4.084098
Observations (or Sum Wgts)	7

Analysis of Variance

Source	DF	Sum of Squares	Mean Square	F Ratio
Model	1	5.4100168	5.41002	5.9059
Error	5	4.5802154	0.91604	**Prob > F**
C. Total	6	9.9902322		0.0594

Parameter Estimates

Term	Estimate	Std Error	t Ratio	Prob>|t|
Intercept	5.4027846	0.652155	8.28	0.0004*
day	-0.439562	0.180875	-2.43	0.0594

Bivariate Fit of LnConc By day Oil=HFO, PAH=FLUORANTHENE

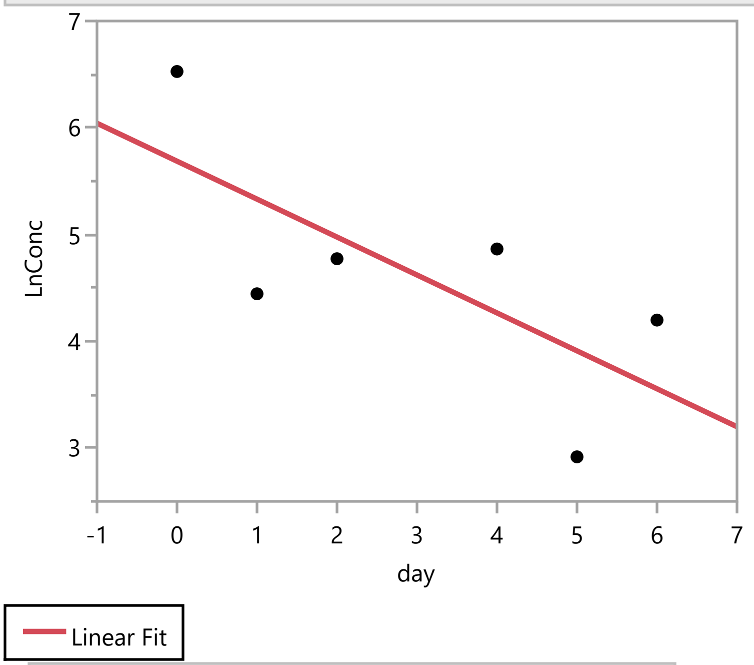

Linear Fit

LnConc = 5.6868602 - 0.355364*day

Summary of Fit

RSquare	0.517835
RSquare Adj	0.397294
Root Mean Square Error	0.907245
Mean of Response	4.620768
Observations (or Sum Wgts)	6

Analysis of Variance

Source	DF	Sum of Squares	Mean Square	F Ratio
Model	1	3.5359407	3.53594	4.2959
Error	4	3.2923764	0.82309	**Prob > F**
C. Total	5	6.8283171		0.1069

Parameter Estimates

Term	Estimate	Std Error	t Ratio	Prob>|t|
Intercept	5.6868602	0.633836	8.97	0.0009*
day	-0.355364	0.171453	-2.07	0.1069

Bivariate Fit of LnConc By day Oil=HFO, PAH=FLUORENE

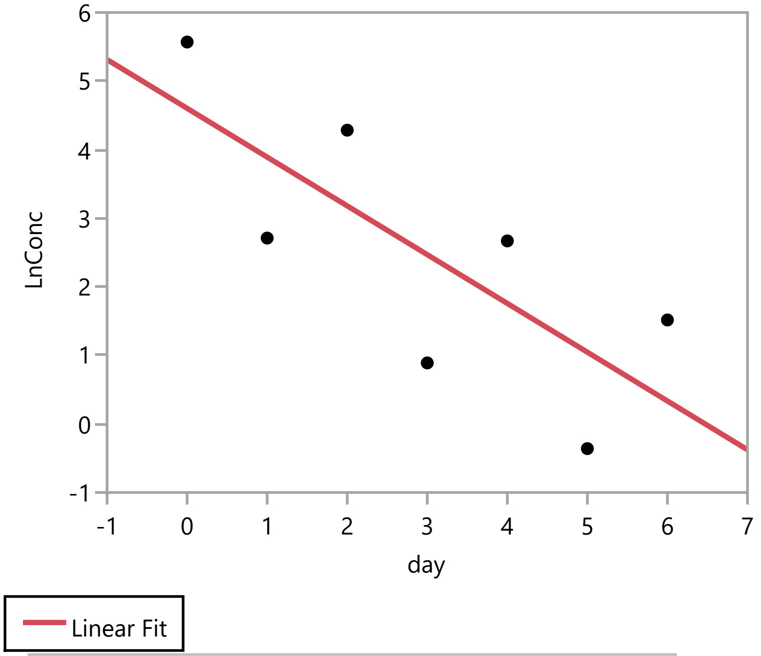

Linear Fit

LnConc = 4.6021204 - 0.7112665*day

Summary of Fit

RSquare	0.580308
RSquare Adj	0.49637
Root Mean Square Error	1.431404
Mean of Response	2.468321
Observations (or Sum Wgts)	7

Analysis of Variance

Source	DF	Sum of Squares	Mean Square	F Ratio
Model	1	14.165199	14.1652	6.9135
Error	5	10.244594	2.0489	**Prob > F**
C. Total	6	24.409793		0.0466*

Parameter Estimates

Term	Estimate	Std Error	t Ratio	Prob>|t|
Intercept	4.6021204	0.975338	4.72	0.0052*
day	-0.711266	0.27051	-2.63	0.0466*

Bivariate Fit of LnConc By day
Oil=HFO, PAH=NAPHTHOBENZOTHIOPHENE

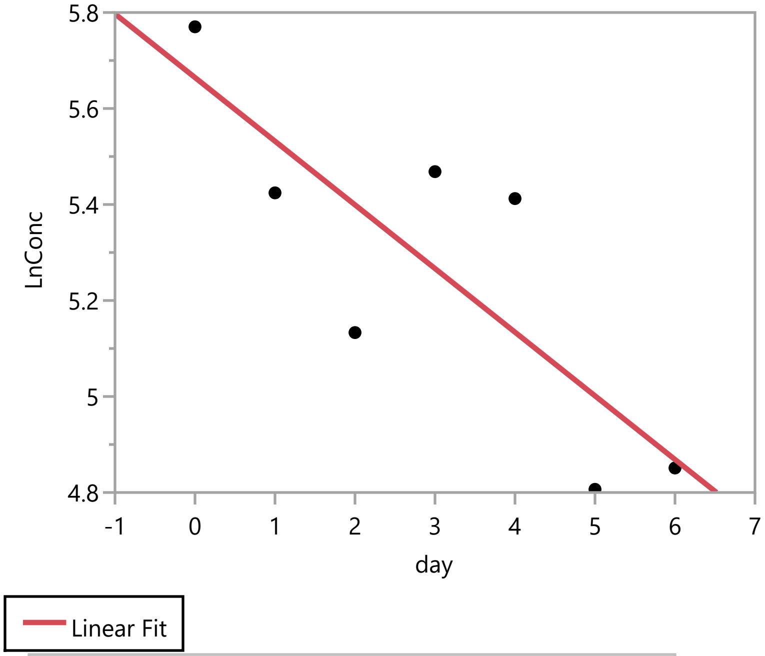

Linear Fit

LnConc = 5.6644603 - 0.1326253*day

Summary of Fit

RSquare	0.663301
RSquare Adj	0.595961
Root Mean Square Error	0.223608
Mean of Response	5.266585
Observations (or Sum Wgts)	7

Analysis of Variance

Source	DF	Sum of Squares	Mean Square	F Ratio
Model	1	0.49250500	0.492505	9.8500
Error	5	0.25000159	0.050000	**Prob > F**
C. Total	6	0.74250658		0.0257*

Parameter Estimates

Term	Estimate	Std Error	t Ratio	Prob>|t|
Intercept	5.6644603	0.152363	37.18	<.0001*
day	-0.132625	0.042258	-3.14	0.0257*

Bivariate Fit of LnConc By day Oil=HFO, PAH=PHENANTHRENE

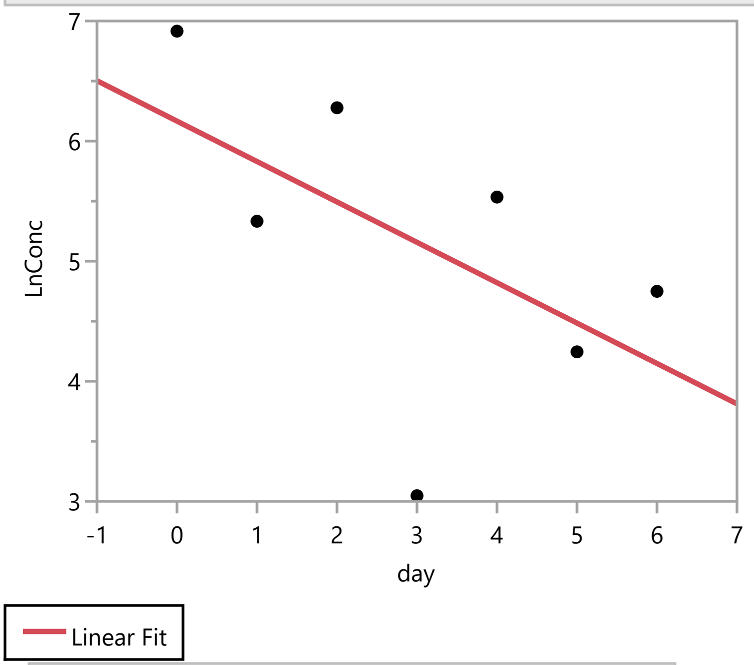

Linear Fit

LnConc = 6.1663535 - 0.3363496*day

Summary of Fit

RSquare	0.317691
RSquare Adj	0.18123
Root Mean Square Error	1.166468
Mean of Response	5.157305
Observations (or Sum Wgts)	7

Analysis of Variance

Source	DF	Sum of Squares	Mean Square	F Ratio
Model	1	3.1676686	3.16767	2.3281
Error	5	6.8032327	1.36065	**Prob > F**
C. Total	6	9.9709013		0.1876

Parameter Estimates

Term	Estimate	Std Error	t Ratio	Prob>|t|
Intercept	6.1663535	0.794814	7.76	0.0006*
day	-0.33635	0.220442	-1.53	0.1876

Bivariate Fit of LnConc By day Oil=HFO, PAH=PYRENE

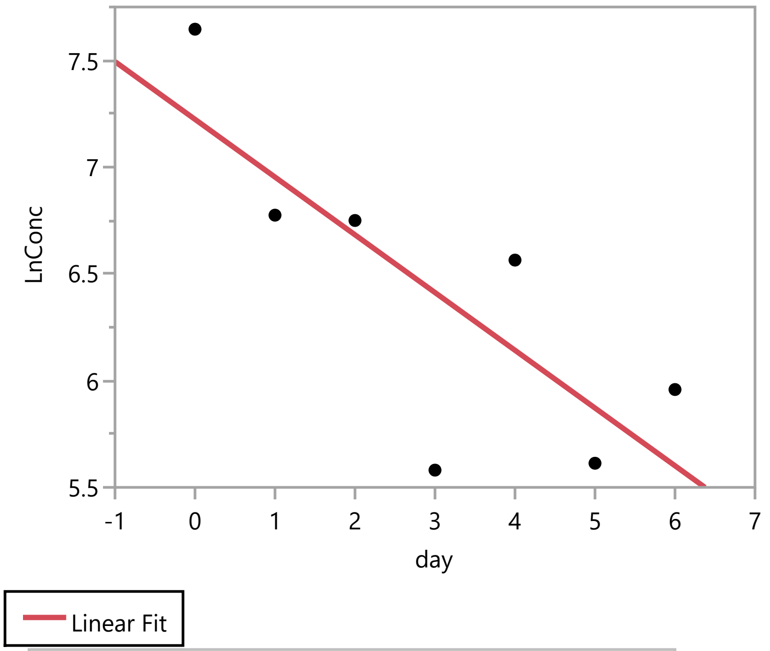

Linear Fit

LnConc = 7.2238796 - 0.2704497*day

Summary of Fit

RSquare	0.615522
RSquare Adj	0.538626
Root Mean Square Error	0.505819
Mean of Response	6.41253
Observations (or Sum Wgts)	7

Analysis of Variance

Source	DF	Sum of Squares	Mean Square	F Ratio
Model	1	2.0480057	2.04801	8.0046
Error	5	1.2792622	0.25585	**Prob > F**
C. Total	6	3.3272679		0.0367*

Parameter Estimates

Term	Estimate	Std Error	t Ratio	Prob>|t|
Intercept	7.2238796	0.344657	20.96	<.0001*
day	-0.27045	0.095591	-2.83	0.0367*
